# Steric Repulsion Induced Conformational Switch in Supramolecular Structures

**DOI:** 10.1002/chem.202103879

**Published:** 2021-12-02

**Authors:** Karolis Norvaiša, Sophie Maguire, Claire Donohoe, John E. O'Brien, Brendan Twamley, Ligia C. Gomes‐da‐Silva, Mathias O. Senge

**Affiliations:** ^1^ School of Chemistry, Chair of Organic Chemistry Trinity Biomedical Sciences Institute Trinity College Dublin The University of Dublin 152–160 Pearse Street D02 R590 Dublin 2 Ireland; ^2^ CQC, Coimbra Chemistry Center Department of Chemistry University of Coimbra 3000-435 Coimbra Portugal; ^3^ School of Chemistry Trinity College Dublin The University of Dublin D02 PN40 Dublin 2 Ireland; ^4^ Institute for Advanced Study (TUM-IAS) Focus Group – Molecular and Interfacial Engineering of Organic Nanosystems Technical University of Munich Lichtenbergstrasse 2a D-85748 Garching Germany

**Keywords:** Atropisomers, Crystallography, NMR, Porphyrinoids, Supramolecular Chemistry

## Abstract

Inspired by the rigidified architecture of ‘picket‐fence’ systems, we propose a strategy utilizing strain to impose intramolecular tension in already peripherally overcrowded structures leading to selective atropisomeric conversion. Employing this approach, tuneable shape‐persistent porphyrin conformations were acquired exhibiting distinctive supramolecular nanostructures based on the orientation of the peripheral groups. The intrinsic assemblies driven by non‐covalent bonding interactions form supramolecular polymers while encapsulating small molecules in parallel channels or solvent‐accessible voids. The developed molecular strain engineering methodologies combined with synthetic approaches have allowed the introduction of the pivalate units creating a highly strained molecular skeleton. Changes in the absorption spectrum indicated the presence of severe steric repulsions between the peripheral groups which were confirmed by single crystal X‐ray analysis. To release the steric strain introduced by the peripheral units, thermal equilibration strategies were used to selectively convert the most abundant atropisomer to the desirable minor one.

## Graphical abstract

Molecular strain engineering of dodecasubstituted picket‐fence porphyrins led us to develop a method for selective atropisomeric conversion based on the steric clashes. The tension rigidified architecture revealed distinct spectroscopic alterations with vibrant colorimetric changes making it a first case study where the porphyrin‐based photometric response is induced only by atropisomer configurations.

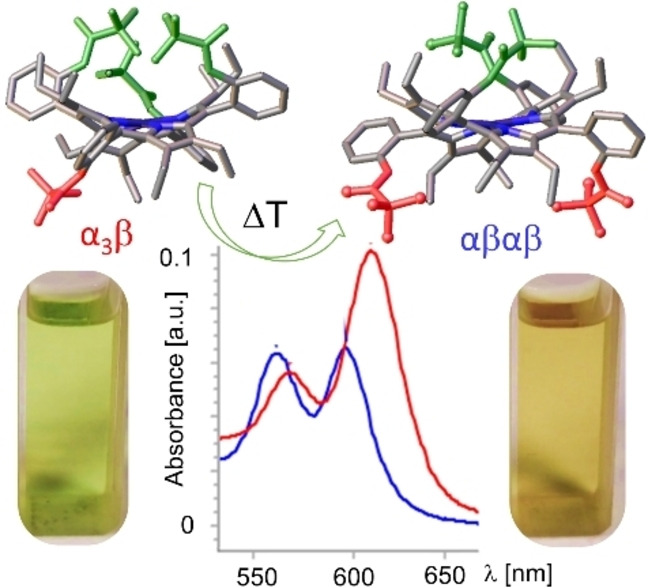

The molecular design of ‘picket‐fence’ porphyrins with peripheral pivalate (trimethylacetate) groups attracted our attention as superstructures bearing sterically hindered residues can create nonprotic cavities with fine control of the interactions with the core of the system.^[1]^ Employing such shape‐persistent molecular skeletons can lead to enhanced performance in certain situations such as solvent‐accessible cavity/channel formations,^[2]^ control of desired self‐assemblies,^[3]^ targeted atropisomeric enrichment methods,^[4]^ and tunable electrostatic configurations.^[5]^

While the conformational flexibility of tetrapyrrolic macrocycles has been found to play a vital role in controlling the function of proteins with porphyrin cofactors,^[6]^ for the development of synthetic methods to access artificial enzyme mimetic catalytic sites has only recently gained traction.^[7]^ The interplay of repulsive *peri*‐interactions was established to be a prerequisite to provoke a particular type of conformational distortion (saddle) to expose the inner pyrrolic units for the enzyme‐like catalytic active site. While new means of synthetic and electronic manipulation of the ring system to promote catalytic activity have introduced a handful of new out‐of‐shape superstructures, the remarkable flexibility of the porphyrin macrocycle continues to drive the search for novel ways to introduce more complex deformations.[^8^, ^9^]

Steric forces can undoubtedly play a critical role in the thermal stability of atropisomers^[10]^ and have been actively used to induce molecular transformations in stereochemistry (Figure 1a).^[11]^ While in porphyrin chemistry the general enrichment methods using weak coordination^[12]^ and thermal manipulations[^4^, ^13^] have been known for decades, the use of steric repulsion as a tool in selective atropisomeric conversion has been overlooked. Previously it has been shown that spatially demanding groups on the periphery of the aryl units can enhance the formation of the αβαβ atropisomer.[^4^, ^19^] Hence, we hypothesized that a ∼45° tilt of the phenyl groups due to the *peri*‐interactions could induce severe steric repulsion between the bulky groups at *ortho*‐positions which might be relaxed by thermally changing stereoconformation (Figure 1b). Such a powerful toolbox could be used to selectively transform the most abundant atropisomer (α_3_β) to the highly desirable (αβαβ) stereoisomer (Figure 1c).

**Figure 1:**
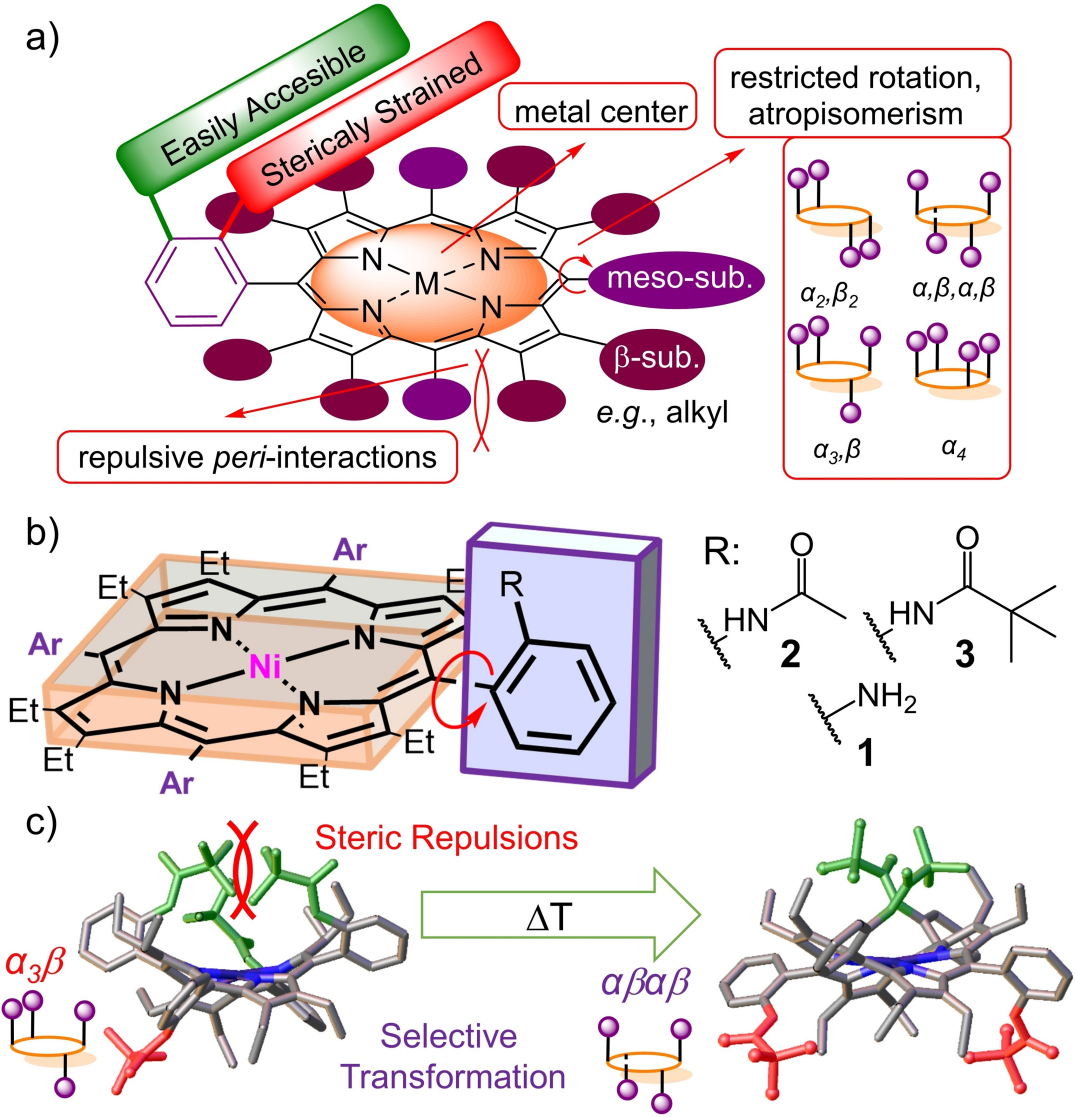
a) Graphical representation of easily accessible *meta*‐positions^[9]^ and sterically challenging *ortho*‐positional for substitution in nonplanar porphyrins; b) ‘Picket fence” porphyrins discussed in the following study; c) The illustration of the steric repulsion‐induced conformational switch.

The synthesis of nonplanar ‘picket‐fence’ porphyrin systems presents a formidable challenge due to the sterically overcrowded superstructure which restricts access to porphyrin phenyl *ortho*‐positions.^[9]^ To address the common issues associated with introducing picket fence residues to *ortho*‐positions, four main molecular engineering strategies were exploited (Figure 1a): 1) dodecasubstitution to induce repulsive *peri*‐interactions for defined saddle deformation; 2) restricted C−C bond rotation for ultimate control of the shape‐persistent conformations in different atropisomers; 3) the presence of a metal center simplifies reaction monitoring by eliminating possible inner N−*H* tautomerism; 4) substitution with less bulky reagents (e. g., acetyl chloride) to minimize steric bulk around the *ortho*‐positions.

The acetylated nonplanar porphyrins α_3_β‐**2**; α_4_‐**2** and α_2_β_2_‐**2** were prepared from corresponding {5,10,15,20‐tetrakis(2‐aminophenyl)‐2,3,7,8,12,13,17,18‐octaethylporphyrinato}nickel(II) (**1**) atropisomers^[15]^ using an excess of acetyl chloride (Scheme S1). While the synthesis of α_4_‐**2** and α_2_β_2_‐**2** required over 100 equiv. of acetyl chloride, full conversion to α_3_β‐**2** required 40 equivalents of acetyl chloride, only one‐third of the amount required for the previous two atropisomers, indicating a more accessible substitution pattern.

Next, we explored the thermal stability of α_3_β‐**2** by variable temperature ^1^H NMR (VT NMR) in *d_6_*‐DMSO. Initially, the sample appeared to be thermally stable when heated to 60 °C (Figure S15); however, upon raising the temperature to 100 °C new spectral lines were observed which indicate thermal interconversion (Figure S16). In order to further investigate the atropisomeric transformations, the sample was heated to 150 °C for 15 h (Note, full conversion was already observed at 100 °C after 1 h, Figure S17) converting to αβαβ‐**2** in 62 % atropisomeric purity with minor fractions of α_2_β_2_‐**2** (13 %) and α_3_β‐**2** (25 %) (ratios identified by HPLC analysis, see Figure S21). All of the isolated **2** atropisomers were recrystallized and their structure confirmed via X‐ray crystallography, contributing to the scarce database of full atropisomeric porphyrin families (Figure 2, Figure S1).

**Figure 2:**
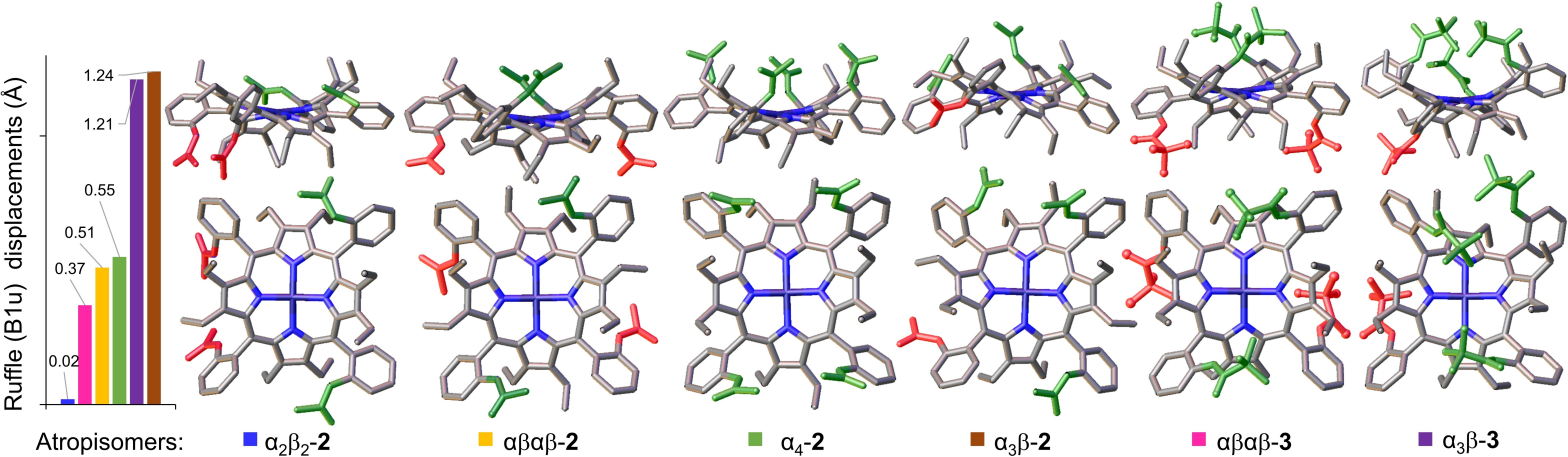
Isolated molecular structures of the atropisomers discussed in this study (Figure 1b), in green – peripheral groups (acetyl (**2**) or pivaloyl (**3**)) above the macrocycle plane, in red – below the plane. Hydrogen atoms and solvent molecules were omitted for clarity. On the left, a chart of the ruffle distortion obtained from out‐of‐plane normal‐coordinate structural decomposition (NSD) (see Figure S2 for full NSD analysis).

The basis for the incomplete conversion of α_3_β‐**2** to αβαβ‐**2** can be explained through the X‐ray single‐crystal analysis (Figure 2). The structure of α_3_β‐**2** indicates a high strain energy state due to the strong ruffling (B_1u_) profile introduced by the steric repulsion between the acetyl groups in addition to the dominant saddle (B_2u_) deformation (Figure S2, Video S1), making it the least thermally stable conformation. Evidently, αβαβ‐**2** is the most favorable conformation as sterically repulsive groups are divided equally below and above the plane. The high thermal stability of αβαβ‐**2** was indicated by VT NMR as no spectral changes were observed upon cooling down to 25 °C after heating to 99 °C (Figure S18). This was confirmed by HPLC analysis after heating the sample for 1 h at 100 °C (Figure S22). Nevertheless, a small amount of α_2_β_2_‐**2** has also been acquired upon thermal α_3_β‐**2** conversion. Examination of the α_2_β_2_‐**2** structure found dominant intermolecular H‐bonding features which result in minimal ruffling distortion of the macrocycle and lower the overall strain energy (Figure 4; Figure S12).

NMR analyses of the **2** atropisomers were performed in *d_6_*‐DMSO as this provided the most well‐resolved spectra in comparison to other deuterated solvents (Figure S19). As expected, the αβαβ‐**2** and α_4_‐**2** atropisomers showed the most resolved spectra with well‐defined aromatic and aliphatic regions attributed to the high symmetry and the low probability of stacking forming an intermolecular H⋅⋅⋅H network (Figure 3). The characteristic amide (N−H) resonance signals of αβαβ‐**2** are shifted further downfield in comparison to α_4_‐**2** contributing to the more accessible solvent binding pockets for potential hydrogen bonding interactions. The number of resonance signals in α_3_β‐**2** is noticeably greater as a result of the unsymmetrical nature of the rotamer.^[17]^ The ^1^H NMR spectrum of α_2_β_2_‐**2** was found to be the least resolved with very broad resonance signals. This indicated the formation of intermolecular hydrogen bonding networks, as was observed in the X‐ray structural analysis (Figure 4; Figure S12). The resolution of the ^1^H NMR spectra could be greatly improved by performing the measurements at higher temperatures, due to the disruption of the intermolecular stacking interactions (Figure S20).

**Figure 3:**
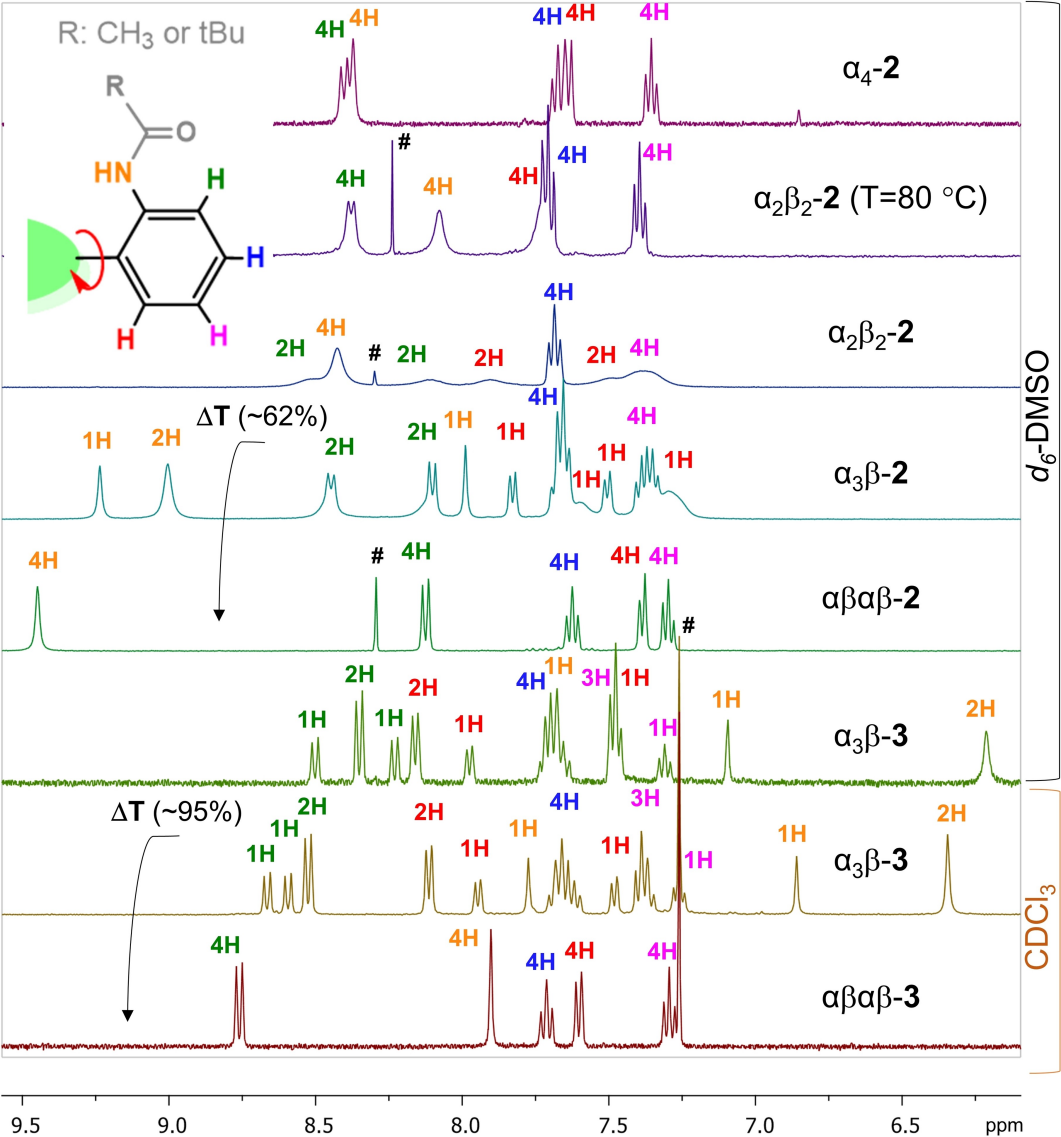
^1^H NMR spectra of aromatic regions in atropisomers **2** and **3** (*d_6_*‐DMSO or CDCl_3_); ΔT represents atropisomeric thermal interconversion; Hashtag symbol marks chloroform signal.

**Figure 4:**
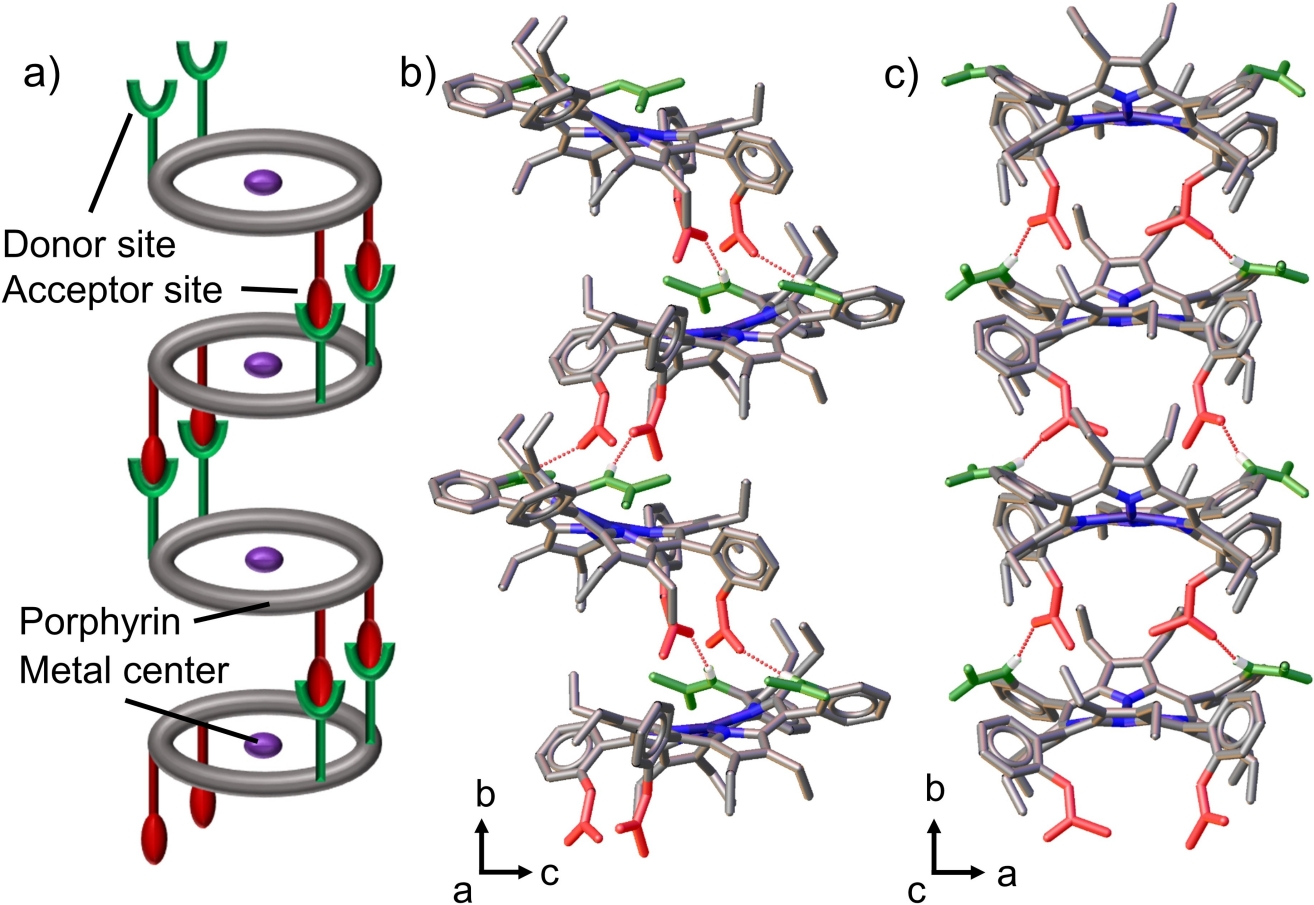
a) Schematic illustration of the intermolecular packing observed in α_2_β_2_‐**2**; b) and c) structural packing representation at different viewing angles. Non‐essential hydrogen atoms and solvent molecules were omitted for clarity. Red represents the amide groups as hydrogen‐bond acceptors, while green represents the hydrogen bond donor groups.

In order to enable the assembly of the interlinked superstructure, the structural model requires shape‐persistent dual functionality, wherein one side acts as acceptor, while the other acts as a donor. The precisely defined α_2_β_2_‐**2** structure, arrayed with opposing aryl group angles, satisfies such a critical design requirement. Inwardly facing acetyl groups form H⋅⋅⋅H acceptor sites, whereas on the other side of the macrocycle, outwardly orientated amide groups create donating pockets (Figure 4; Figure S12). While α_3_β‐**2**, α_4_‐**2** and α_2_β_2_‐**2** form solvent‐accessible voids with different sized cavities (α_3_β‐**2**>α_4_‐**2**>α_2_β_2_‐**2**), αβαβ‐**2** constructs solvent‐accessible parallel channels (Figure S13). Such tuneable supramolecular arrangements, depending on the orientation of the peripheral coordinating units, are a particularly important consideration for composing sophisticated molecular ensembles where control of intermolecular distances is important^[17]^ or selective encapsulation of small molecules is required.^[2]^

In line with the observations of α_3_β‐**2** thermal conversion to αβαβ‐**2**, we hypothesized that the use of even bulkier peripheral substituents could tune the thermal transformation selectively towards the αβαβ conformation. To test this hypothesis, we decided to attempt the introduction of the original^[1]^ “picket fence” reagent (pivaloyl chloride) on the α_3_β‐**1** scaffold From the previous studies with α_4_‐**1**, it was expected that substitution at the *ortho*‐positions with pivaloyl chloride would be a challenging task.^[9]^ As anticipated, this required the addition of 120 equiv. of pivaloyl chloride three‐fold greater than required for the synthesis of α_3_β‐**2**. In chloroform, the trisubstituted product was predominantly formed (Figure S23). To propel the reaction to tetra‐substitution, the reaction mixture was heated to 60 °C for 2 h (longer heat exposure might induce thermal equilibration) successfully yielding α_3_β‐**3**. The obtained structure of α_3_β‐**3** shows a high ruffling profile comparable to α_3_β‐**2** which suggests the high strain energy allows for easy thermal interconversion and offers a novel way of inducing ruffling. (Figure 2). Typically such immense ruffling deformations with dominant saddling is observed in unsymmetrical dodecasubstituted porphyrins (Figure S9),^[18]^ on protonation of formerly dominant ruffled systems (Figure S10),^[19]^ or guest‐assisted sterically hindered porphyrins (Figure S11).^[20]^

In order to test thermal convergence and issue the first report of selective one‐pot synthesis and simultaneous conversion from α_3_β‐**1** to αβαβ‐**3**, we have designed a synthetic protocol that includes an excess of pivaloyl chloride combined with *N,N‐*diisopropylethylamine in dichlorobenzene stirred at 120 °C for 15 h. Despite the moderate yield obtained (53 %) due to the formation of a tri‐substituted product and the extensive purification required, αβαβ‐**3** was isolated in 95 % atropisomeric excess (5 % identified as α_3_β‐**3**). This confirms the hypothesis that with the increasing bulkiness of the peripheral units, steric clashes are tremendously more dominant, resulting in the stereoselective conversion to αβαβ upon thermal equilibration.

While acetylated porphyrin atropisomers **2** had similar solubility in common organic solvents, the solubility of α_3_β‐**3** proved to be remarkably different from the αβαβ‐**3** conformer. α_3_β‐**3** was found to be soluble in both highly polar solvents (e. g. DMSO, MeOH, CH_3_CN) as well as considerably non‐polar solvents (e. g. hexane, toluene). The amphiphilic behavior could be attributed to the high density of pivalate groups on one side of the superstructure.^[9]^ However, when equally distributed on both faces, for example, αβαβ‐**3**, good solubility was observed only in chlorinated solvents. Hence, for ^1^H NMR comparisons, α_3_β‐**3** and αβαβ‐**3** were dissolved in CDCl_3_, and α_3_β‐**3** and α_3_β‐**2** in *d_6_*‐DMSO (Figure 3). Two main observations can be made: 1) the amide NH groups of α_3_β‐**3** are shifted considerably up‐field as a result of sheltering by the pivalate groups; hence, limiting access for the interactions with solvent molecules (this is also clearly seen in the X‐ray structures); 2) the resolution of α_3_β‐**3** NMR spectra is considerably more defined as a result of the increased structural rigidity.

All of the isolated compounds were screened by UV‐vis spectrophotometric analysis (Figure S24). While the ruffling profiles for the isolated **2** structures were very different (Figure 2), this was not reflected in UV‐vis spectra. Typically, ruffle profiles give rise to the red‐shift of Soret and Q‐band regions.^[20]^ However, all of the structures contain a dominant saddle distortion mode whereas complimentary ruffling (Video S1) has little to no influence on the spectrophotometric changes. Nonplanarity‐induced destabilization of the porphyrin HOMOs is the principal cause of redshifts,^[21]^ hence, a similar magnitude of the overall out‐of‐plane (Δ_oop_) distortion in all structures (Figure S2) results in almost identical absorbance spectra.

On the other hand, stark differences between αβαβ‐**3** and α_3_β‐**3** in the Q‐band region were observed, which resulted in observable colorimetric changes (brown and green, respectively) (Figure 5). In α_3_β‐**3** Q_(0,0)_ has doubled in intensity as to Q_(1,0)_ and batochromically‐shifted by ∼15 nm compared to Q_(0,0)_ of αβαβ‐**3**. The large spectral deviation observed for sterically crowded α_3_β‐**3** may arise from substituent electronic effects.^[22]^ In the α_3_β‐**3** structure, steric repulsion forces one of the phenyl rings to twist to ∼70° relative to the mean plane of the macrocycle, while the others adopt the conventional ∼45° angle (Figure S14). In contrast, neither αβαβ‐**3** nor the acetylated analog α_3_β‐**2** has one dominant phenyl twist, rather all of the rings are orientated at approximately 50°. The relaxation of the high‐symmetry coupling conditions of the orbitals due to the strain‐induced rotation of the single aryl group in α_3_β‐**3** could potentially result in a decreased HOMO‐LUMO gap, red‐shift, and increased intensity.^[23]^ Typically such an effect is observed in unsymmetrical push‐pull porphyrin systems,^[24]^ highlighting the unique characteristic of tension rigidified architecture of α_3_β‐**3**.

**Figure 5:**
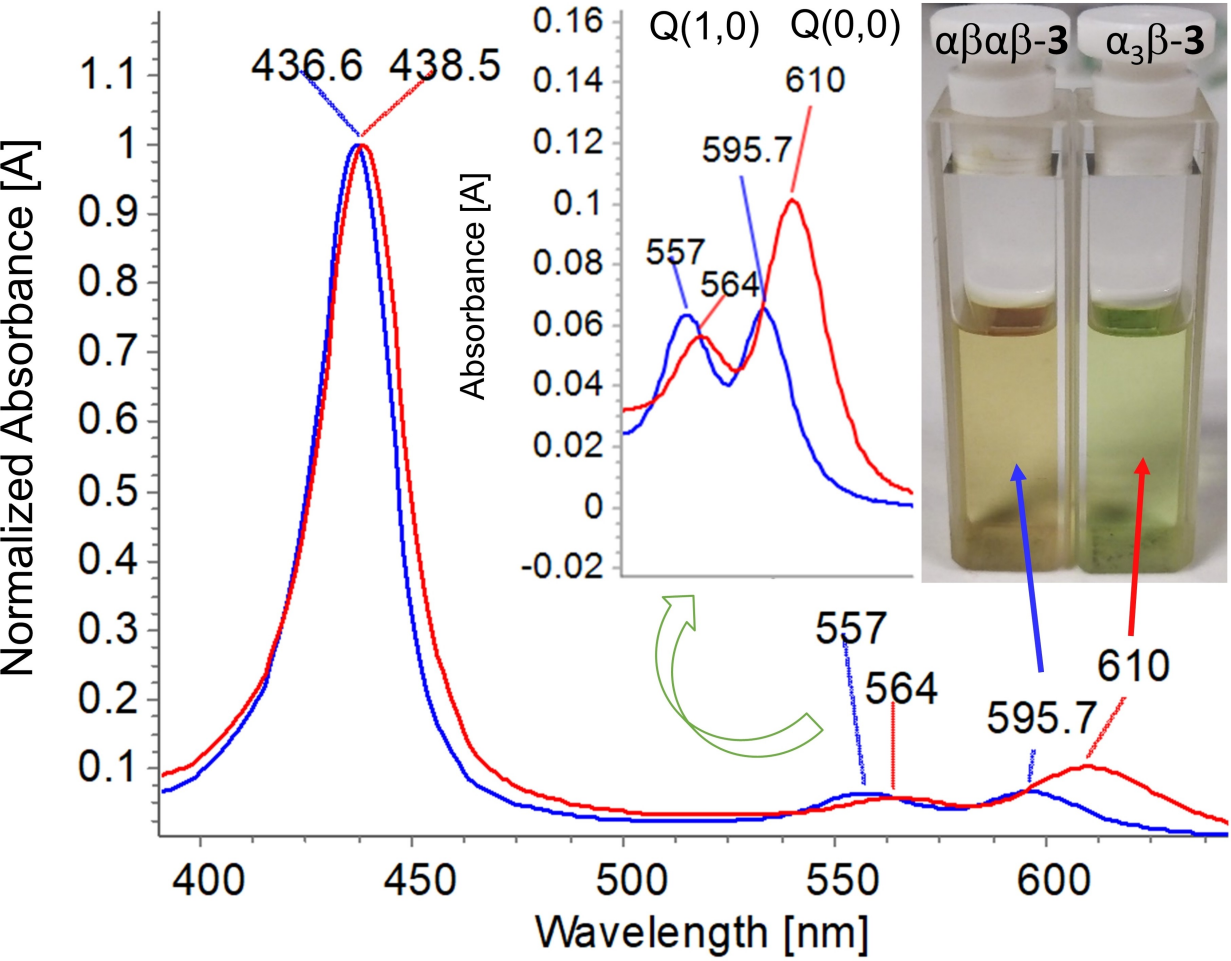
Comparison of the UV‐Vis spectra of α_3_β‐**3** and αβαβ‐**3** with expanded Q‐band region and illustration highlighting the colorimetric differences. Recorded in CHCl_3_.

In conclusion, we have developed a unique atropisomer enrichment strategy that operates by exploiting steric repulsion to induce stereoselective atropisomer interconversion (α_3_β to αβαβ). We show that introducing acetyl groups to the *ortho*‐positions of saddle shaped porphyrin allows thermal enrichment to reach 62 % while introducing bulky pivalamido substituents can drive the selective transformation to over 95 % of the desired atropisomer. Distortion and bulkiness of the periphery appear to be critical factors for enhanced selective atropisomeric enrichment. The formation of sophisticated supramolecular assemblies driven by non‐covalent bonding interactions were found in acetyl porphyrin superstructures. The size of the solvent‐accessible voids, channels, and hydrogen bonding networks were solely dependent on the orientation of the peripheral groups. Upon investigating the photophysical properties, marginal absorbance spectral differences between acetylated porphyrin atropisomers were found. Unlike acetyl groups, the pivalamido‐based α_3_β atropisomer attained a significant bathochromic shift of the Q‐band region in comparison to the αβαβ conformation. Upon detailed X‐ray crystallographic analysis, the difference in absorbance spectra was rationalized in terms of phenyl rotation induced by the severe steric repulsion, which caused geometrical and stereoelectronic desymmetrization. The combined effects of a nonplanar porphyrin scaffold with highly strained “picket‐fence” components lay the foundation for supramolecular atropisomeric architectures which could potentially result in promising superstructures for sophisticated receptor systems.^[25]^

Deposition Numbers 2118018 (for α_4_‐**2**), 2118019 for (α_3_β‐**3**), 2118020 (for α_3_β‐**2**), 2118021 (for α_2_β_2_‐**2**), 2118023 (for α,β,α,β‐**2**), 2118022 (for α,β,α,β‐**3**) contain the supplementary crystallographic data for this paper. These data are provided free of charge by the joint Cambridge Crystallographic Data Centre and Fachinformationszentrum Karlsruhe Access Structures service.

## Conflict of interest

The authors declare no conflict of interest.

## Supporting information

As a service to our authors and readers, this journal provides supporting information supplied by the authors. Such materials are peer reviewed and may be re‐organized for online delivery, but are not copy‐edited or typeset. Technical support issues arising from supporting information (other than missing files) should be addressed to the authors.

Supporting Information

## Data Availability

The data that support the findings of this study are available in the supplementary material of this article.
