# Supplementary material for: Steric Repulsion Induced Conformational Switch in Supramolecular Structures
Source: Chemistry. 2021 Dec 2;28(4):e202103879. doi: 10.1002/chem.202103879 (PMC9299809; doi:10.1002/chem.202103879)
Supplement: Supplementary file 1 — Supporting Information [file CHEM-28-0-s001.pdf]

# Chemistry–A European Journal

Supporting Information


## Steric Repulsion Induced Conformational Switch in Supramolecular Structures


Karolis Norvaiša, Sophie Maguire, Claire Donohoe, John E. O'Brien, Brendan Twamley, Ligia C. Gomes-da-Silva, and Mathias O. Senge*

Supporting Information

**Table of Contents**

Results and Discussion ........ 3
Synthetic Scheme of Target Compounds ........ 3
Structural Determination of Isolated Structures ........ 4
Structural analysis ........ 7
$^1$H NMR analysis of isolated compounds ........ 16
HPLC analysis ........ 19
Monitoring $\alpha_3\beta$-**3** reaction ........ 21
UV-vis spectrophotometry ........ 22
Experimental Procedures ........ 23
General Materials and Methods ........ 23
Synthesis and Characterization of Compounds ........ 24
*Synthesis and characterization of $\alpha_3\beta$-**2*** ........ *24*
*Synthesis and characterization of αβαβ-**2*** ........ *30*
*Synthesis and characterization of $\alpha_2\beta_2$-**2*** ........ *36*
*Synthesis and characterization of $\alpha_4$-**2*** ........ *42*
*Synthesis and characterization of αβαβ-**3*** ........ *47*
*Synthesis and characterization of $\alpha_3\beta$-**3*** ........ *52*
References ........ 60

**Results and Discussion**

**Synthetic Scheme of Target Compounds**

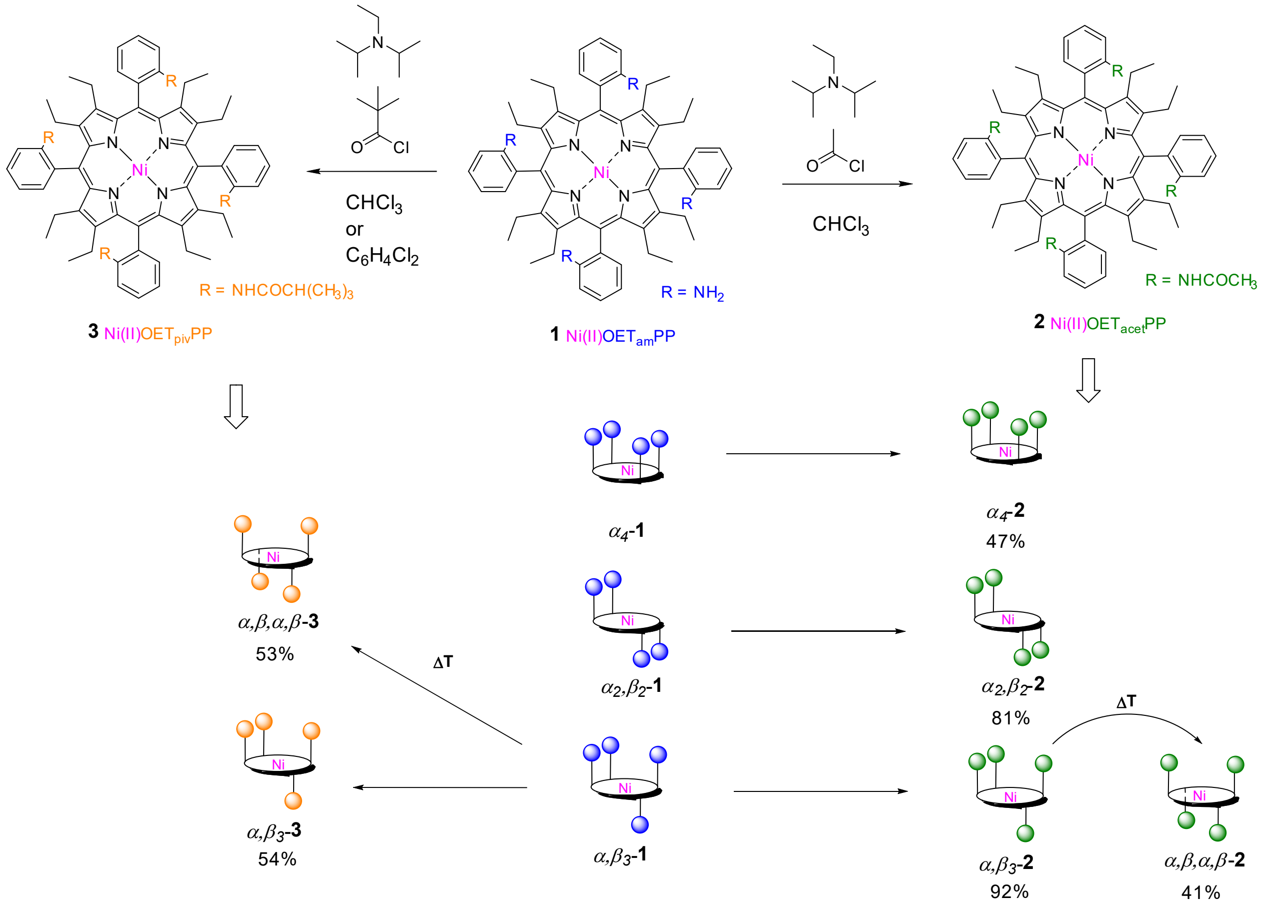


**Scheme S1.** Synthetic scheme for the preparation of the target compounds; ΔT represents atropisomeric thermal interconversion.

**Structural Determination of Isolated Structures**

Crystals were grown following the protocol developed by Hope, liquid-liquid diffusion in $CHCl_3$ and methanol or oversaturated solutions in DMSO or Hexane.[1] Using Olex2, the structure was solved with the XT structure solution program, using the intrinsic phasing solution method and refined against $|F^2|$ with XL using least squares minimization.[2] The C and N bound H atoms were placed in their expected calculated positions and refined as riding model: N–H = 0.88 Å, C–H = 0.95–0.98 Å, with $U_{iso}$ (H) = 1.5$U_{eq}$ (C) for methyl H atoms and 1.2$U_{eq}$ (C, N) for all other atoms H atoms. Details of data refinements can be found in Table S1. All images were prepared using Olex2.[2a]

In the structure of *α₃β*-**2** one of the acetamide units was modelled over two positions 62%:38%; the solvent accessible void contains chloroform, acetonitrile and dichloromethane molecules that were modelled with rigid fragments from the fragment database with corresponding SADI, SIMU, ISOR and DFIX commands.

In the structure of $\alpha_2\beta_2$-**2** one of the ethyl units (at C18) was modelled over two positions 89%:11%; one of the solvent accessible voids contains disordered acetonitrile molecules over two positions 50%:50% that were modelled with rigid fragments from the fragment database with corresponding SIMU and ISOR commands.

In the structure *α,β,α,β*-**2**, the solvent accessible channel contains disordered hexane and two chloroform molecules modelled over three positions 37%:10%:3% that were modelled with rigid fragments from the fragment database with corresponding SIMU and ISOR commands.

In the structure of $\alpha_4$-**2**, one of the acetyl groups (at C5) modelled over two positions 68%:32% using SIMU restraints. Acetonitrile was modelled as rigid unit from the fragment data base with additional SIMU and ISOR restraints.

In the structure of $\alpha_3\beta$-**3**, one of the pivaloylamide groups is disordered at C52 over two positions (66%:37%, also one of the phenyl rings has been modelled over two positions (41%:59%) at C10 position using ISOR and SIMU restraints. A highly disordered diethyl partially occupied ether molecule was modelled over two positions 27%:23% using DANG, DFIX, SIMU and ISOR restraints.

In the structure *α,β,α,β*-**3**, one of the pivaloylamide groups is disordered at C5B over two positions 32%:68%. PLATON SQUEEZE[3] was used to remove highly disordered solvent molecules: Solvent Accessible Volume = 289 Angstroms. Electrons Found in S.A.V. = 43. 2 voids per cell - ca 20 electrons each, approx. 50% $CH_2Cl_2$ per void.

Deposition numbers 2118018-2118023 contain the supplementary crystallographic data for this paper. These data are provided free of charge by the joint Cambridge Crystallographic Data Centre and Fachinformationszentrum Karlsruhe http://www.ccdc.cam.ac.uk/structures.

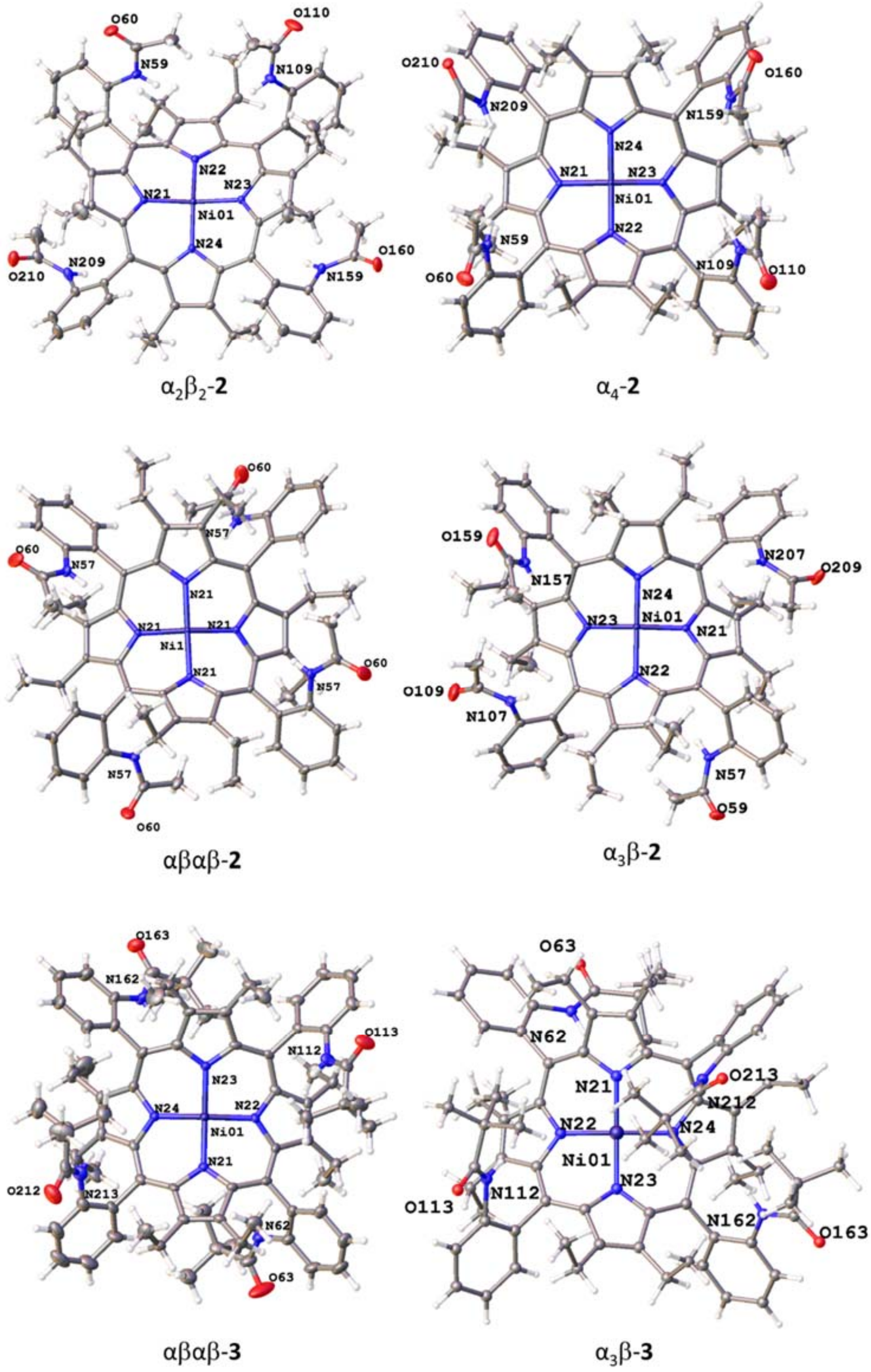


**Figure S1.** Molecular structures with labeled hetero atoms of isolated and analyzed compounds from X-ray crystallography. Solvent molecules omitted for clarity; thermal displacement shown at 50% probability.

**Table S1:** Details of XRD data refinement of *α₃β-**2***, *α₂β₂-**2***, *α,β,α,β-**2***, *α₄-**2***, *α₃β-**3***, *α,β,α,β-**3***

| ***Compound*** | $\alpha_3\beta$-**2** | $\alpha_2\beta_2$-**2** | α,β,α,β-**2** | $\alpha_4$-**2** | $\alpha_3\beta$-**3** | α,β,α,β-**3** |
|---|---|---|---|---|---|---|
| *Internal code* | TCD1744 | TCD1767 | TCD1773 | TCD1769 | TCD1821 | TCD1780 |
| *CCDC #* | 2118020 | 2118021 | 2118023 | 2118018 | 2118019 | 2118022 |
| *Empirical formula* | $C_{75.01}H_{82.33}Cl_{2.17}N_{11.11}NiO_4$ | $C_{72}H_{78}N_{10}NiO_4$ | $C_{77.42}H_{93.29}Cl_{1.55}N_8NiO_4$ | $C_{70}H_{79}N_9NiO_6$ | $C_{88}H_{115}N_8NiO_{4.5}$ | $C_{80}H_{96}N_8NiO_4$ |
| *Formula weight* | 1339.25 | 1206.15 | 1313.58 | 1201.13 | 1415.58 | 1292.35 |
| *Temperature/K* | 100(2) | 100(2) | 100(2) | 100(2) | 100(2) | 100(2) |
| *Crystal system* | Triclinic | Monoclinic | Tetragonal | Triclinic | Triclinic | Triclinic |
| *Space group* | $P\bar{1}$ | $P2_1/c$ | $P4_2/n$ | $P\bar{1}$ | $P\bar{1}$ | $P\bar{1}$ |
| *a/Å* | 12.3146(6) | 14.1193(5) | 14.6384(4) | 12.8178(5) | 14.1558(6) | 15.8514(6) |
| *b/Å* | 12.9854(6) | 15.9066(6) | 14.6384(4) | 14.2126(6) | 15.1923(6) | 16.9846(6) |
| *c/Å* | 23.4365(11) | 28.4015(10) | 16.1568(7) | 19.3079(8) | 20.5683(8) | 28.2326(10) |
| *α/°* | 98.966(2) | 90 | 90 | 93.618(2) | 87.2160(10) | 102.4420(10) |
| *β/°* | 103.058(2) | 92.3630(10) | 90 | 108.1670(10) | 84.9610(10) | 97.8380(10) |
| *γ/°* | 105.352(2) | 90 | 90 | 113.7660(10) | 64.2030(10) | 100.1910(10) |
| *Volume/Å³* | 3426.5(3) | 6373.3(4) | 3462.1(2) | 2985.7(2) | 3966.9(3) | 7184.0(5) |
| *Z* | 2 | 4 | 2 | 2 | 2 | 4 |
| $D_{calc}$ *g/cm³* | 1.298 | 1.257 | 1.260 | 1.336 | 1.185 | 1.195 |
| *μ/mm⁻¹* | 0.426 | 0.362 | 0.396 | 0.388 | 0.300 | 0.325 |
| *F(000)* | 1414.0 | 2560.0 | 1400.0 | 1276.0 | 1526.0 | 2768 |
| *Crystal size/mm³* | 0.43 × 0.37 × 0.18 | 0.5 × 0.32 × 0.17 | 0.431 × 0.4 × 0.322 | 0.34 × 0.19 × 0.09 | 0.395 × 0.231 × 0.159 | 0.24 x 0.21 x 0.08 |
| *Radiation* | MoKα | MoKα | MoKα | MoKα | MoKα | MoKα |
| *Wavelength/Å* | 0.71073 | 0.71073 | 0.71073 | 0.71073 | 0.71073 | 0.71073 |
| *2θ/°* | 5.5 to 55.966 | 4.738 to 56.852 | 5.566 to 56.742 | 4.702 to 56.09 | 5.236 to 56.084 | 2.158 to 26.890 |
| *Reflections collected* | 92862 | 151759 | 77267 | 69157 | 76391 | 149737 |
| *Independent reflections* | 16470 | 15977 | 4337 | 14446 | 19137 | 30900 |
| $R_{int}$ | 0.0360 | 0.0525 | 0.0309 | 0.0688 | 0.0454 | 0.1737 |
| $R_{sigma}$ | 0.0255 | 0.0268 | 0.0105 | 0.0519 | 0.0412 | 0.1377 |
| *Restraints* | 424 | 146 | 281 | 109 | 445 | 274 |
| *Parameters* | 1020 | 841 | 309 | 837 | 1108 | 1821 |
| *GooF* | 1.038 | 1.042 | 1.037 | 1.025 | 1.019 | 1.008 |
| $R_1$ *[I> 2σ (I)]* | 0.0444 | 0.0410 | 0.0469 | 0.0457 | 0.0459 | 0.0732 |
| $wR_2$ *[I> 2σ (I)]* | 0.1081 | 0.0935 | 0.1282 | 0.0923 | 0.1033 | 0.1378 |
| $R_1$ *[all data]* | 0.0561 | 0.0560 | 0.0521 | 0.0737 | 0.0687 | 0.1691 |
| $wR_2$ *[all data]* | 0.1160 | 0.1019 | 0.1336 | 0.1041 | 0.1160 | 0.1761 |
| *Largest peak/e Å⁻³* | 0.73 | 0.39 | 1.06 | 0.63 | 0.93 | 0.58 |
| *Deepest hole/e Å⁻³* | -0.64 | -0.30 | -0.63 | -0.58 | -0.52 | -0.32 |

**Structural analysis**

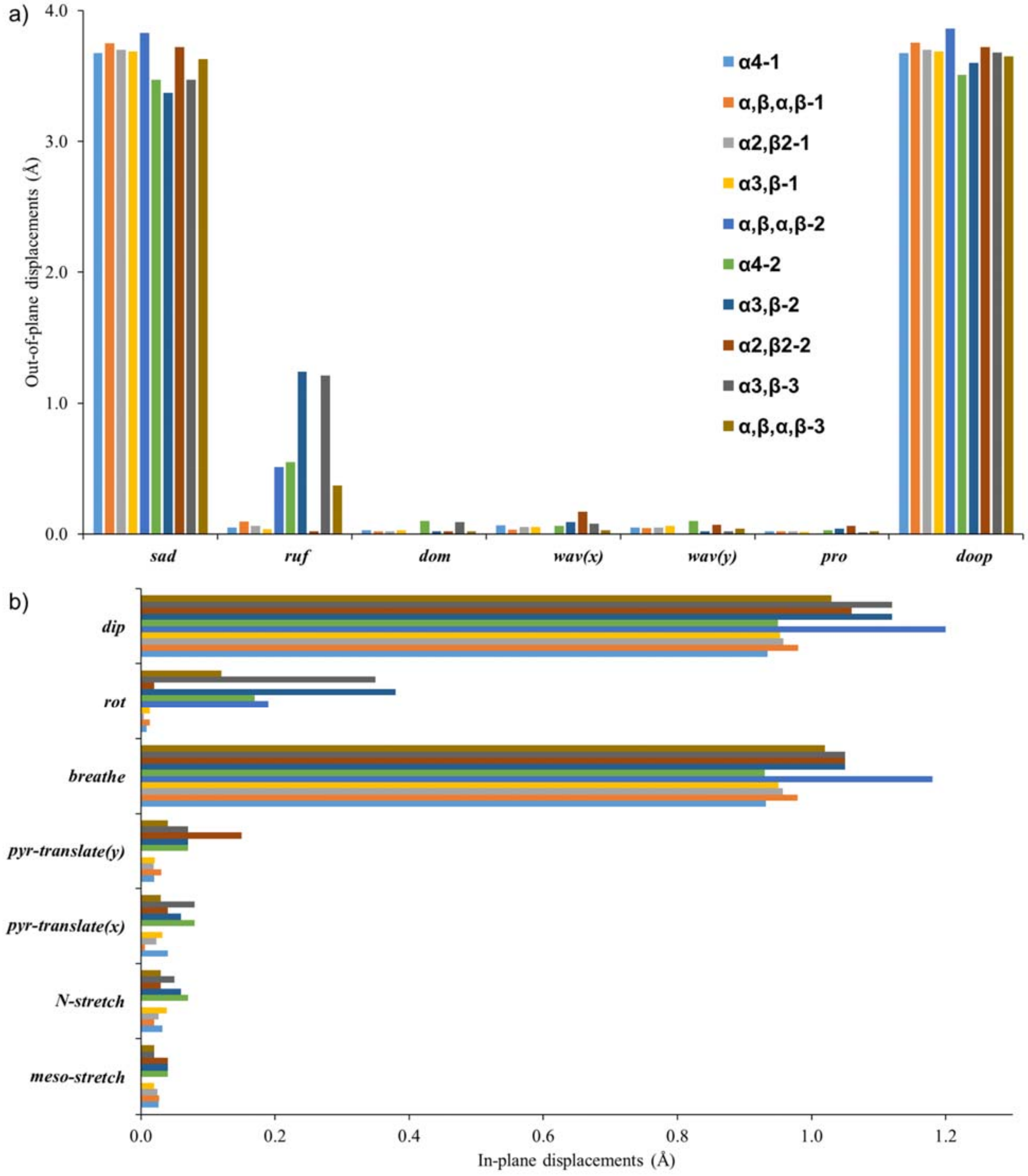


**Figure S2a.** Illustration of the a) out-of-plane (*sad* ($B_{2u}$), *ruf* ($B_{1u}$), *dom* ($A_{2u}$), *wav*(*x*) ($E_g(x)$), *wav*(*y*), ($E_g(y)$) and *pro* ($A_{1u}$)) and b) in-plane (*m-str* ($B_{2g}$), *N-str* ($B_{1g}$), *bre* ($A_{1g}$), *trans*(*x*) $E_u(x)$, *trans*(*y*) $E_u(y)$ and *rot* ($A_{2g}$)) normal-coordinate structural decomposition results for **1**, **2** and **3** atropisomers.

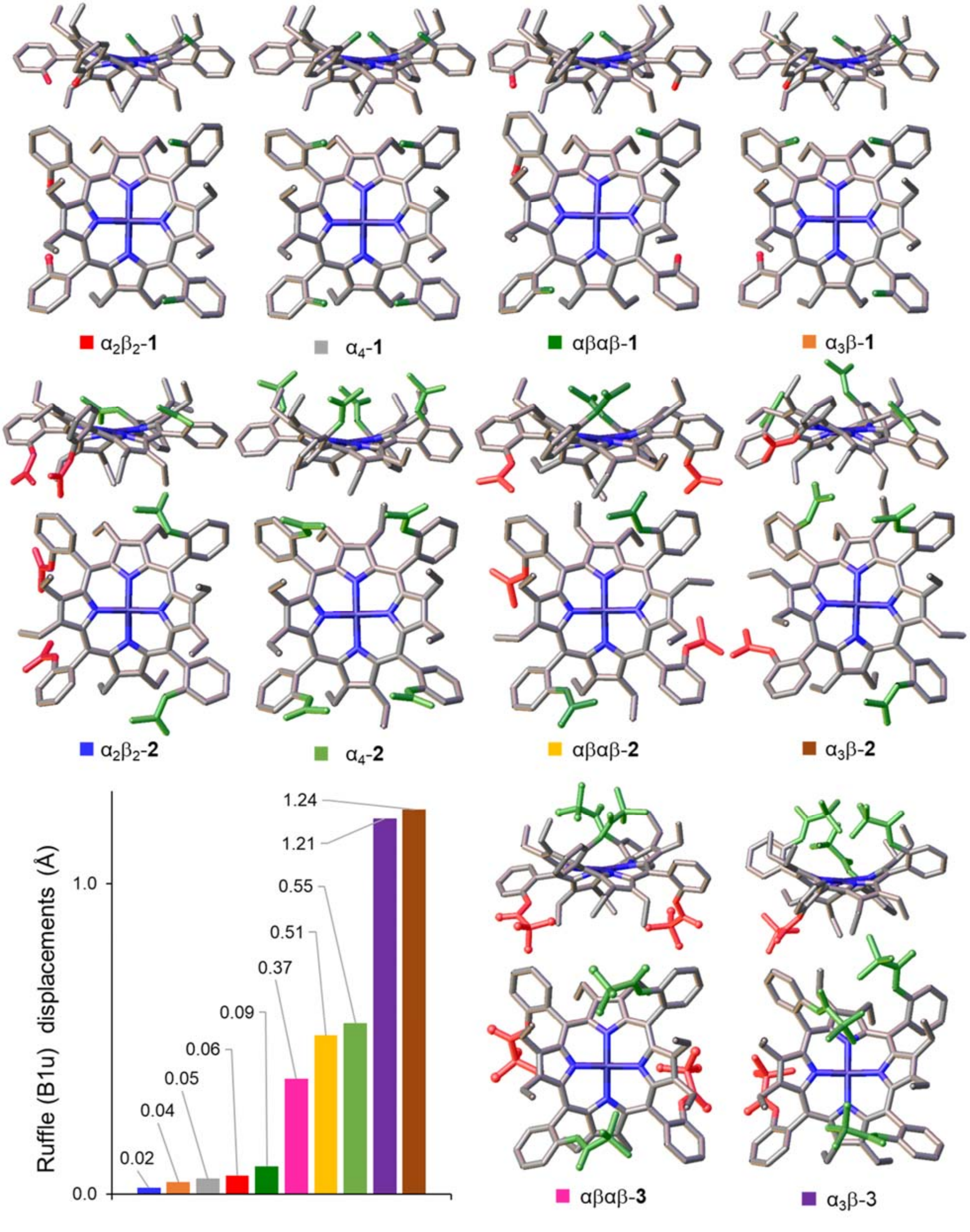


**Figure S2b.** Molecular structures of atropisomers discussed in this study, in green – peripheral groups (amine (**1**) acetyl (**2**) or pivaloyl (**3**)) above the macrocycle plane, in red – below the plane. Hydrogen atoms and solvent molecules were omitted for clarity. Bottom left corner, a chart of the ruffle distortion obtained from out-of-plane normal-coordinate structural decomposition (NSD) (see Figure S2a for full NSD analysis).

| basis | $\Delta_{oop}$ | $\delta_{oop}$ | $B_{2u}$ | $B_{1u}$ | $A_{2u}$ | $E_g(x)$ | $E_g(y)$ | $A_{1u}$ |
|---|---|---|---|---|---|---|---|---|
| min. | 3.72 | 0.01 | -3.72 | -0.02 | -0.02 | 0.15 | -0.06 | -0.06 |
| ext. | 3.72 | 0.00 | -3.72 | -0.02 | -0.02 | 0.15 | -0.06 | -0.06 |
| | | | 0.10 | 0.00 | 0.00 | -0.07 | 0.02 | 0.01 |
| total | 3.72 | 0.00 | -3.72 | -0.02 | -0.02 | 0.15 | -0.06 | -0.06 |
| | | | 0.11 | 0.00 | 0.00 | -0.06 | 0.02 | 0.01 |
| | | | 0.06 | -0.01 | -0.01 | 0.02 | -0.03 | |
| | | | | | | -0.01 | 0.02 | |
| | | | | | | 0.03 | 0.00 | |
| comp. | 3.72 | 0.00 | 3.72 | 0.02 | 0.02 | 0.17 | 0.07 | 0.06 |

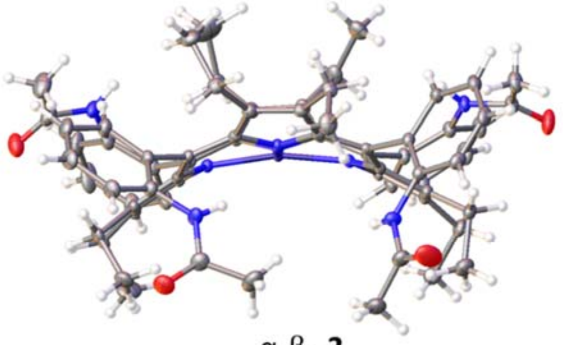

$\alpha_2\beta_2$-**2**

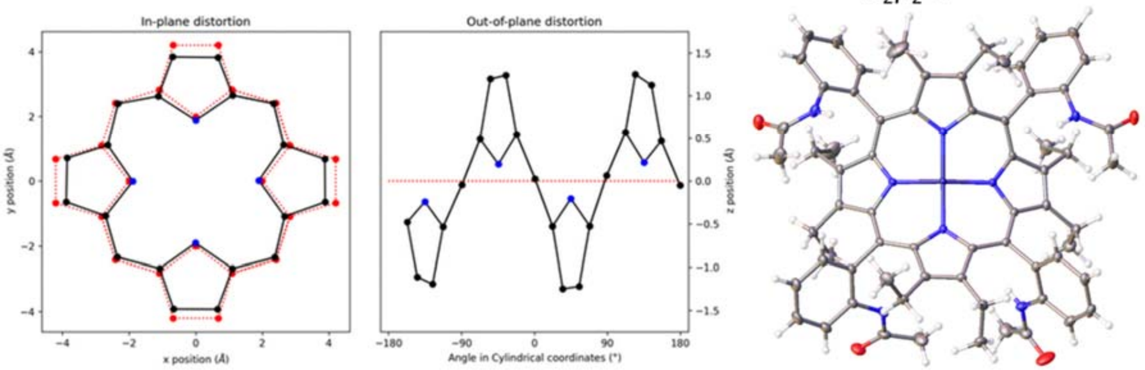


**Figure S3.** Top: out-of-plane NSD results of *α₂β₂-**2***; bottom: out-of-plane and in-plane skeletal plots of the porphyrin *α₂β₂-**2*** core. Porphyrin *α₂β₂-**2*** is represented in black(C) and blue(N), with the reference structure (CuTPP) in red dotted lines.[4] Solvent molecules omitted for clarity, thermal ellipsoids shown at 50% probability.

| basis | $\Delta_{oop}$ | $\delta_{oop}$ | $B_{2u}$ | $B_{1u}$ | $A_{2u}$ | $E_g(x)$ | $E_g(y)$ | $A_{1u}$ |
|---|---|---|---|---|---|---|---|---|
| min. | 3.51 | 0.00 | -3.47 | 0.54 | 0.09 | 0.03 | 0.08 | 0.03 |
| ext. | 3.51 | 0.00 | -3.47 | 0.54 | 0.09 | 0.03 | 0.08 | 0.03 |
| | | | 0.09 | 0.05 | -0.04 | 0.00 | -0.01 | 0.00 |
| total | 3.51 | 0.00 | -3.47 | 0.54 | 0.09 | 0.03 | 0.08 | 0.03 |
| | | | 0.09 | 0.05 | -0.05 | 0.00 | 0.00 | 0.00 |
| | | | 0.01 | 0.02 | -0.01 | 0.04 | 0.03 | |
| | | | | | | -0.03 | 0.00 | |
| | | | | | | 0.00 | 0.04 | |
| comp. | 3.51 | 0.00 | 3.47 | 0.55 | 0.10 | 0.06 | 0.10 | 0.03 |

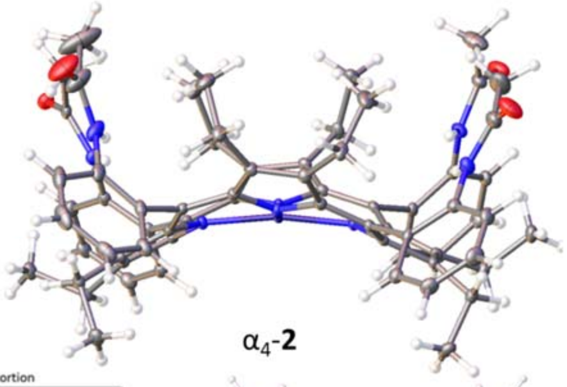

$\alpha_4$-**2**

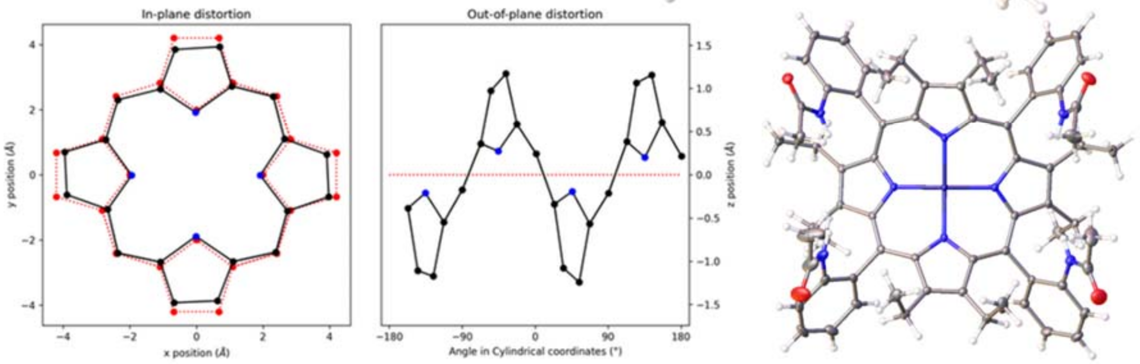


**Figure S4.** Top: out-of-plane NSD results of *α₄-**2***; bottom: out-of-plane and in-plane skeletal plots of the porphyrin *α₄-**2*** core. Porphyrin *α₄-**2*** is represented in black(C) and blue(N), with the reference structure (CuTPP) in red dotted lines.[4] Solvent molecules omitted for clarity, thermal ellipsoids shown at 50% probability.

| basis | $\Delta_{oop}$ | $\delta_{oop}$ | $B_{2u}$ | $B_{1u}$ | $A_{2u}$ | $E_g(x)$ | $E_g(y)$ | $A_{1u}$ |
|---|---|---|---|---|---|---|---|---|
| min. | 3.86 | 0.00 | -3.83 | -0.51 | 0.00 | 0.00 | 0.00 | 0.00 |
| ext. | 3.86 | 0.00 | -3.83 | -0.51 | 0.00 | 0.00 | 0.00 | 0.00 |
| | | | 0.13 | 0.00 | 0.00 | 0.00 | 0.00 | 0.00 |
| total | 3.86 | 0.00 | -3.83 | -0.51 | 0.00 | 0.00 | 0.00 | 0.00 |
| | | | 0.13 | 0.00 | 0.00 | 0.00 | 0.00 | 0.00 |
| | | | 0.06 | 0.00 | 0.00 | 0.00 | 0.00 | |
| | | | | | | 0.00 | 0.00 | |
| | | | | | | 0.00 | 0.00 | |
| comp. | 3.86 | 0.00 | 3.83 | 0.51 | 0.00 | 0.00 | 0.00 | 0.00 |

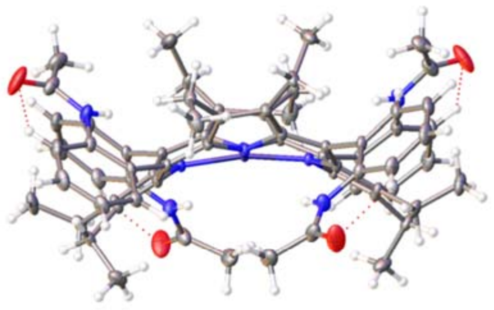

αβαβ-**2**

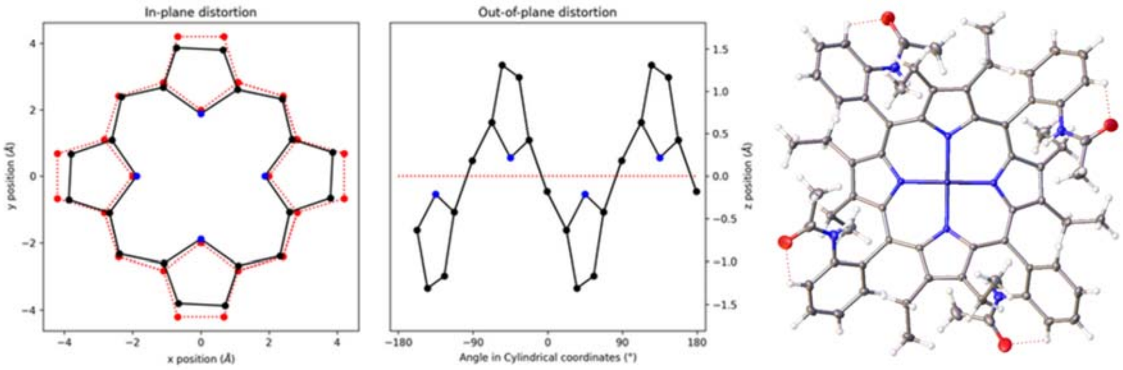


**Figure S5.** Top: out-of-plane NSD results of *α,β,α,β-**2***; bottom: out-of-plane and in-plane skeletal plots of the porphyrin *α,β,α,β-**2*** core. Porphyrin *α,β,α,β-**2*** is represented in black (C) and blue (N), with the reference structure (CuTPP) in red dotted lines.[4] Solvent molecules omitted for clarity, thermal ellipsoids shown at 50% probability.

| basis | $\Delta_{oop}$ | $\delta_{oop}$ | $B_{2u}$ | $B_{1u}$ | $A_{2u}$ | $E_g(x)$ | $E_g(y)$ | $A_{1u}$ |
|---|---|---|---|---|---|---|---|---|
| min. | 3.65 | 0.00 | -3.63 | -0.37 | -0.01 | 0.02 | -0.04 | -0.02 |
| ext. | 3.65 | 0.00 | -3.63 | -0.37 | -0.02 | 0.02 | -0.04 | -0.02 |
| | | | 0.11 | 0.01 | -0.02 | -0.02 | 0.01 | 0.01 |
| total | 3.65 | 0.00 | -3.63 | -0.37 | -0.02 | 0.02 | -0.04 | -0.02 |
| | | | 0.12 | 0.01 | -0.02 | -0.02 | 0.01 | 0.01 |
| | | | 0.04 | 0.00 | -0.01 | 0.00 | -0.02 | |
| | | | | | | 0.00 | 0.01 | |
| | | | | | | 0.00 | 0.00 | |
| comp. | 3.65 | 0.00 | 3.63 | 0.37 | 0.02 | 0.03 | 0.04 | 0.02 |

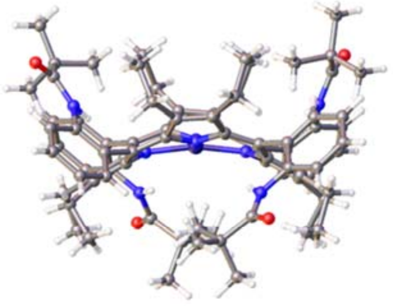

αβαβ-**3**

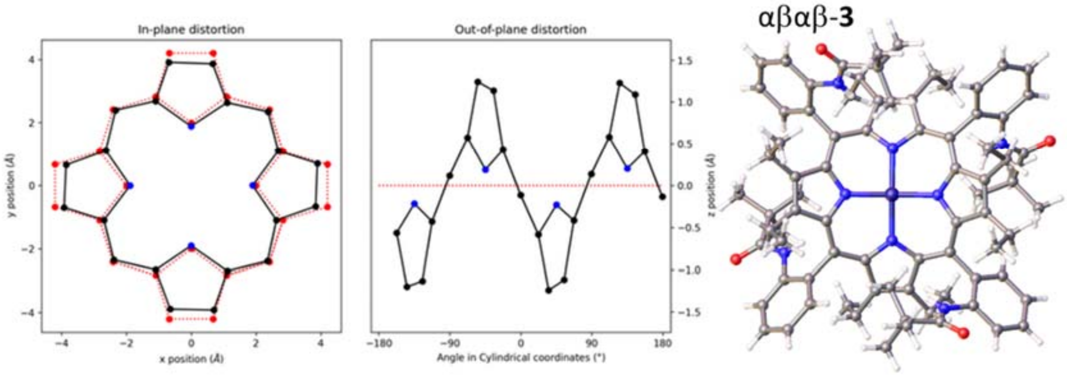


**Figure S6.** Top: out-of-plane NSD results of *α,β,α,β-**3***; bottom: out-of-plane and in-plane skeletal plots of the porphyrin *α,β,α,β-**3*** core. Porphyrin *α,β,α,β-**3*** is represented in black (C) and blue (N), with the reference structure (CuTPP) in red dotted lines.[4] Solvent molecules omitted for clarity, thermal ellipsoids shown at 50% probability.

| basis | $\Delta_{oop}$ | $\delta_{oop}$ | $B_{2u}$ | $B_{1u}$ | $A_{2u}$ | $E_g(x)$ | $E_g(y)$ | $A_{1u}$ |
|---|---|---|---|---|---|---|---|---|
| min. | 3.60 | 0.01 | -3.37 | -1.24 | 0.02 | 0.08 | 0.00 | 0.03 |
| ext. | 3.60 | 0.00 | -3.37 | -1.24 | 0.02 | 0.07 | 0.00 | 0.03 |
| | | | 0.14 | 0.01 | -0.01 | -0.04 | 0.00 | -0.02 |
| total | 3.60 | 0.00 | -3.37 | -1.24 | 0.02 | 0.07 | 0.00 | 0.03 |
| | | | 0.14 | 0.01 | -0.01 | -0.04 | 0.00 | -0.02 |
| | | | 0.04 | -0.05 | -0.01 | 0.03 | -0.02 | |
| | | | | | | -0.02 | 0.00 | |
| | | | | | | 0.01 | 0.01 | |
| comp. | 3.60 | 0.00 | 3.37 | 1.24 | 0.02 | 0.09 | 0.02 | 0.04 |

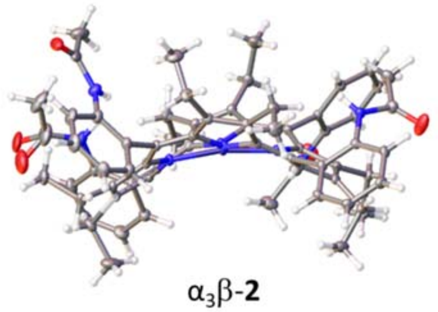

$\alpha_3\beta$-**2**

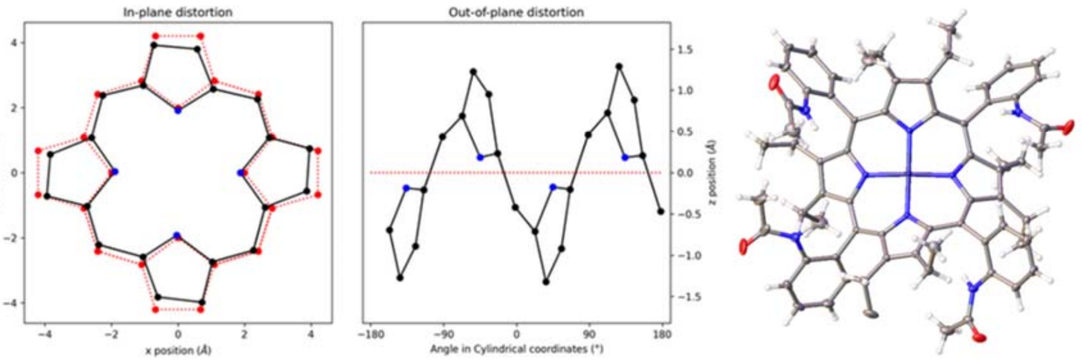


**Figure S7.** Top: out-of-plane NSD results of *α₃β-2*; bottom: out-of-plane and in-plane skeletal plots of the porphyrin *α₃β-2* core. Porphyrin *α₃β-2* is represented in black (C) and blue (N), with the reference structure (CuTPP) in red dotted lines.[4] Solvent molecules omitted for clarity, thermal ellipsoids shown at 50% probability.

| basis | $\Delta_{oop}$ | $\delta_{oop}$ | $B_{2u}$ | $B_{1u}$ | $A_{2u}$ | $E_g(x)$ | $E_g(y)$ | $A_{1u}$ |
|---|---|---|---|---|---|---|---|---|
| min. | 3.68 | 0.01 | 3.47 | 1.21 | 0.09 | 0.08 | 0.02 | 0.01 |
| ext. | 3.68 | 0.00 | 3.47 | 1.21 | 0.08 | 0.08 | 0.02 | 0.01 |
| | | | -0.13 | -0.03 | -0.05 | 0.02 | 0.00 | -0.03 |
| total | 3.68 | 0.00 | 3.47 | 1.21 | 0.09 | 0.09 | 0.02 | 0.01 |
| | | | -0.13 | -0.03 | -0.05 | 0.02 | 0.00 | -0.03 |
| | | | -0.05 | 0.05 | 0.01 | -0.01 | -0.03 | |
| | | | | | | 0.01 | 0.00 | |
| | | | | | | 0.01 | 0.00 | |
| comp. | 3.68 | 0.00 | 3.47 | 1.22 | 0.10 | 0.09 | 0.04 | 0.03 |

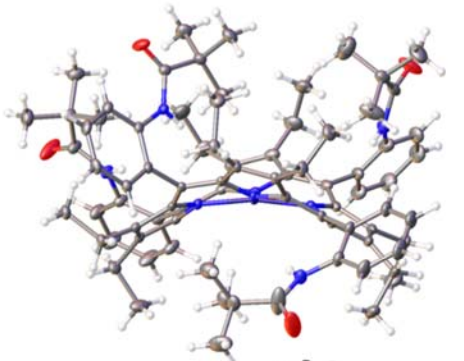

$\alpha_3\beta$-**3**

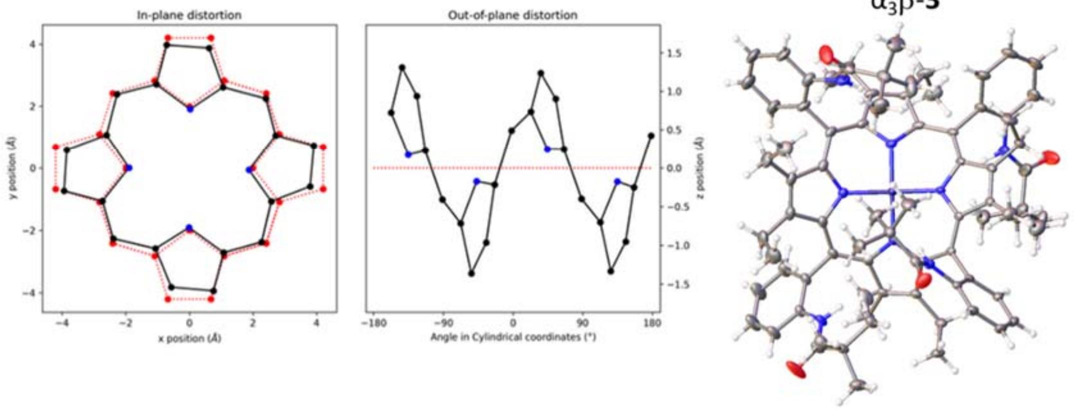


**Figure S8.** Top: out-of-plane NSD results of *α₃β-3*; bottom: out-of-plane and in-plane skeletal plots of the porphyrin *α₃β-3* core. Porphyrin *α₃β-3* is represented in black (C) and blue (N), with the reference structure (CuTPP) in red dotted lines.[4] Solvent molecules omitted for clarity, thermal ellipsoids shown at 50% probability.

| basis | $\Delta_{oop}$ | $\delta_{oop}$ | $B_{2u}$ | $B_{1u}$ | $A_{2u}$ | $E_g(x)$ | $E_g(y)$ | $A_{1u}$ |
|---|---|---|---|---|---|---|---|---|
| min. | 3.43 | 0.01 | -3.23 | 1.12 | 0.12 | 0.21 | -0.05 | -0.02 |
| ext. | 3.43 | 0.00 | -3.22 | 1.12 | 0.12 | 0.21 | -0.05 | -0.02 |
| | | | 0.15 | -0.02 | 0.02 | -0.06 | 0.00 | 0.00 |
| total | 3.43 | 0.00 | -3.22 | 1.12 | 0.12 | 0.21 | -0.05 | -0.02 |
| | | | 0.15 | -0.02 | 0.02 | -0.06 | 0.00 | 0.00 |
| | | | 0.04 | 0.02 | 0.00 | 0.01 | -0.01 | |
| | | | | | | -0.01 | 0.00 | |
| | | | | | | 0.01 | 0.00 | |
| comp. | 3.43 | 0.00 | 3.23 | 1.12 | 0.12 | 0.22 | 0.05 | 0.02 |

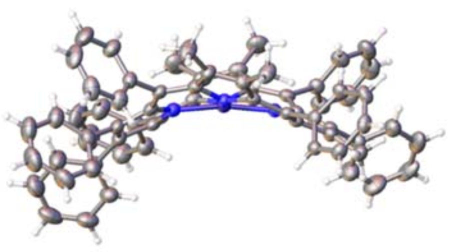

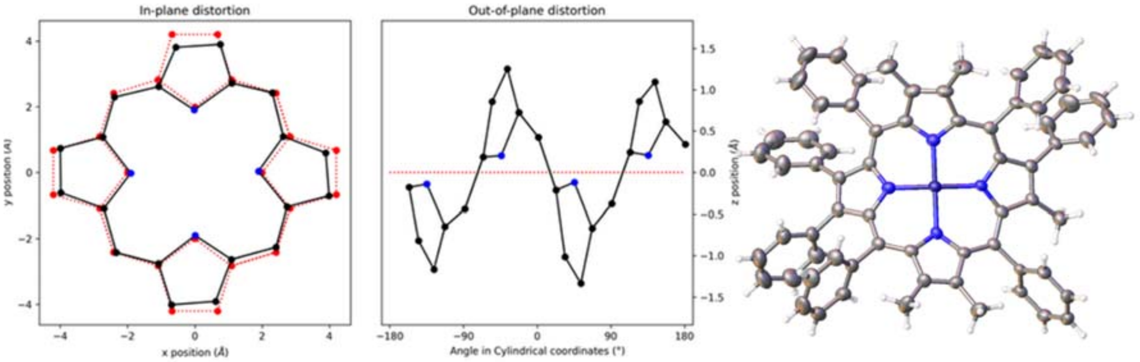


**Figure S9.** Top: out-of-plane NSD results of unsymmetrical dodecasubstituted porphyrin;[5] bottom: out-of-plane and in-plane skeletal plots of the porphyrin[5] core. Porphyrin[5] is represented in black (C) and blue (N), with the reference structure (CuTPP) in red dotted lines.[4] Solvent molecules omitted for clarity, thermal ellipsoids shown at 50% probability.

| basis | $\Delta_{oop}$ | $\delta_{oop}$ | $B_{2u}$ | $B_{1u}$ | $A_{2u}$ | $E_g(x)$ | $E_g(y)$ | $A_{1u}$ |
|---|---|---|---|---|---|---|---|---|
| min. | 3.96 | 0.13 | -3.78 | 1.18 | -0.06 | 0.07 | -0.03 | 0.00 |
| ext. | 4.01 | 0.00 | -3.77 | 1.18 | -0.06 | 0.07 | -0.03 | 0.00 |
| | | | 0.71 | 0.08 | 0.01 | -0.02 | 0.02 | 0.00 |
| total | 4.02 | 0.00 | -3.77 | 1.18 | -0.06 | 0.07 | -0.03 | 0.00 |
| | | | 0.72 | 0.08 | 0.01 | -0.02 | 0.02 | 0.00 |
| | | | 0.08 | 0.04 | -0.01 | 0.00 | 0.01 | |
| | | | | | | 0.00 | -0.01 | |
| | | | | | | 0.01 | -0.01 | |
| comp. | 4.02 | 0.00 | 3.84 | 1.18 | 0.06 | 0.07 | 0.04 | 0.01 |

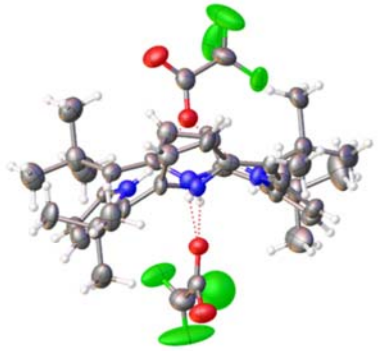

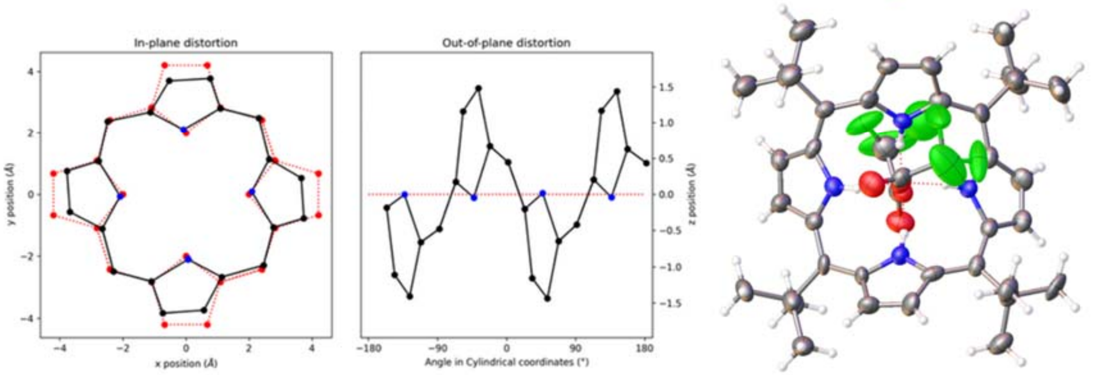


**Figure S10.** Top: out-of-plane NSD results of $[H_4TtBP][CF_3COO^-]_2$•$2CF_3COOH$;[6] bottom: out-of-plane and in-plane skeletal plots of the porphyrin[6] core. Porphyrin[6] is represented in black (C) and blue (N), with the reference structure (CuTPP) in red dotted lines.[4] Solvent molecules omitted for clarity, thermal ellipsoids shown at 50% probability.

| basis | $\Delta_{oop}$ | $\delta_{oop}$ | $B_{2u}$ | $B_{1u}$ | $A_{2u}$ | $E_g(x)$ | $E_g(y)$ | $A_{1u}$ |
|---|---|---|---|---|---|---|---|---|
| min. | 3.35 | 0.10 | 3.27 | 0.73 | 0.00 | -0.06 | 0.13 | 0.12 |
| ext. | 3.40 | 0.00 | 3.26 | 0.73 | 0.00 | -0.06 | 0.13 | 0.12 |
| | | | -0.60 | 0.06 | 0.10 | -0.09 | 0.02 | -0.13 |
| total | 3.40 | 0.00 | 3.26 | 0.73 | 0.00 | -0.06 | 0.13 | 0.12 |
| | | | -0.60 | 0.06 | 0.10 | -0.10 | 0.02 | -0.13 |
| | | | -0.01 | 0.06 | 0.03 | -0.02 | 0.07 | |
| | | | | | | -0.03 | -0.04 | |
| | | | | | | -0.01 | -0.05 | |
| comp. | 3.40 | 0.00 | 3.31 | 0.73 | 0.10 | 0.12 | 0.16 | 0.18 |

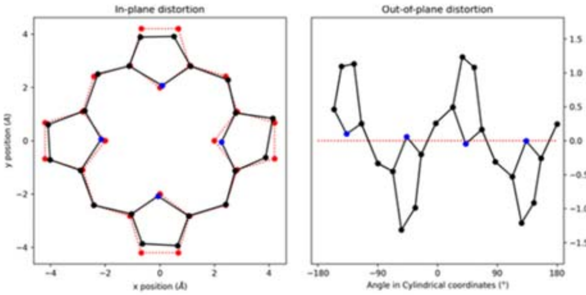


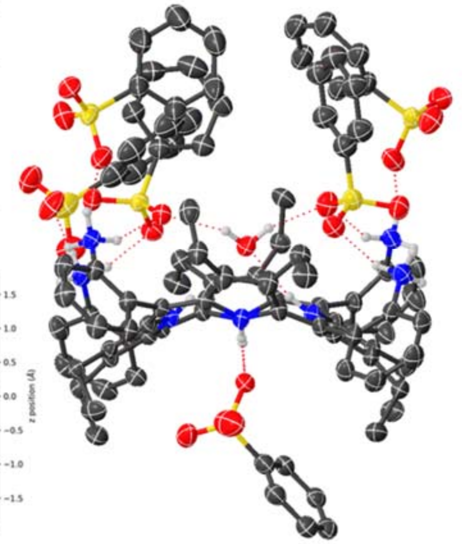

**Figure S11.** Top: out-of-plane NSD results of guest assisted sterically hindered porphyrin *α₄*-**P1**•$[C_6H_5SO_3^-]_6[H_2O][CD_3CN]_2$ [7]; bottom: out-of-plane and in-plane skeletal plots of the porphyrin[7] core. Porphyrin[7] is represented in black (C) and blue (N), with the reference structure (CuTPP) in red dotted lines.[4] Non-essential hydrogens and solvent molecules omitted for clarity, thermal ellipsoids shown at 50% probability.

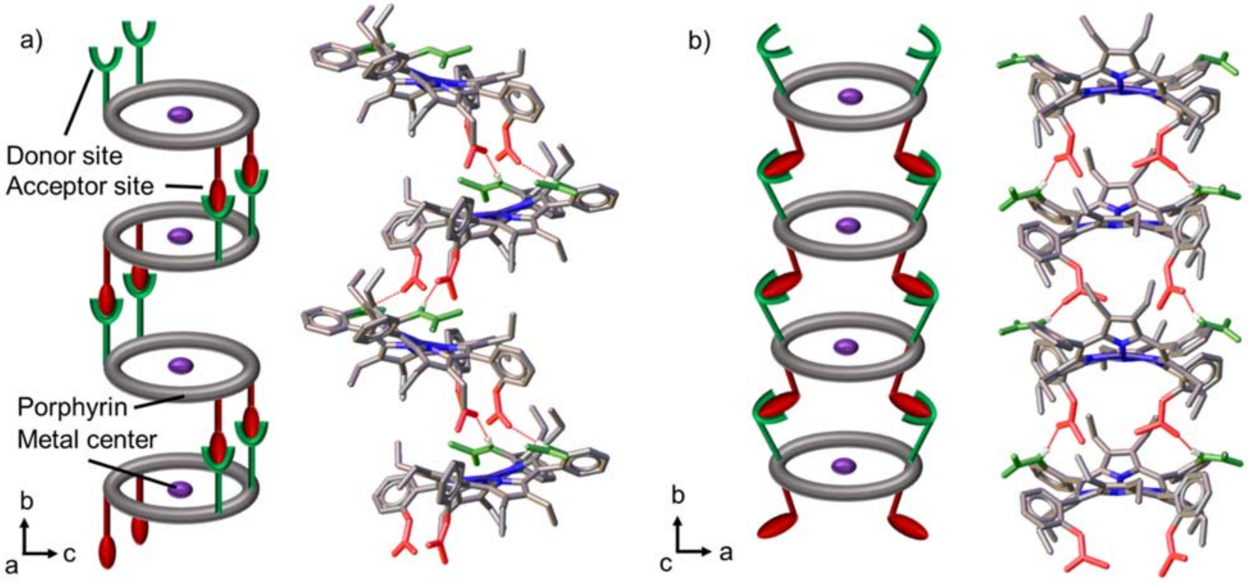


**Figure S12.** Schematic illustration of the intermolecular packing observed in $\alpha_2\beta_2$-**2**; a) and b) structural packing representation in different angles. Non-essential hydrogen atoms and solvent molecules were omitted for clarity, thermal ellipsoids shown at 50%. Red represents the amide groups as acceptors, while green – hydrogen bond donors.

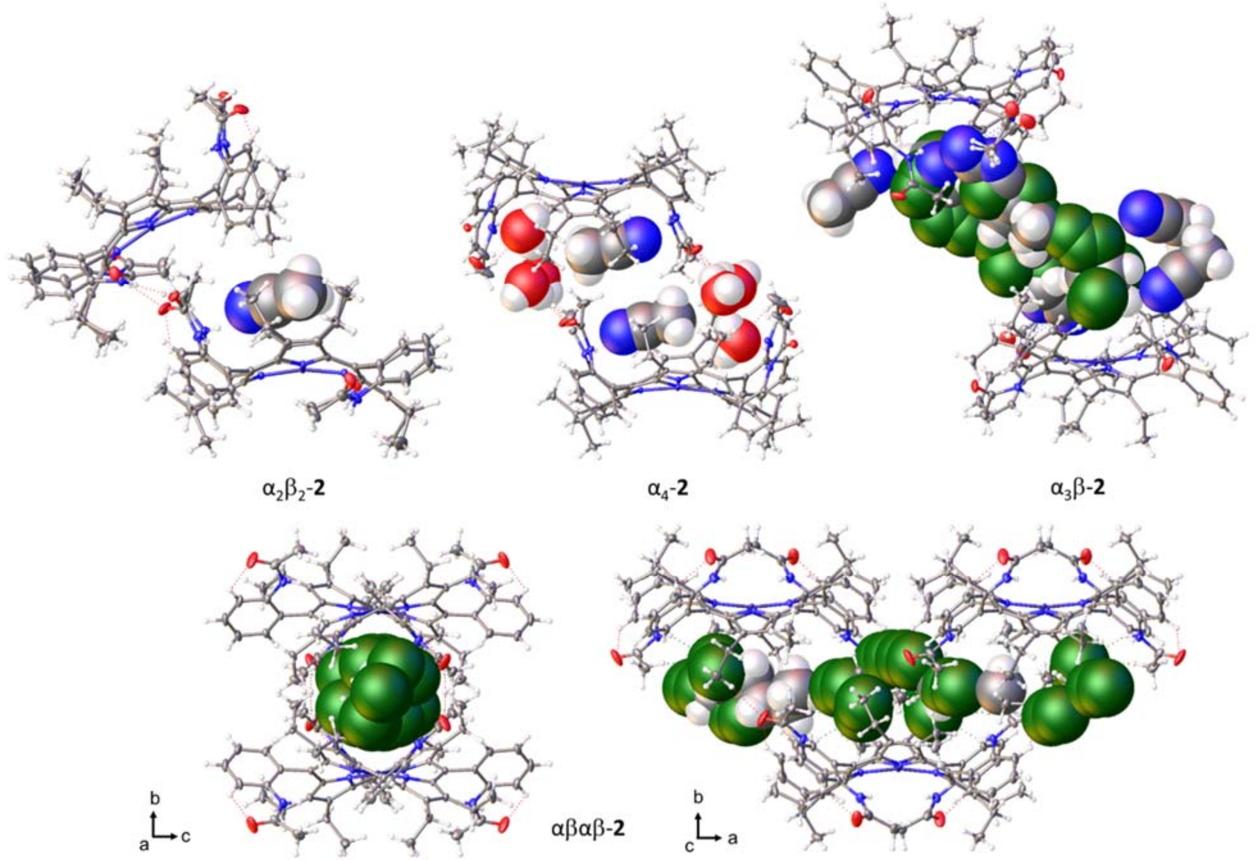


**Figure S13a.** Representation of the solvent-accessible voids of $\alpha_3\beta$-**2**; $\alpha_4$-**2** and $\alpha_2\beta_2$-**2** and parallel solvent channels in $\alpha\beta\alpha\beta$-**2**. Thermal ellipsoids shown at 50%.

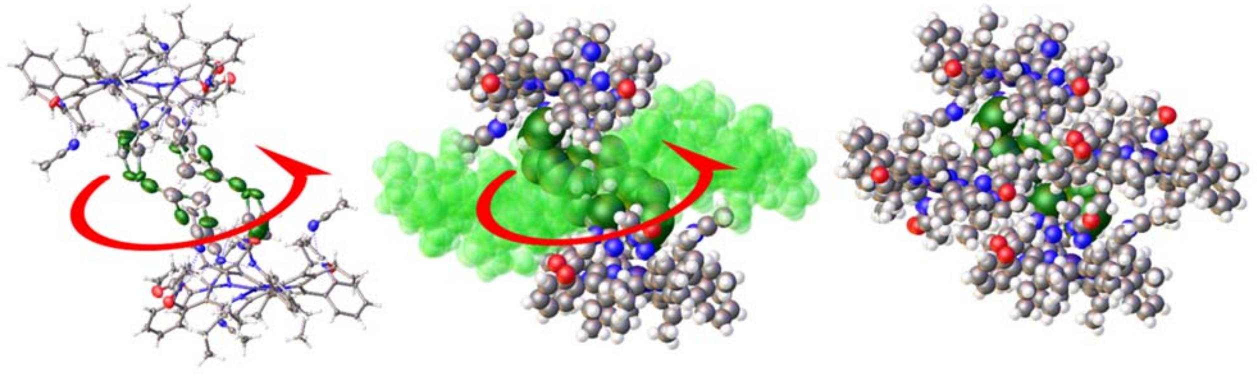

**Figure S13b.** Packing of the $\alpha_3\beta$-**2** highlighted in light green with the red arrow is 4 parallel porphyrin $\alpha_3\beta$-**2** units surrounding the solvent void. Thermal ellipsoids shown at 50%.

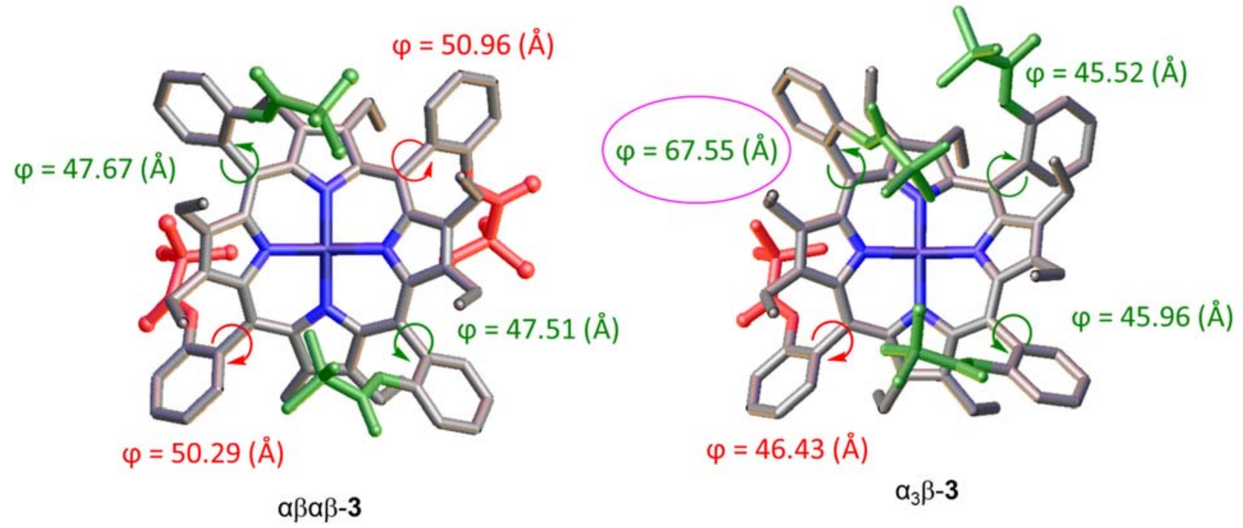


**Figure S14.** Plane-to-plane angle comparison of the phenyl rings to the 24-atom macrocycle plane in $\alpha_3\beta$-**3** and αβαβ-**3** structures. Highlighted in purple the angle of the phenyl in $\alpha_3\beta$-**3** showing aryl twist due to the severe steric repulsion forces. Non-essential hydrogen atoms and solvent molecules were omitted for clarity, thermal ellipsoids shown at 50%.

**$^{1}$H NMR analysis of isolated compounds**

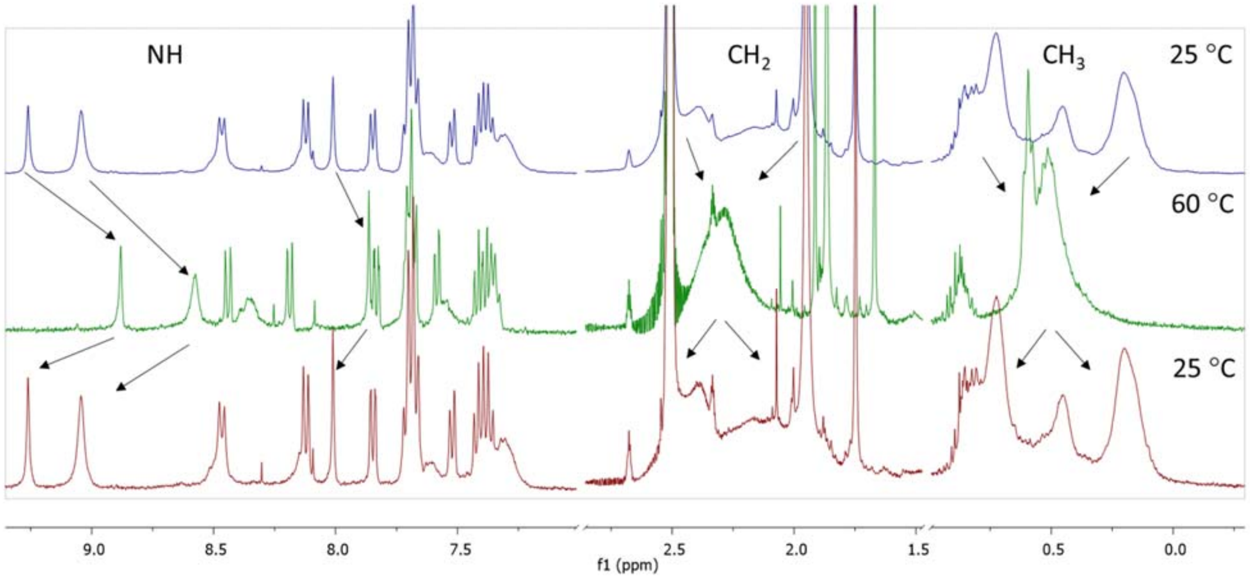


**Figure S15.** Variable temperature (to 60 °C) $^{1}$H NMR spectra of $\alpha_3\beta$-**2** (*d$_6$*-DMSO)

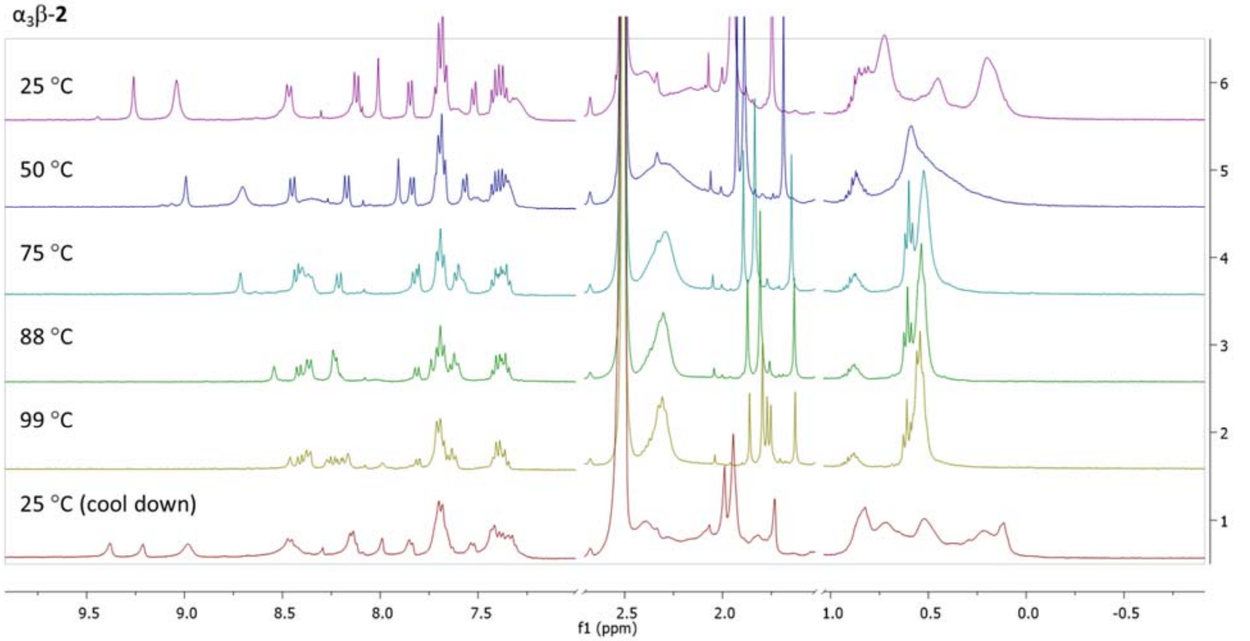


**Figure S16.** Variable temperature (to 100 °C) $^{1}$H NMR spectra of $\alpha_3\beta$-**2** (*d$_6$*-DMSO). Note, upon cooling down from 99 °C to 25 °C new spectral lines were observed indicating thermal interconversion.

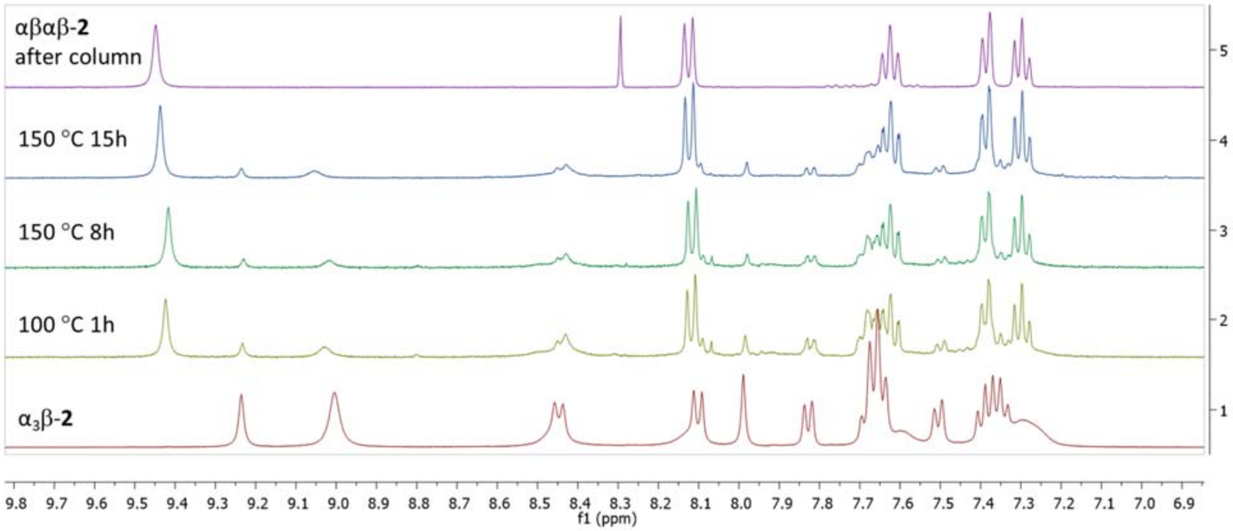


**Figure S17.** Aromatic region $^{1}$H NMR spectra of $\alpha_3\beta$-**2** ($d_6$-DMSO) recorded in various period of time and temperature. Note, stirring the $\alpha_3\beta$-**2** solution at 100 °C for 1 hour is sufficient for complete thermal enrichment, further exposer to higher temperatures and longer times had only marginal changes to the $^{1}$H NMR spectrum.

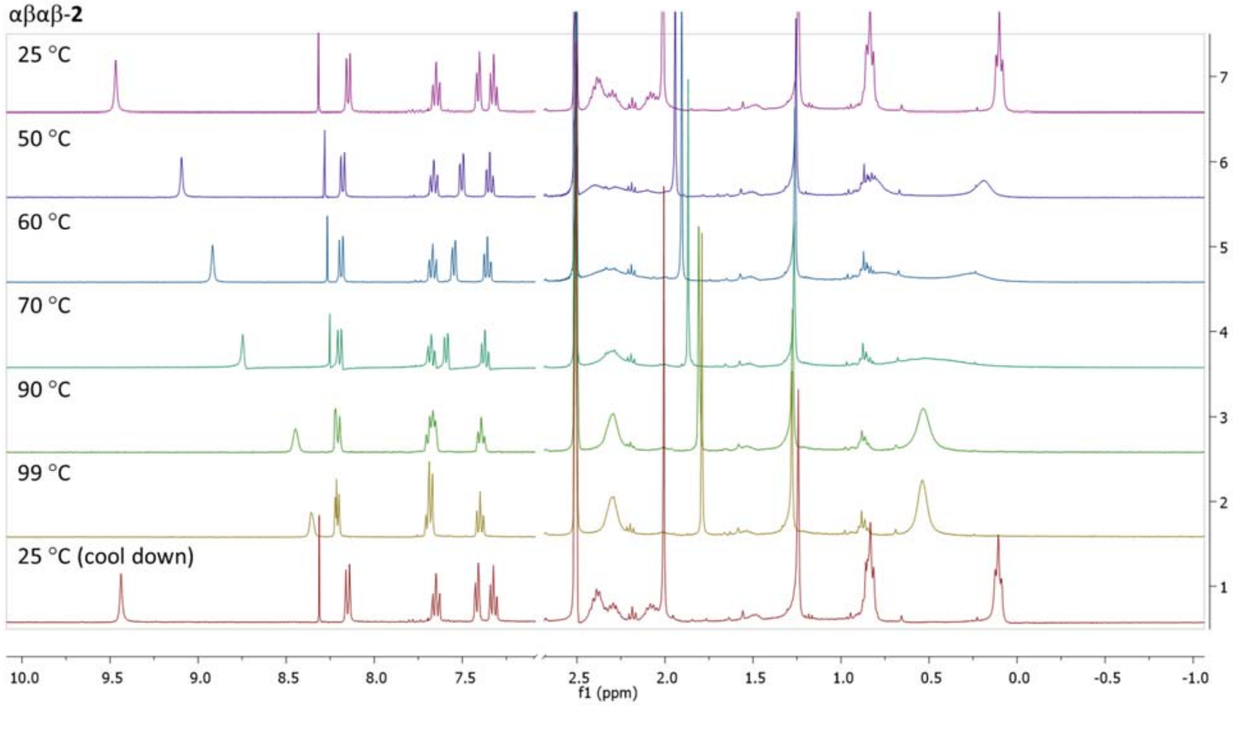


**Figure S18.** Variable temperature (to 99 °C) $^{1}$H NMR spectra of αβαβ-**2** ($d_6$-DMSO)

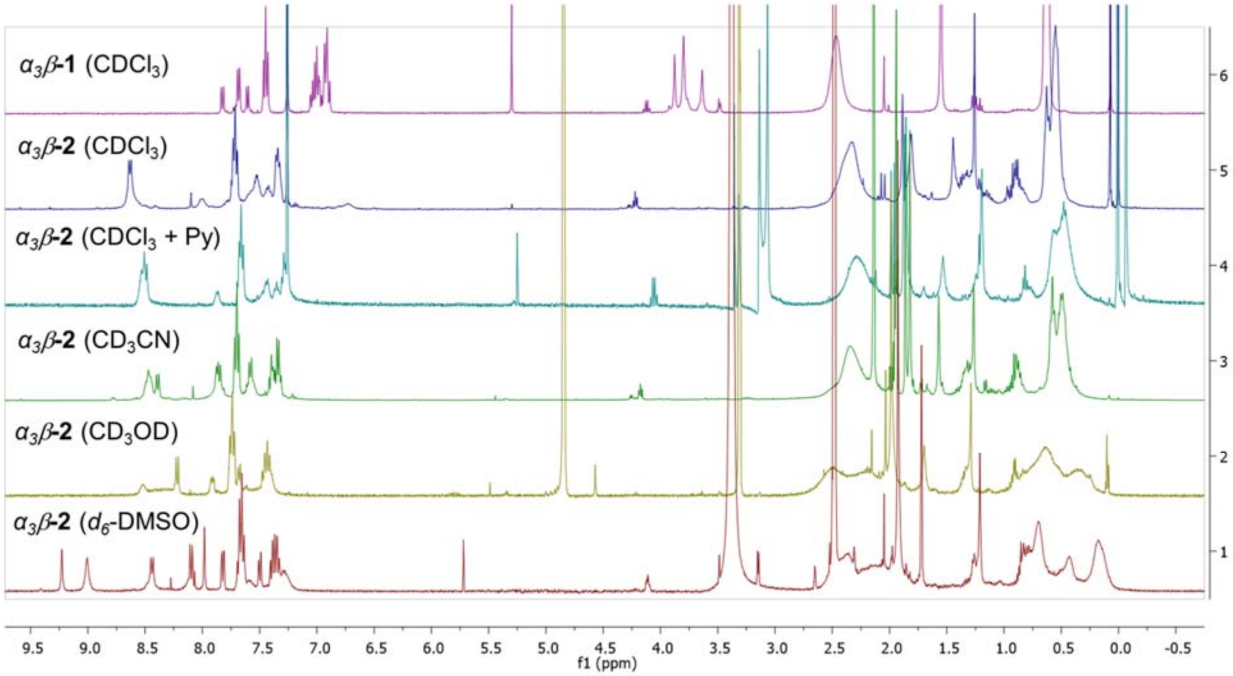


**Figure S19.** Overlay spectra of $\alpha_3\beta$-**2** and $\alpha_3\beta$-**1** recorded in various deuterated solvents.

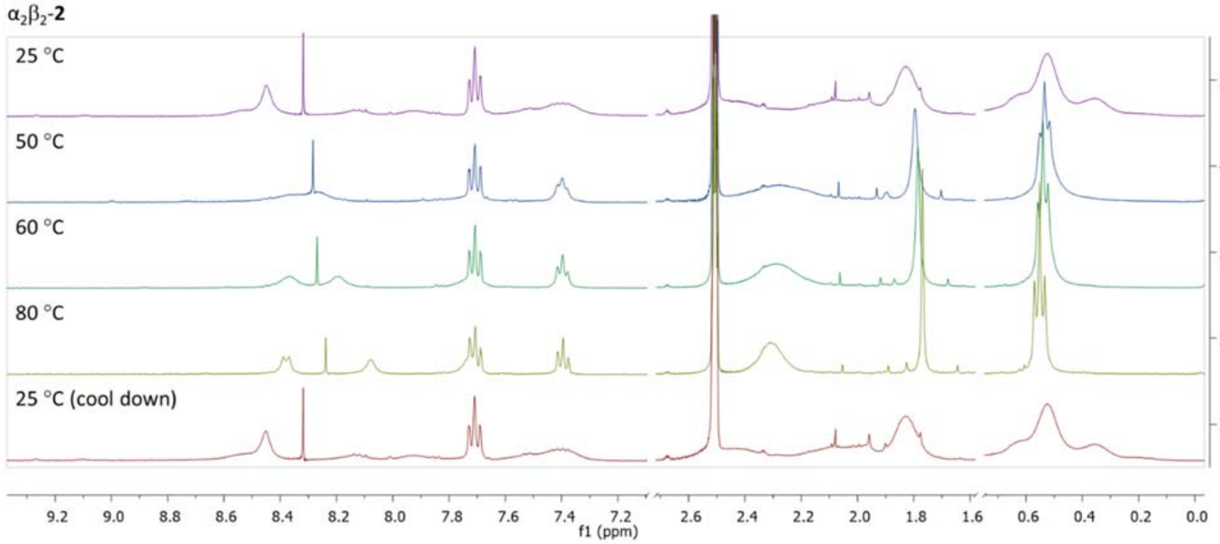


**Figure S20.** Variable temperature (to 80 °C) $^1$H NMR spectra of $\alpha_2\beta_2$-**2** ($d_6$-DMSO)

**HPLC analysis**

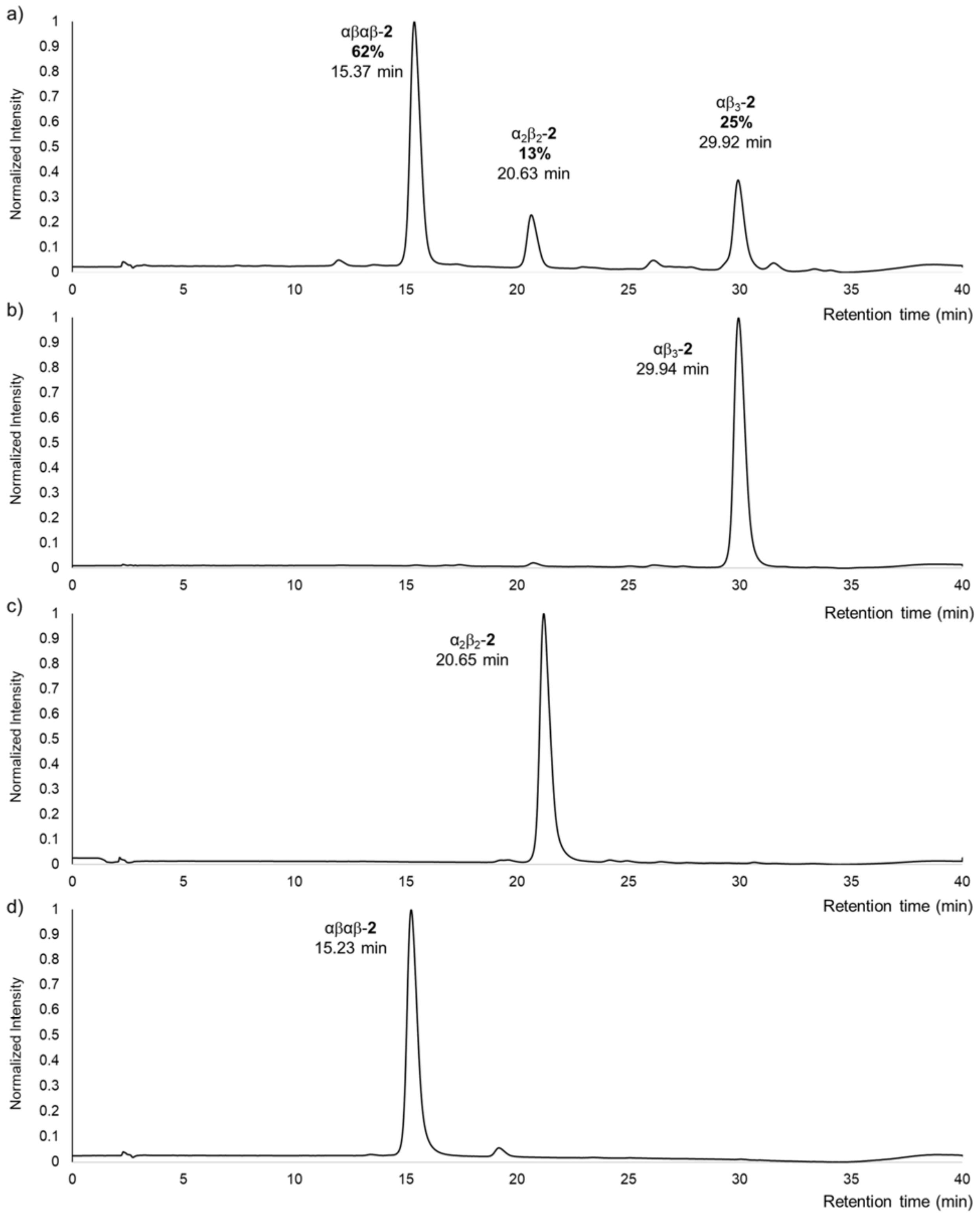


**Figure S21.** a) HPLC analysis of the $\alpha_3\beta$-**2** sample upon thermal interconversion (150 °C for 15h in *d*$_6$-DMSO); b) HPLC analysis of pure $\alpha_3\beta$-**2** sample; c) HPLC analysis of pure $\alpha_2\beta_2$-**2** sample; d) HPLC analysis of pure $\alpha\beta\alpha\beta$-**2** sample. Chromatograms recorded with detection at 450 nm.

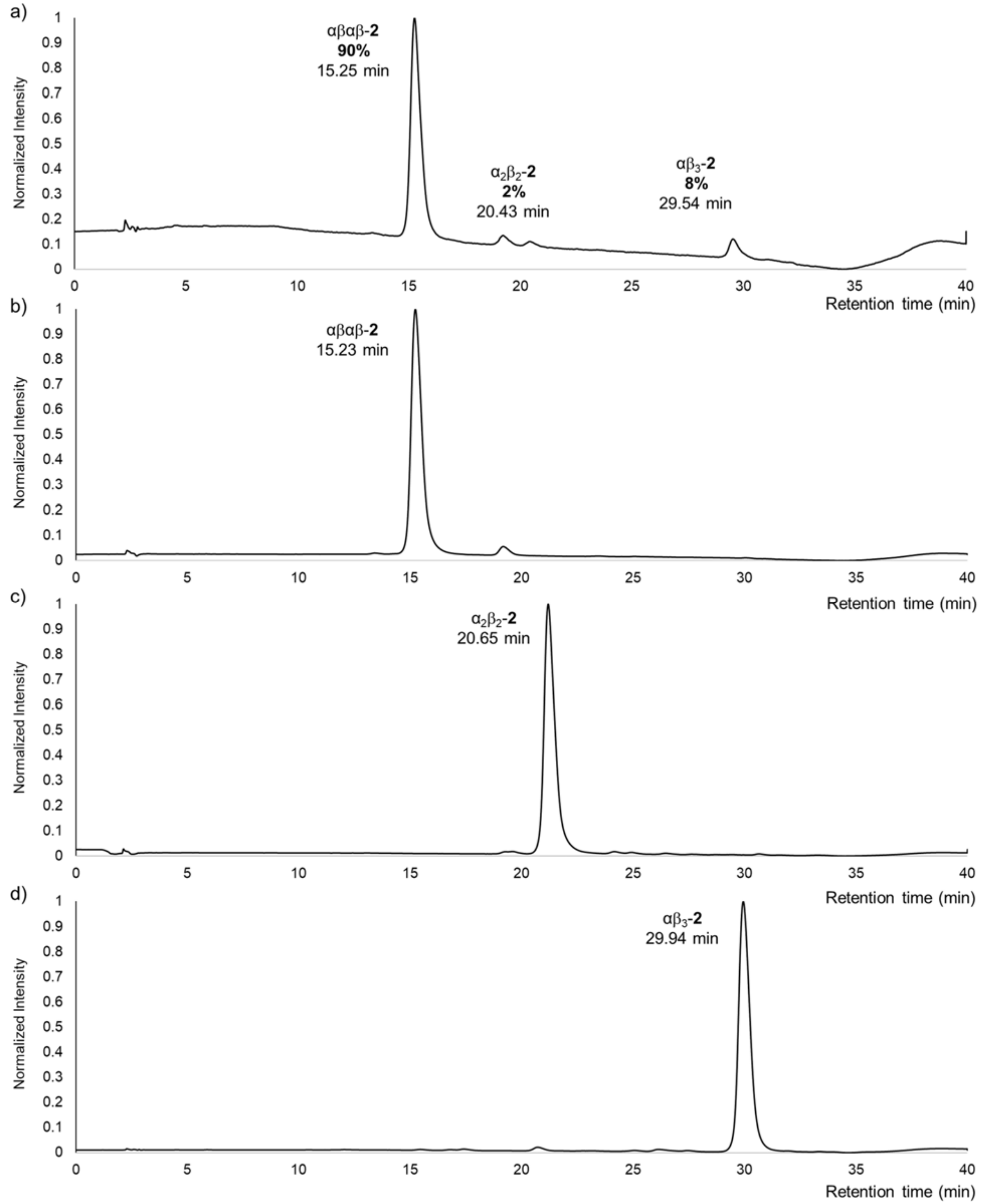


**Figure S22.** a) HPLC analysis of the $\alpha\beta\alpha\beta$-**2** sample used for thermal stability (100 °C for 1h in DMSO); b) HPLC analysis of pure $\alpha\beta\alpha\beta$-**2** sample; c) HPLC analysis of pure $\alpha_2\beta_2$-**2** sample; d) HPLC analysis of pure $\alpha_3\beta$-**2** sample. Note, vast majority of $\alpha_3\beta$-**2** was converted to other atropisomeric species by heating the sample at 100 °C for 1h in $d_6$-DMSO indicating very low thermal stability (see Figure S17). Chromatograms recorded with detection at 450 nm

**Monitoring α3β-3 reaction**

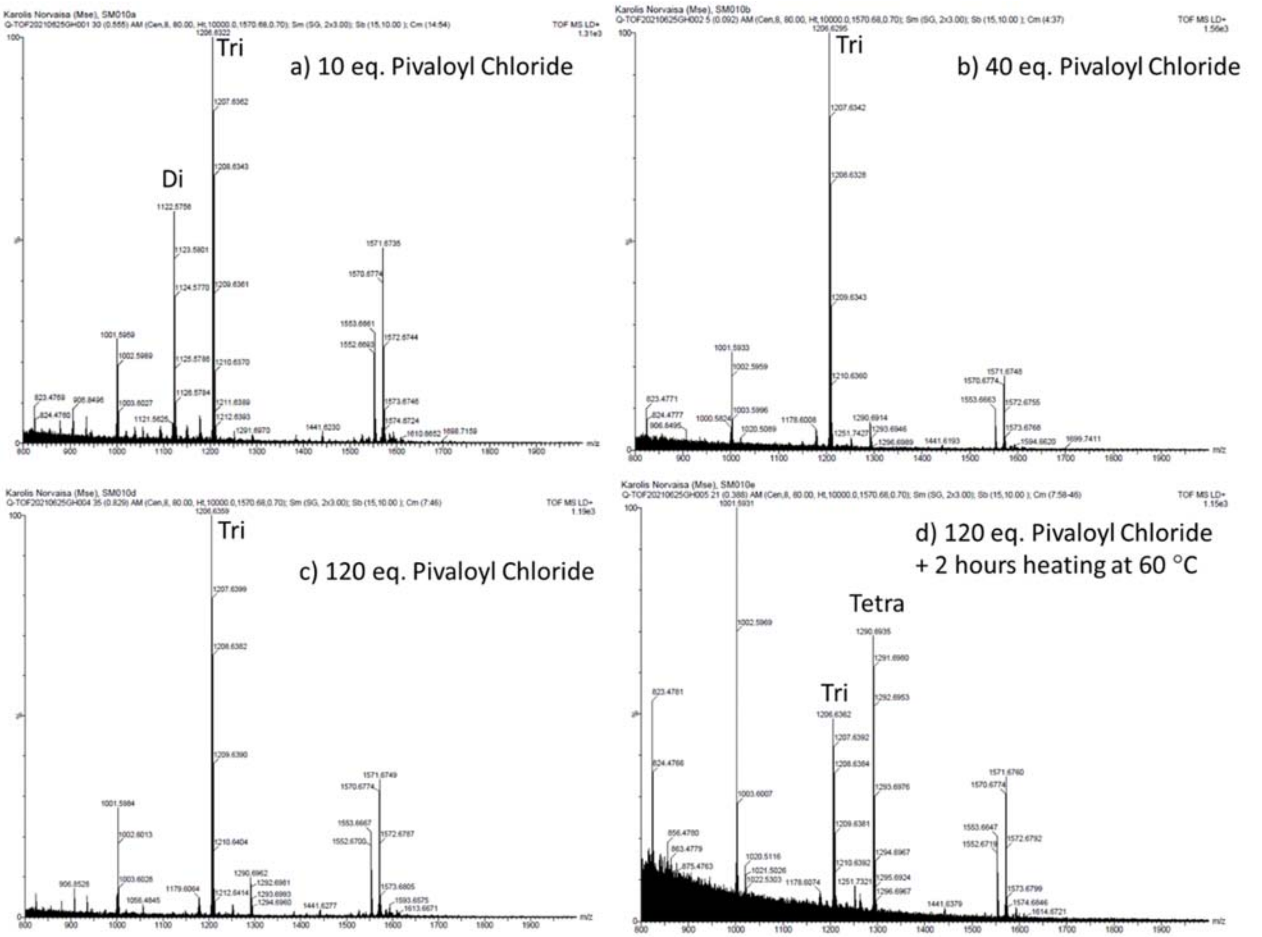


**Figure S23.** Monitoring $\alpha_3\beta$-**2** reaction by mass spectrometry (MALDI): a) upon addition of 10 eq. of pivaloyl chloride majority of di- and tri-substituted product has formed; b) upon addition of 40 eq. of pivaloyl chloride only tri substituted product has formed; b) after addition of 120 eq. of pivaloyl chloride yet still majority of tri substituted product was present with a trace amount of tetra-substituted product in the reaction mixture; d) only upon heating for 2 hours at 60 °C, tetra- with tri-substituted product was present in the solution.

**UV-vis spectrophotometry**

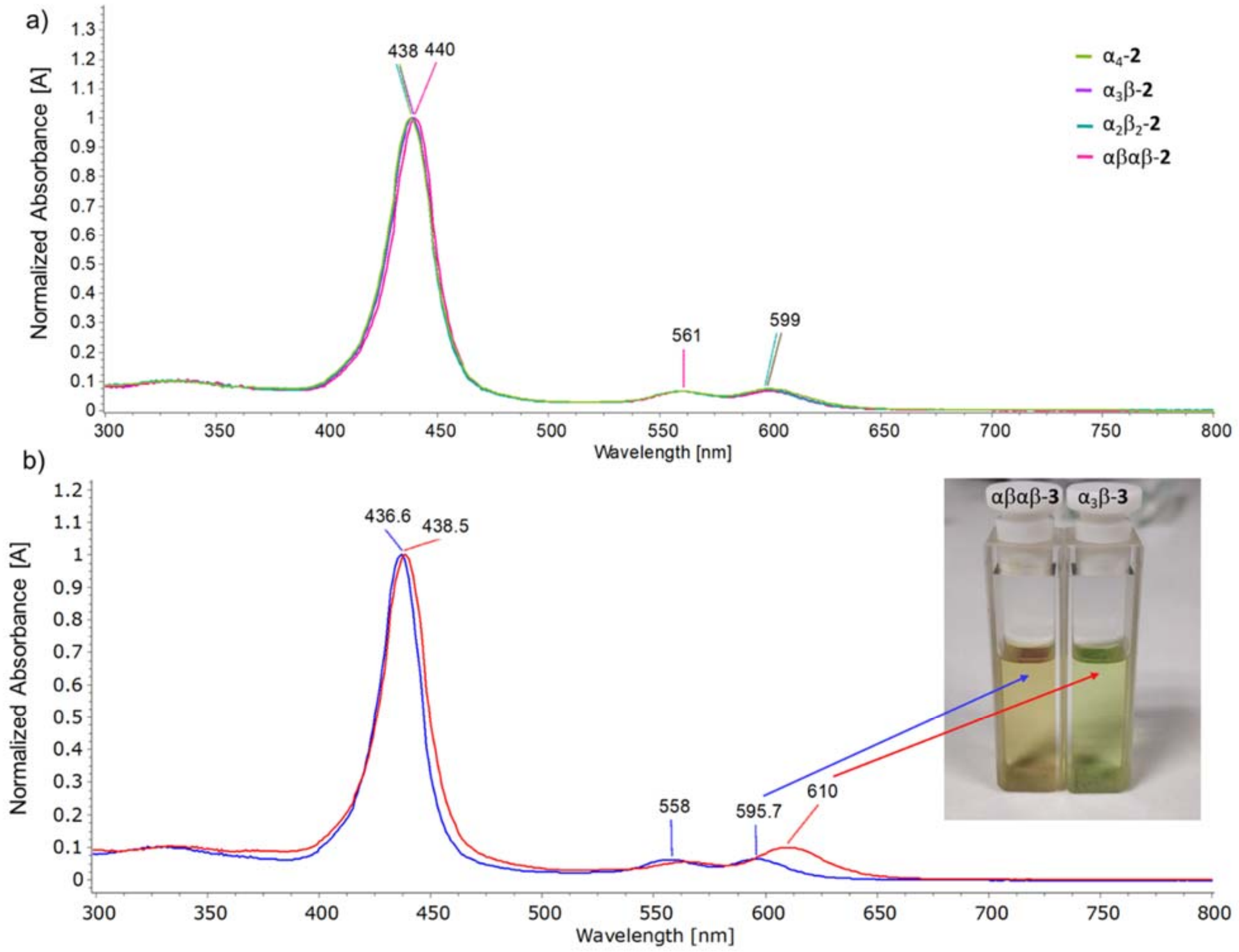


**Figure S24.** Normalized UV-vis spectra of a) $\alpha_3\beta$-**2**, $\alpha\beta\alpha\beta$-**2**, $\alpha_4$-**2**, and $\alpha_2\beta_2$-**2**; b) $\alpha_3\beta$-**3** and $\alpha\beta\alpha\beta$-**3** with illustration highlighting the colorimetric differences. Recorded in $CHCl_3$

**Experimental Procedures**

**General Materials and Methods**

All chemicals were supplied by Sigma Aldrich, Acros Organics, Fluka, Frontier Scientific, and Fischer and handled without further purification unless otherwise stated. Anhydrous DCM used in large scale reactions was obtained *via* drying with phosphorus pentoxide followed by distillation, while smaller amounts of $CHCl_3$ and $C_6H_4Cl_2$ were used as commercially available HPLC grade solvents. Reactions involving moisture and/or air-sensitive reagents were carried out in pre-dried glassware and with standard Schlenk line techniques. The 3,4-diethyl-1H-pyrrole **11**[8] and [2,3,7,8,12,13,17,18-octaethyl-5,10,15,20-tetrakis(2-aminophenyl)porphyrin] **1**[9] was prepared *via* known procedures. Yields refer to chromatographically and spectroscopically ($^1$H NMR) homogeneous material, unless otherwise noted. Reactions were monitored by thin layer chromatography (TLC) and absorption spectroscopy.

**Analytical thin layer chromatography** was performed using silica gel 60 (fluorescence indicator F254, precoated sheets, 0.2 mm thick, 20 cm × 20 cm; Merck) or aluminum oxide 60 (neutral, F254; Merck) plates and visualized by UV irradiation ($\lambda$ = 254 nm).

**Column chromatography** was carried out using Fluka Silica Gel 60 (230–400 mesh; Merck) or aluminum oxide (neutral, activated with 6% $H_2O$, Brockman Grade III). Mobile phases are given as (*v/v*).

**Mass spectrometry** was performed with a Q-Tof Premier Waters MALDI quadrupole time-of-flight (Q-TOF) mass spectrometer equipped with Z-spray electrospray ionization (ESI) and matrix-assisted laser desorption ionization (MALDI) sources in positive mode with DCTB *trans*-2-[3-(4-*tert*-butylphenyl)-2-methyl-2-propenylidene]malononitrile (DCTB) as the matrix. ESI mass spectra were acquired in positive modes as required, using a Micromass time-of-flight mass spectrometer (TOF) interfaced to a Waters 2960 HPLC or a Bruker microTOF-Q III spectrometer interfaced to a Dionex UltiMate 3000 LC. Atmospheric pressure chemical ionization (APCI) experiments were performed on a Bruker microTOF-Q III spectrometer interfaced to a Dionex UltiMate 3000 LC.

**Melting points** are uncorrected and were measured with a Stuart SP-10 melting point apparatus.

**NMR spectra** were recorded on a Bruker Advance III 400 MHz, a Bruker Advance HD 400 and an Agilent 400 spectrometer for $^1$H (400.13 MHz) and $^{13}$C (100.61 MHz) NMR spectra. A Bruker Ultrashield 600 spectrometer was employed for $^1$H (600.13 MHz) and $^{13}$C (150.90 MHz) NMR spectra. All NMR experiments were performed at 25 °C. Resonances $\delta$ are given in ppm units and referenced to the deuterium peak in the NMR solvents, $d_4$-methanol ($\delta_H$ = 4.87, 3.31 ppm, $\delta_C$ = 49.1 ppm). $CDCl_3$ ($\delta_H$ = 7.26 ppm, $\delta_C$ = 77.2 ppm). Signal multiplicities are abbreviated as follows: singlet = s, doublet = d, triplet = t, multiplet = m, quintet = qui.

**Single crystal X-ray crystallography:** Diffraction data for all compounds were collected on a Bruker D8 Quest ECO or Bruker APEX 2 DUO CCD diffractometer using graphite-monochromated Mo-$K_\alpha$ ($\lambda$ = 0.71073 Å) or Incoatec IμS Cu-$K_\alpha$ ($\lambda$ = 1.54178 Å) radiation. Crystals were mounted on a MiTeGen MicroMount and collected at 100(2) K using an Oxford Cryosystems Cobra low-temperature device. Data were collected using omega and phi scans and were corrected for Lorentz and polarization effects using the APEX software suite.[10] Data were corrected for absorption effects using the multi-scan method (SADABS).[11]

**UV-Vis absorption measurements** were recorded in solutions using a Specord 250 spectrophotometer from Quartz Glass 10mm 6030-UV (1 cm path length quartz cell).

**IR measurements** were done on a PerkinElmer Spectrum 100 FT-IR.

**Shelnutt's NSD** (normal structural decomposition) method was used to delineate, quantify and illustrate the various distortions modes present in the tetrapyrrole macrocycles.[12] NSD calculations were performed with the NSD GUI version of the program.[13] Analysis was performed with the NSD online interface, available at https://www.sengegroup.eu/nsd.[4]

**HPLC**: Samples were dissolved in a solution of HPLC grade MeCN and ammonium acetate buffer (100 mM, pH 8-9) (6:4) and further submitted to Reverse Phase-HPLC analysis (Varian 920-LC Integrated Analytical HPLC) using a Phenomenex® reverse phase C18 column (4.6 x 25mm, 5μM) at 20-22 °C. A gradient of 80 to 95% MeCN with ammonium acetate buffer was used over 30min at a constant flow rate of 1 mL/min. The atropisomers were detected at 450 nm.

**Synthesis and Characterization of Compounds**

*Synthesis and characterization of $\alpha_3\beta$-**2***

**[$\alpha_3\beta$-5,10,15,20-Tetrakis(2-*N*-acetamidephenyl)-2,3,7,8,12,13,17,18-octaethylporphyrinato]nickel(II) [$\alpha_3\beta$-2]**

In a 50mL round bottom flask containing $\alpha_3\beta$-**1** ([$\alpha_3\beta$-5,10,15,20-tetrakis(2-aminophenyl)-2,3,7,8,12,13,17,18-octaethylporphyrinato]nickel(II)) (59.5mg, 62.24µmol, 1 eq.) was dissolved in chloroform (10mL). Acetyl chloride (178µL, 2.489 mmol, 40 eq.) was added in 10 eq. additions following 15 eq. additions of *N,N*-diisopropylethylamine (696µL, 3.734mmol, 60 eq.). Reaction was stirred at room temperature for 3hrs and monitored by TLC (Dichloromethane: ethyl acetate 10:1 v/v). Solvent was removed under reduced pressure, the purple solid was dissolved in dichloromethane and transferred to column chromatography ($SiO_2$, dichloromethane: ethyl acetate 5:1 v/v). The second dark green band was collected and recrystallized in acetonitrile, leading to a purple crystalline solid product [64.6mg, 57.47µmol, 92%]. M.p > 300 °C. Rf = 0.48 ($SiO_2$, dichloromethane: ethyl acetate 2:1 v/v. $^1$H NMR (600 MHz, $d_6$-DMSO, 25 °C) δ 9.26 (s, 1H, N-H), 9.03 (s, 2H, N-H), 8.47 (d, *J* = 7.8 Hz, 2H, Ar-H), 8.13 (d, *J* = 8.2 Hz, 2H, Ar-H), 8.01 (s, 1H, N-H), 7.85 (d, *J* = 6.8 Hz, 1H, Ar-H), 7.74 – 7.65 (m, 4H, Ar-H), 7.62 (s, 1H, Ar-H), 7.53 (d, *J* = 7.2 Hz, 1H, Ar-H), 7.41 (t, *J* = 7.4 Hz, 2H, Ar-H), 7.38 (t, *J* = 7.3 Hz, 2H, Ar-H), 7.30 (d, *J* = 24.4 Hz, 1H), 2.46 – 2.03 (m, 16H, -$CH_2$), 1.96 (s, 12H, Ac-$CH_3$), 1.75 (s, 3H), 0.74 – 0.16 (m, 24H, -$CH_3$). $^{13}$C NMR (151 MHz, $d_6$-DMSO, 25 °C) δ 168.75, 147.96, 147.33, 147.18, 146.39, 145.15, 144.68, 144.32, 143.66, 143.24, 142.91, 142.43, 138.93, 138.53, 135.95, 135.36, 129.88, 129.56, 129.43, 128.64, 123.55, 123.45, 123.15, 113.07, 110.86, 24.54, 23.70, 19.37, 18.47, 16.10. UV/Vis (Chloroform): λ max (log ε) = 438 (5.31), 561 (4.14), 599 (4.17); HRMS (MALDI) m/z calc. for $C_{68}H_{72}N_8O_4Ni$ $[M]^+$: 1122.5030, found 1122.5043; IR (ATR): ṽ = 3401, 3058, 2970, 2930, 2871, 1683, 1604, 1579, 1513, 1436, 1368, 1294, 1256, 1228, 1163, 1135, 1104, 1051, 1021, 994.4, 962.3, 889.3, 849.1, 799.2, 156.3, 730.0, 652.9.

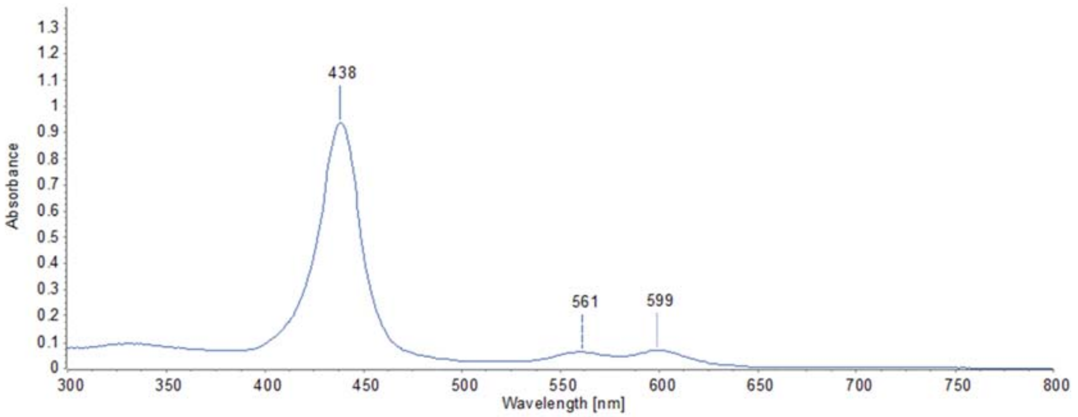


**Figure S25.** UV-vis spectrum of $\alpha_3\beta$-**2** in $CHCl_3$ [4.56µM].

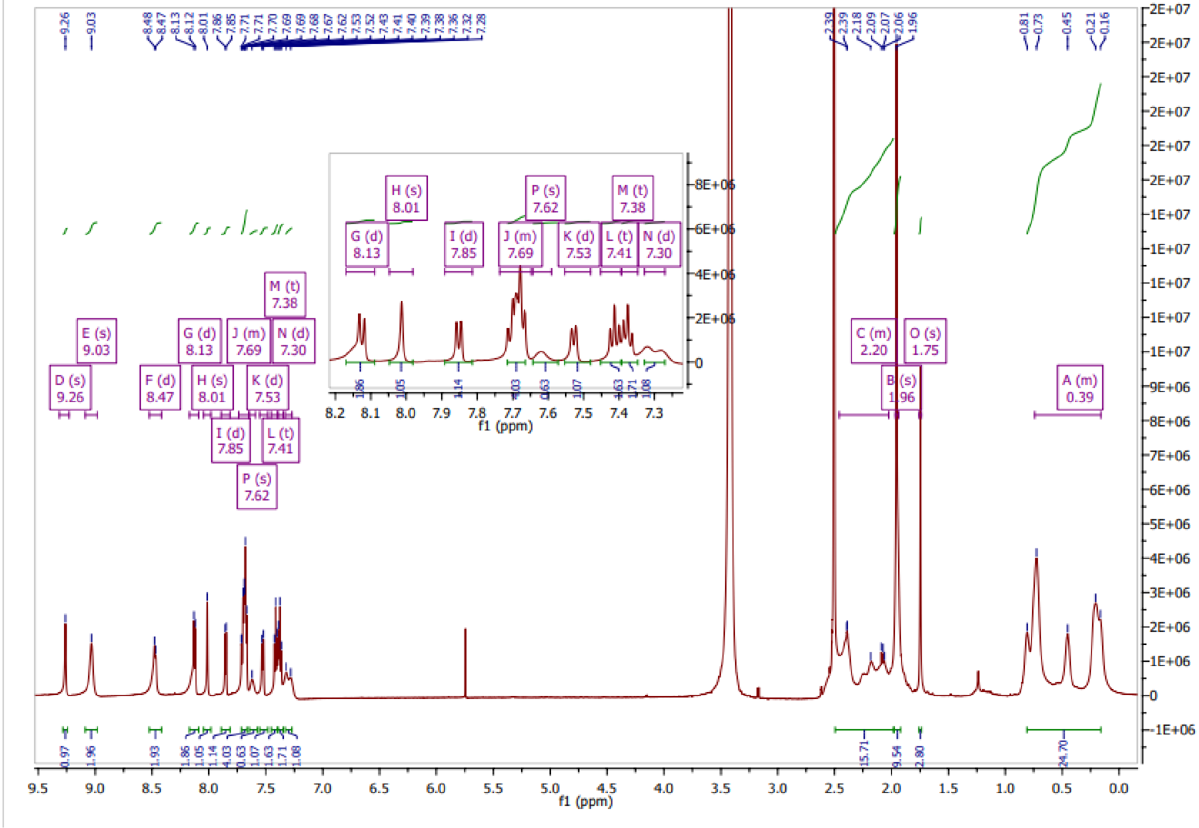


**Figure S26.** ¹H NMR spectrum of *α₃β*-**2** with expansion of areas of interest (600 MHz, *d₆*-DMSO, 25°C).

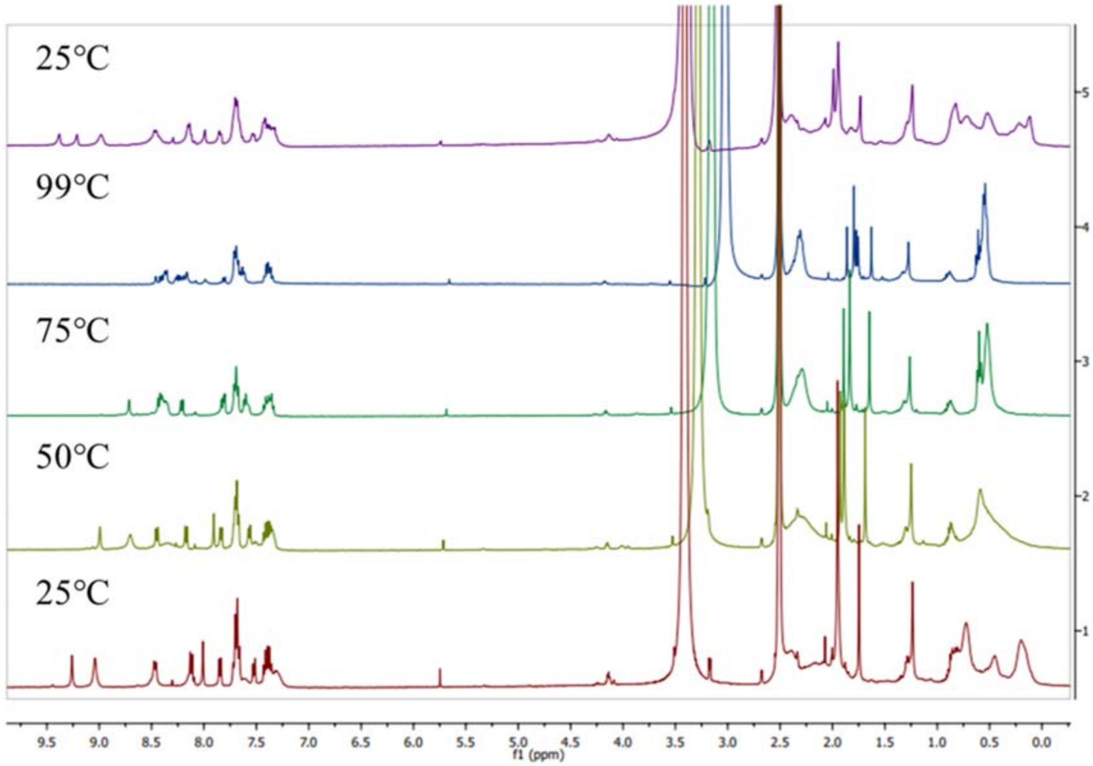


**Figure S27.** ¹H NMR VT spectra of *α₃β*-**2** (400 MHz, *d₆*-DMSO).

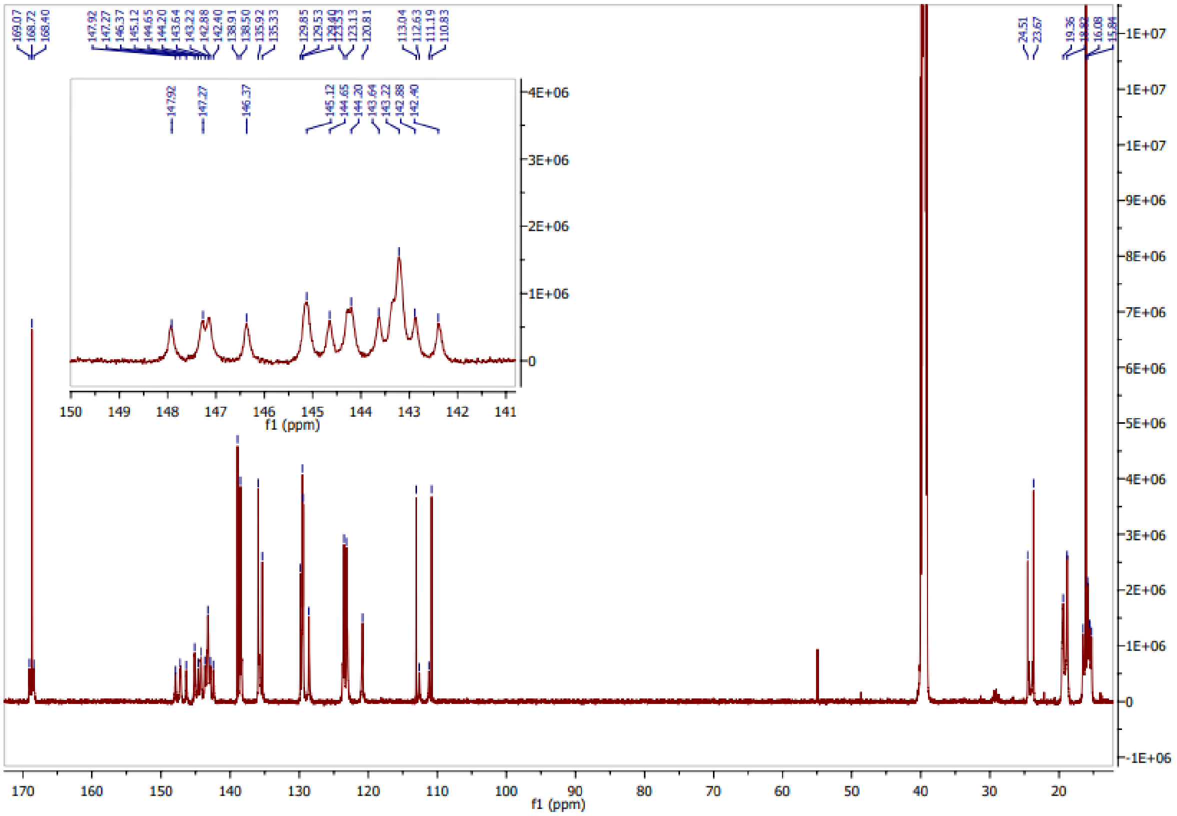

**Figure S28.** $^{13}C$ NMR spectrum of α$_3$β-**2** with expansion of areas of interest (151 MHz, *d*$_6$-DMSO, 25°C).

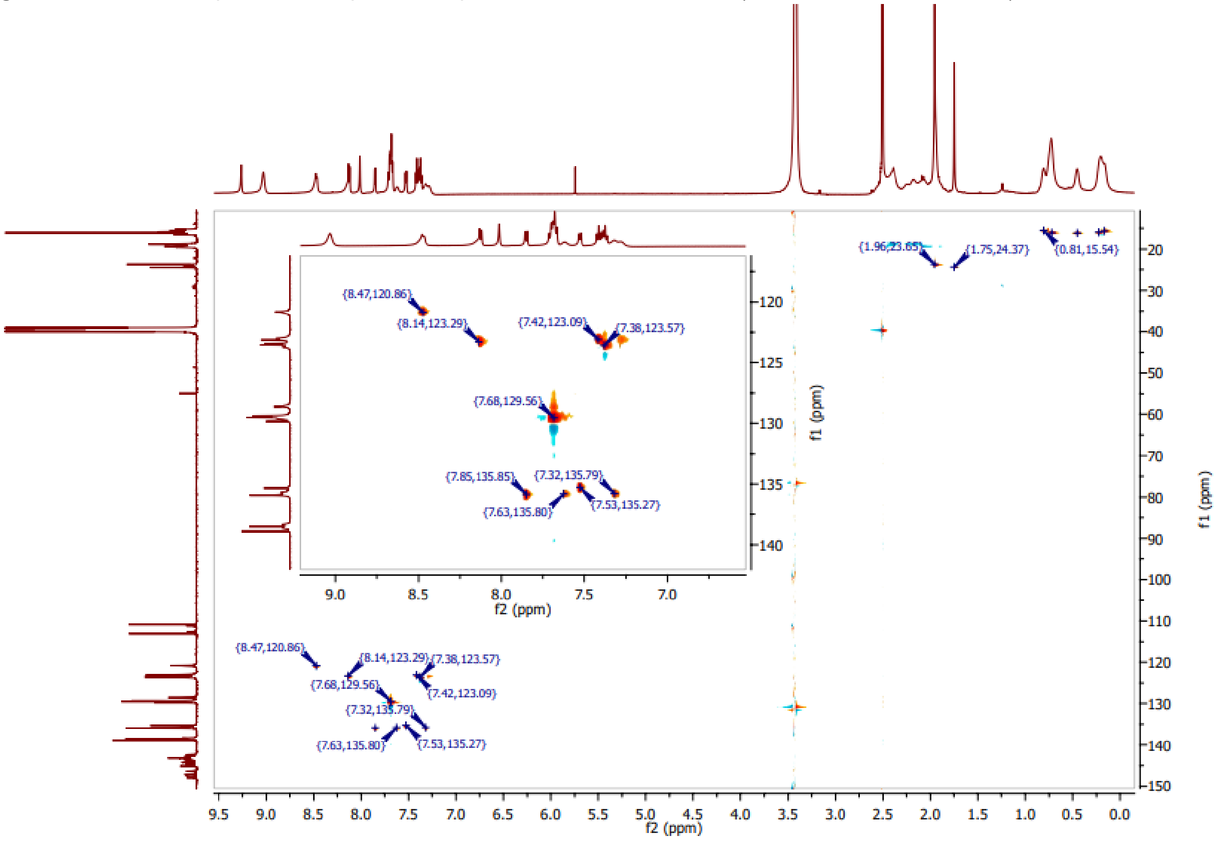

**Figure S29.** $^{1}H$–$^{13}C$ HSQC spectrum of α$_3$β-**2** with expansion of areas of interest (*d*$_6$-DMSO, 25°C).

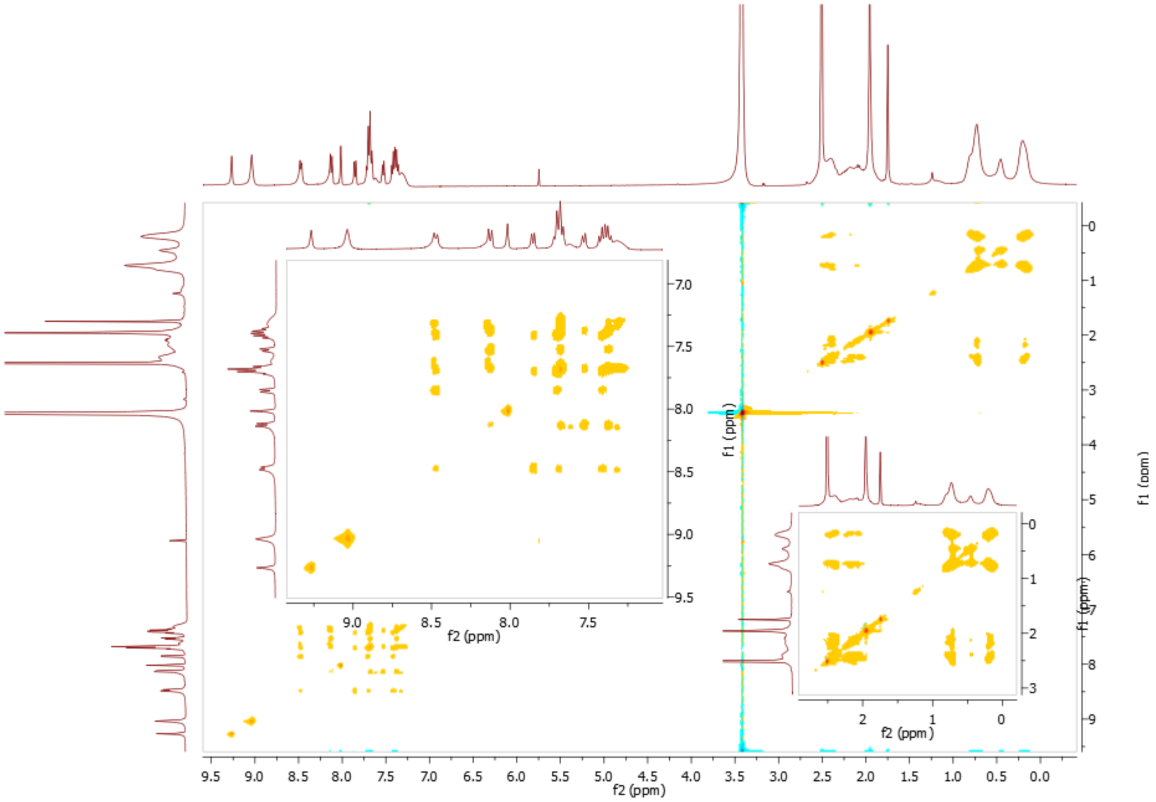


**Figure S30.** $^{1}H$–$^{1}H$ TOCSY spectrum of α$_3$β-**2** with expansion of areas of interest ($d_6$-DMSO, 25°C).

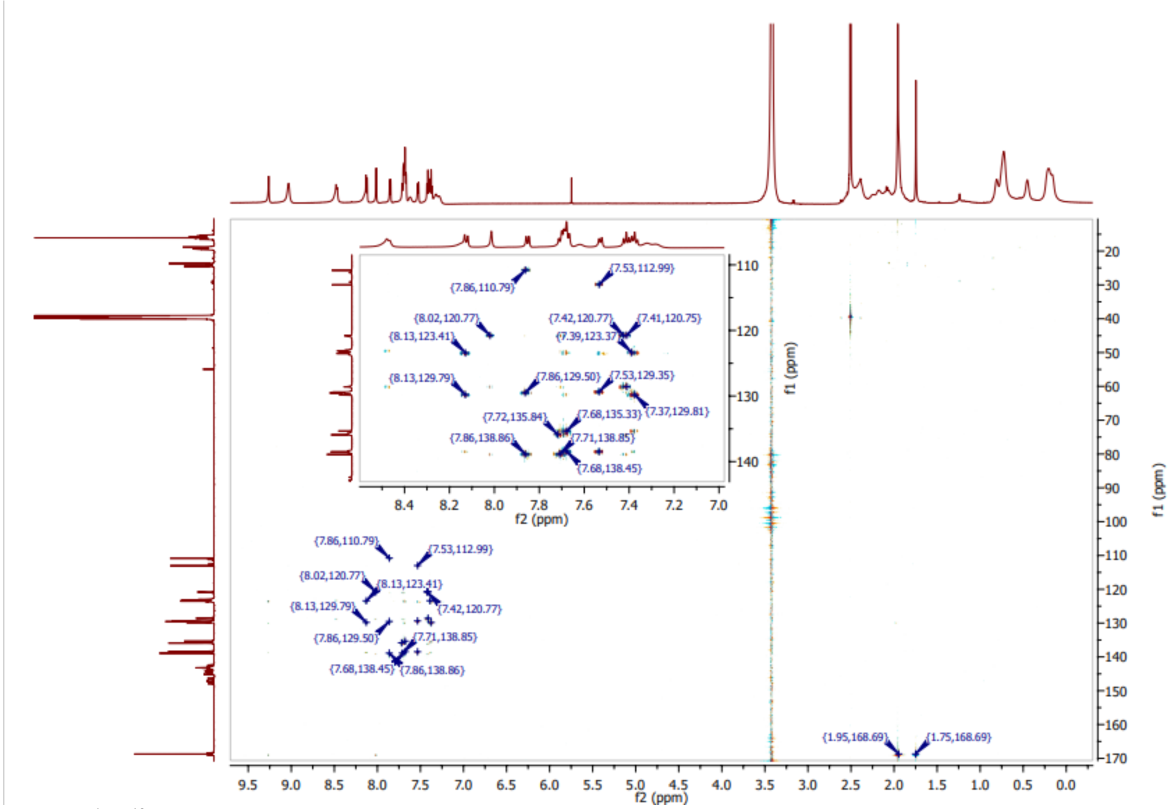


**Figure S31.** $^{1}H$–$^{13}C$ HMBC spectrum of α$_3$β-**2** with expansion of areas of interest ($d_6$-DMSO, 25°C).

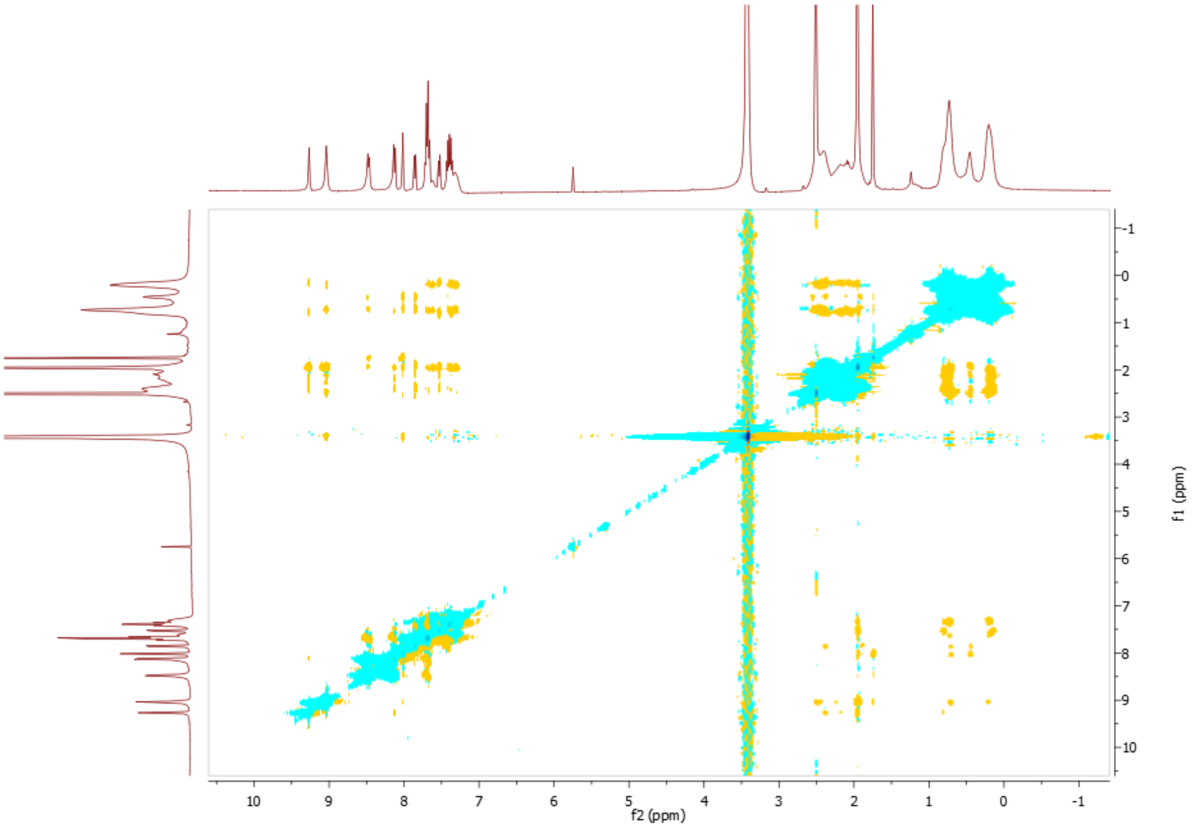


**Figure S32.** $^{1}H$–$^{1}H$ ROESY spectrum of α$_3$β-**2** with expansion of areas of interest (*d*$_6$-DMSO, 25°C).

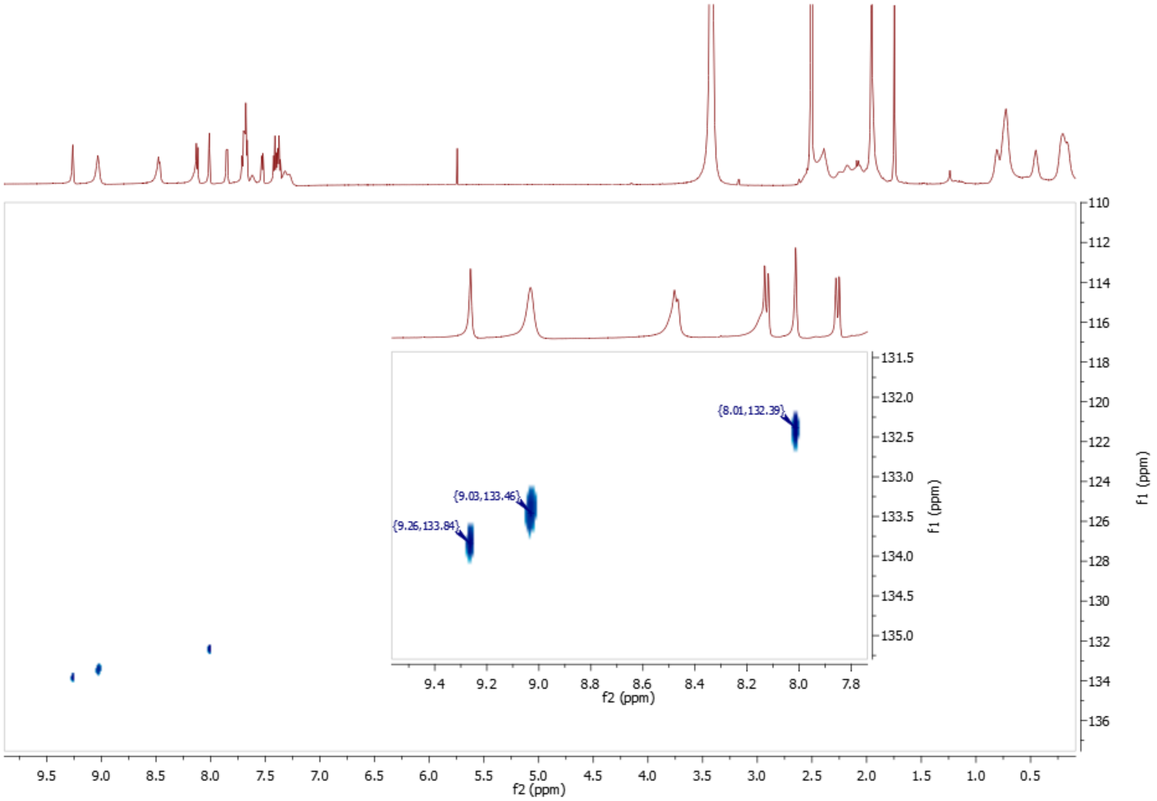


**Figure S33.** $^{1}H$–$^{15}N$ HSQC spectrum of α$_3$β-**2** with expansion of areas of interest (*d*$_6$-DMSO, 25°C).

**Elemental Composition Report** **Page 1**

**Single Mass Analysis**
Tolerance = 50.0 PPM / DBE: min = -1.5, max = 400.0
Element prediction: Off
Number of isotope peaks used for i-FIT = 5

Monoisotopic Mass, Odd and Even Electron Ions
68 formula(e) evaluated with 1 results within limits (up to 10 closest results for each mass)
Elements Used:
C: 0-68 H: 0-72 N: 0-8 O: 0-4 Ni: 0-1

Karolis Norvaisa (MSe), SM001
Q-TOF20210616GH001 69 (1.626) AM (Cen,8, 80.00, Ht,10000.0,1570.68,0.70); Sm (SG, 2x3.00); Sb (15,10.00 ); Cm (11:91-68:70) TOF MS LD+ 1.33e+003

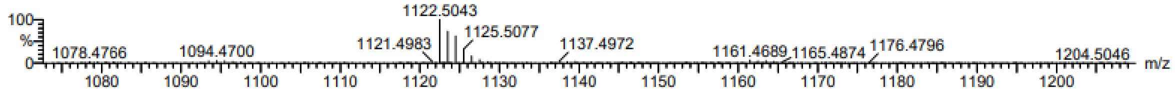


| | | | | | | | |
|---|---|---|---|---|---|---|---|
| Minimum: | | | | -1.5 | | | |
| Maximum: | | 5.0 | 50.0 | 400.0 | | | |
| Mass | Calc. Mass | mDa | PPM | DBE | i-FIT | i-FIT (Norm) | Formula |
| 1122.5043 | 1122.5030 | 1.3 | 1.2 | 37.5 | 53.0 | 0.0 | C68 H72 N8 O4 Ni |

**Figure S34.** HRMS (MALDI) of α$_3$β-**2**.

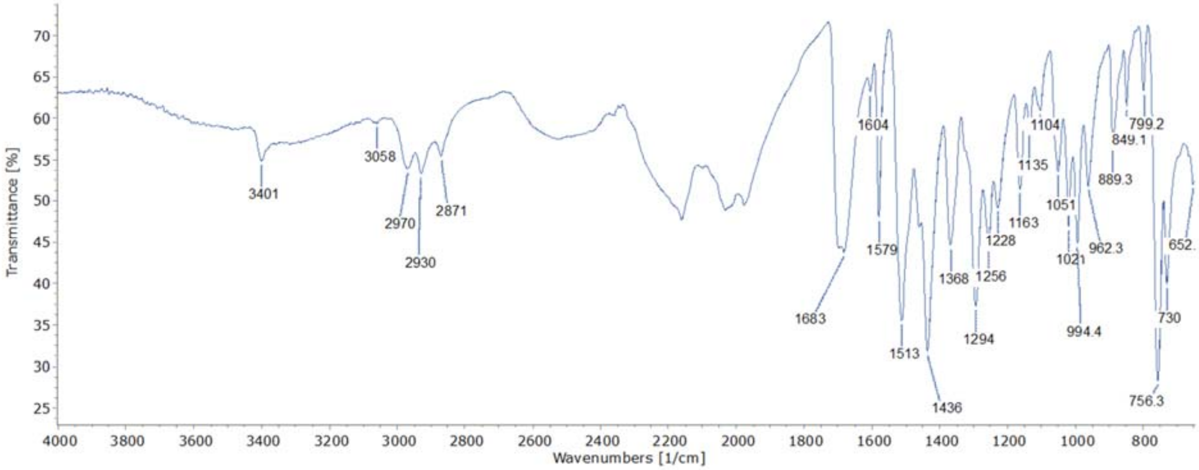


**Figure S35.** FTIR spectrum of α$_3$β-**2**.

*Synthesis and characterization of αβαβ-**2***

**[*αβαβ*-5,10,15,20-Tetrakis(2-*N*-acetamidephenyl)-2,3,7,8,12,13,17,18-octaethylporphyrinato]nickel(II) [*αβαβ-2*]**

In 50mL round bottom flask, ([$\alpha_3\beta$-5,10,15,20-tetrakis(2-aminophenyl)-2,3,7,8,12,13,17,18-octaethylporphyrinato]nickel(II)) (18.7mg, 16.64mmol) was dissolved in DMSO (1.5mL) then heated for 15hrs at 120°C. The solution was washed with saturated NaCl solution in 50 mL of dichloromethane and dried over $MgSO_4$. Upon removal of the solvents, the mixture was re-dissolved in dichloromethane and transferred for column chromatography ($SiO_2$, dichloromethane: ethyl acetate 5:1 v/v). The first dark green band was collected, before removing solvent under reduced pressure. Product was recrystallised in acetonitrile, giving a purple crystalline solid [7.6mg, 6.76µmol, 41%]. M.p > 300 °C. Rf = 0.8 ($SiO_2$, dichloromethane: ethyl acetate 2:1 v/v). $^1$H NMR (600 MHz, $d_6$-DMSO, 25 °C) δ 9.46 (s, 4H, N-H), 8.14 (d, *J* = 8.2 Hz, 4H, Ar-H), 7.64 (t, *J* = 7.8 Hz, 4H, Ar-H), 7.41 (d, *J* = 6.7 Hz, 4H, Ar-H), 7.32 (t, *J* = 7.8 Hz, 4H, Ar-H), 2.44 – 2.20 (m, 12H, $-CH_2$), 2.07 (qui, *J* = 14.1, 7.2 Hz, 4H, $-CH_2$), 2.01 (s, 12H, Ac-$CH_3$), 0.83 (t, *J* = 7.5 Hz, 12H, $-CH_3$), 0.10 (t, *J* = 7.2 Hz, 12H, $-CH_3$); $^{13}$C NMR (151 MHz, $d_6$-DMSO, 25 °C) δ 168.56, 147.53, 145.23, 143.50, 142.24, 138.13, 135.80, 129.87, 129.18, 123.45, 123.11, 112.41, 23.81, 19.58, 18.84, 15.55, 15.35; UV/Vis (Chloroform): λ max (log ε) = 439 (5.23), 561 (4.14), 600 (4.12); HRMS (MALDI) m/z calc. for $C_{68}H_{72}N_8O_4Ni$ $[M]^+$: 1122.5030, found 1122.5043; IR (ATR): ṽ = 3402; 2959; 2925; 1687; 1580; 1513; 1438; 1366; 1296; 1258; 1232; 1163; 1134; 1104; 1046; 1021; 995.1; 961.3; 889.1; 848.4; 798.2; 758.6; 730.1; 654.8.

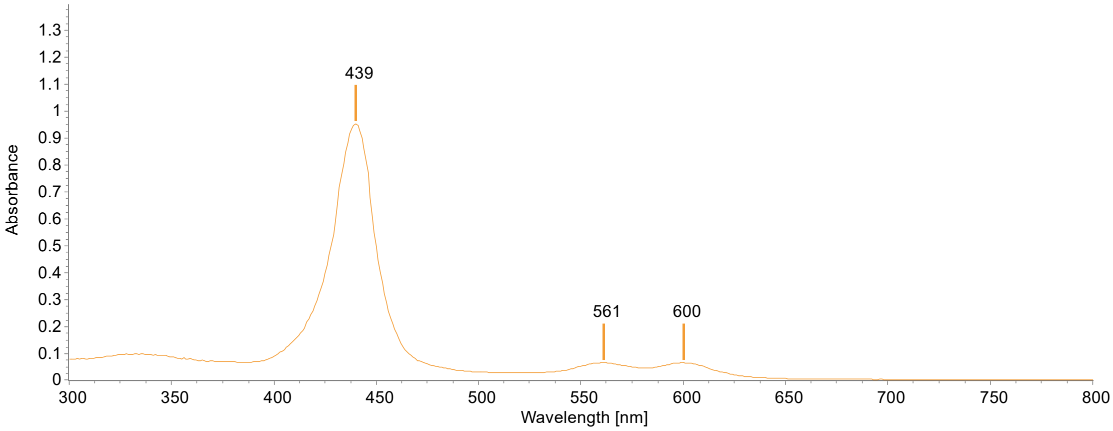


**Figure S36.** UV-vis spectrum of *αβαβ-**2*** in $CHCl_3$ [5.66 µM].

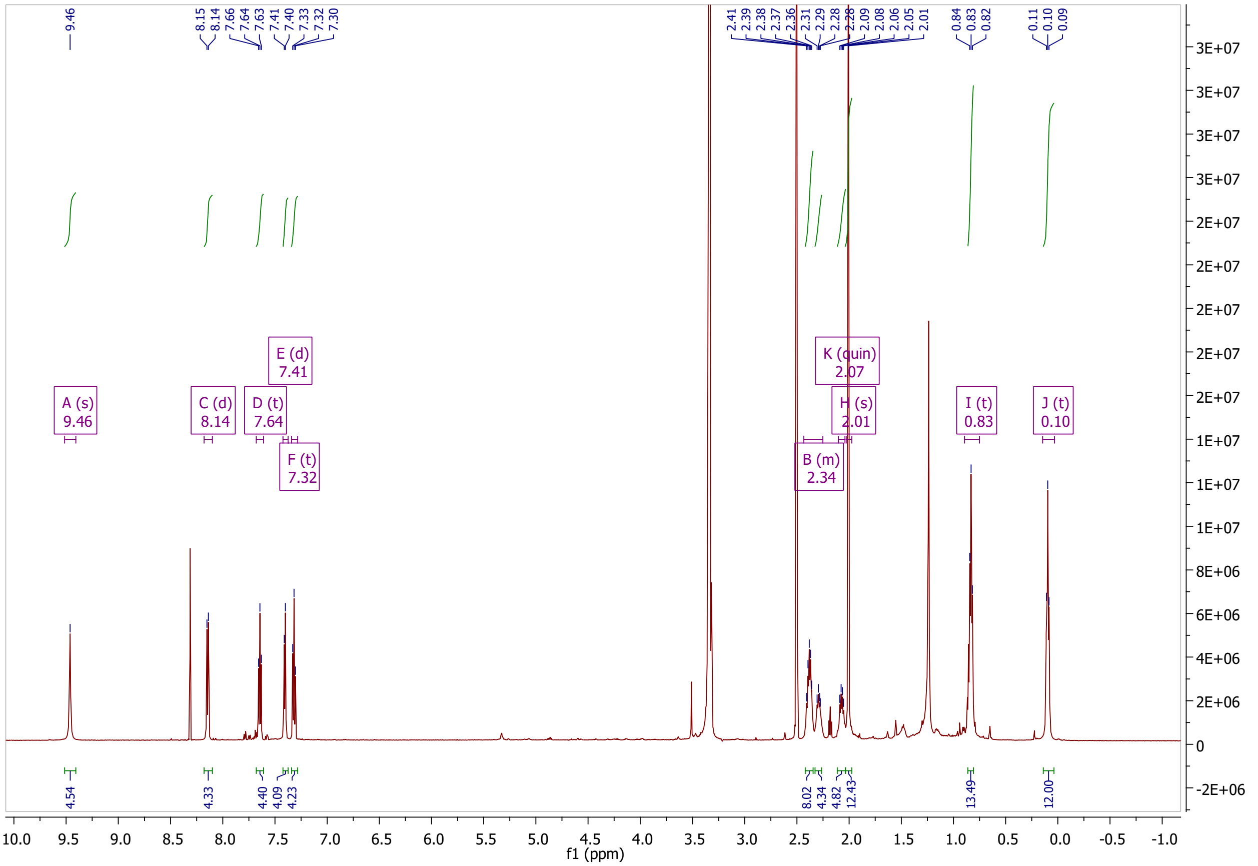


**Figure S37.** $^1$H NMR spectrum of *αβαβ-**2*** (600 MHz, $d_6$-DMSO, 25°C).

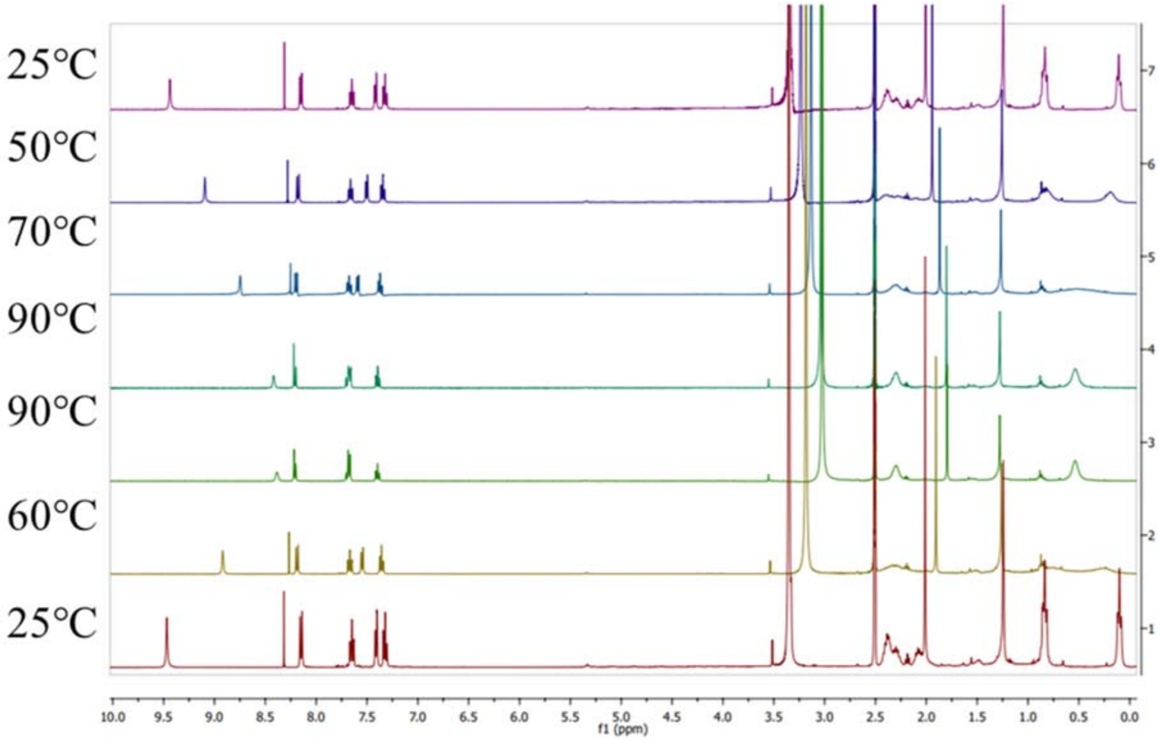


**Figure S38.** $^1$H NMR VT spectra of *αβαβ-**2*** (400 MHz, $d_6$-DMSO).

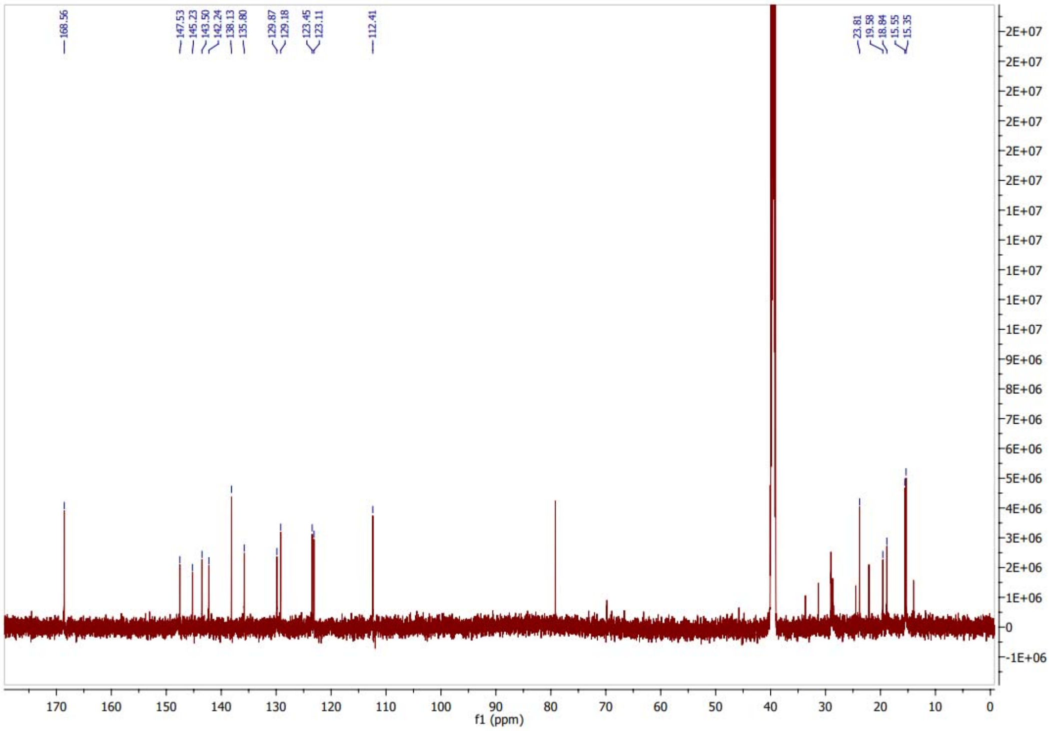


**Figure S39.** $^{13}$C NMR spectrum of *αβαβ-**2*** (151 MHz, *$d_6$*-DMSO, 25°C).

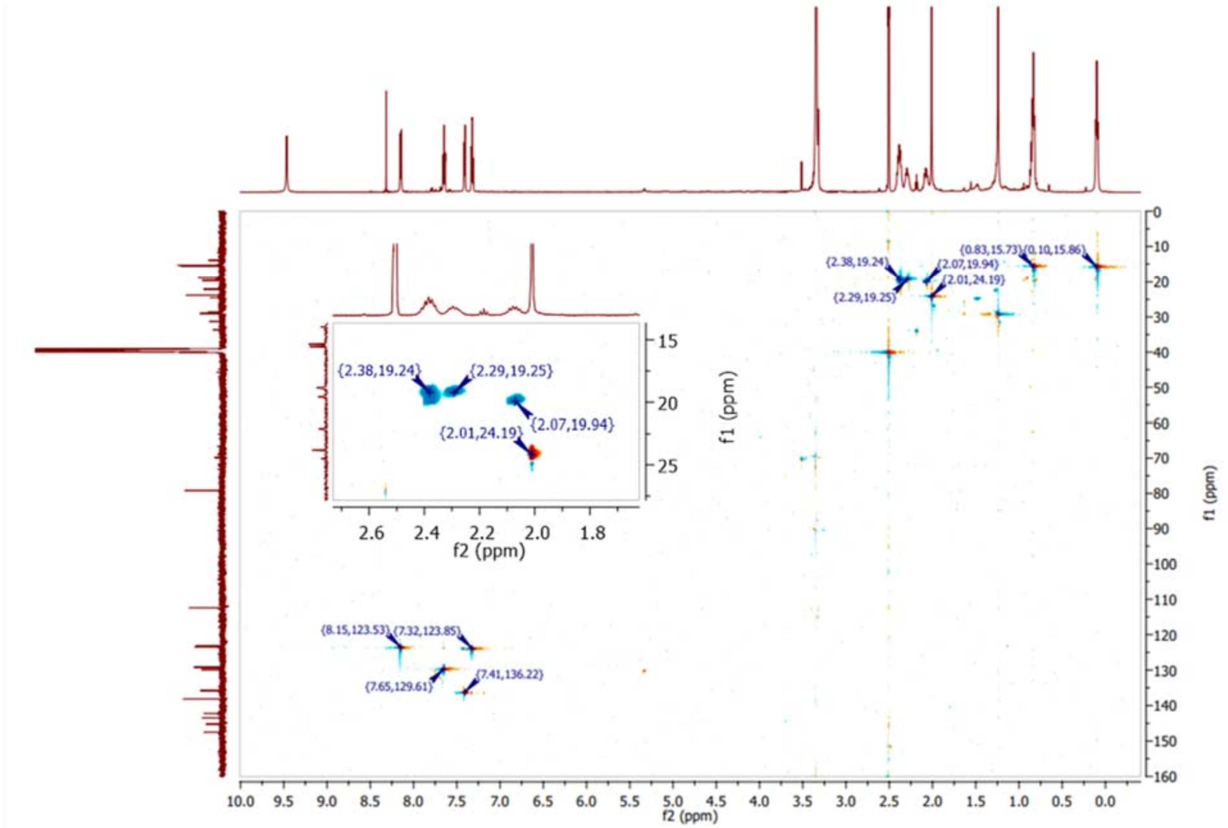


**Figure S40.** $^1$H–$^{13}$C HSQC spectrum of *αβαβ-**2*** with expansion of areas of interest (*$d_6$*-DMSO, 25°C).

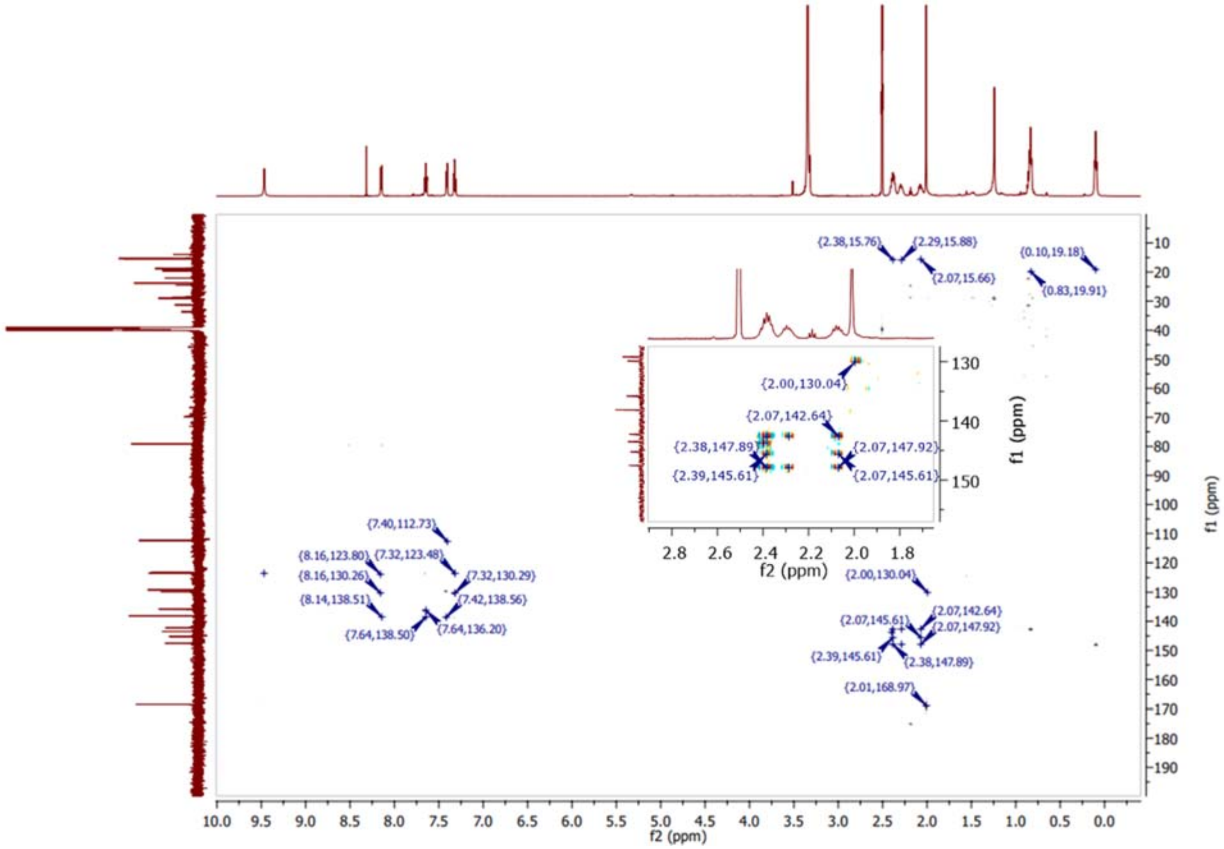


**Figure S41.** $^1H–^{13}C$ HMBC spectrum of *αβαβ-**2*** with expansion of areas of interest ($d_6$-DMSO, 25°C).

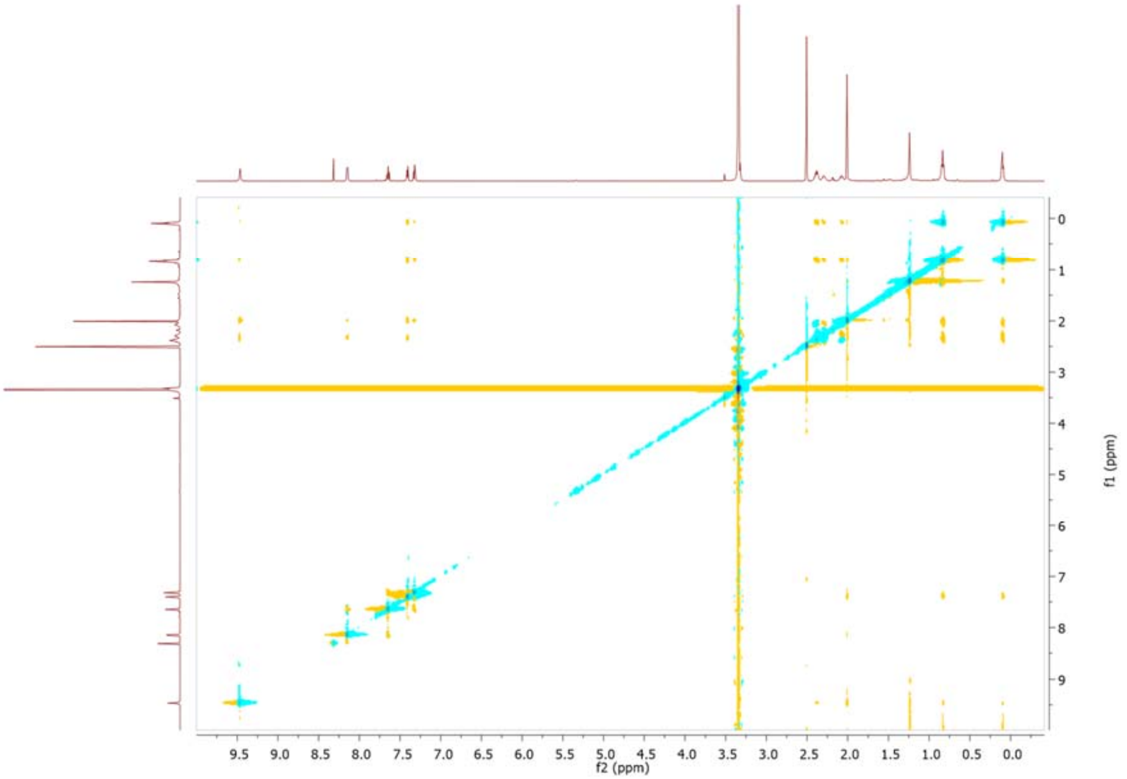


**Figure S42.** $^1H–^1H$ TOCSY spectrum of *αβαβ-**2*** ($d_6$-DMSO, 25°C).

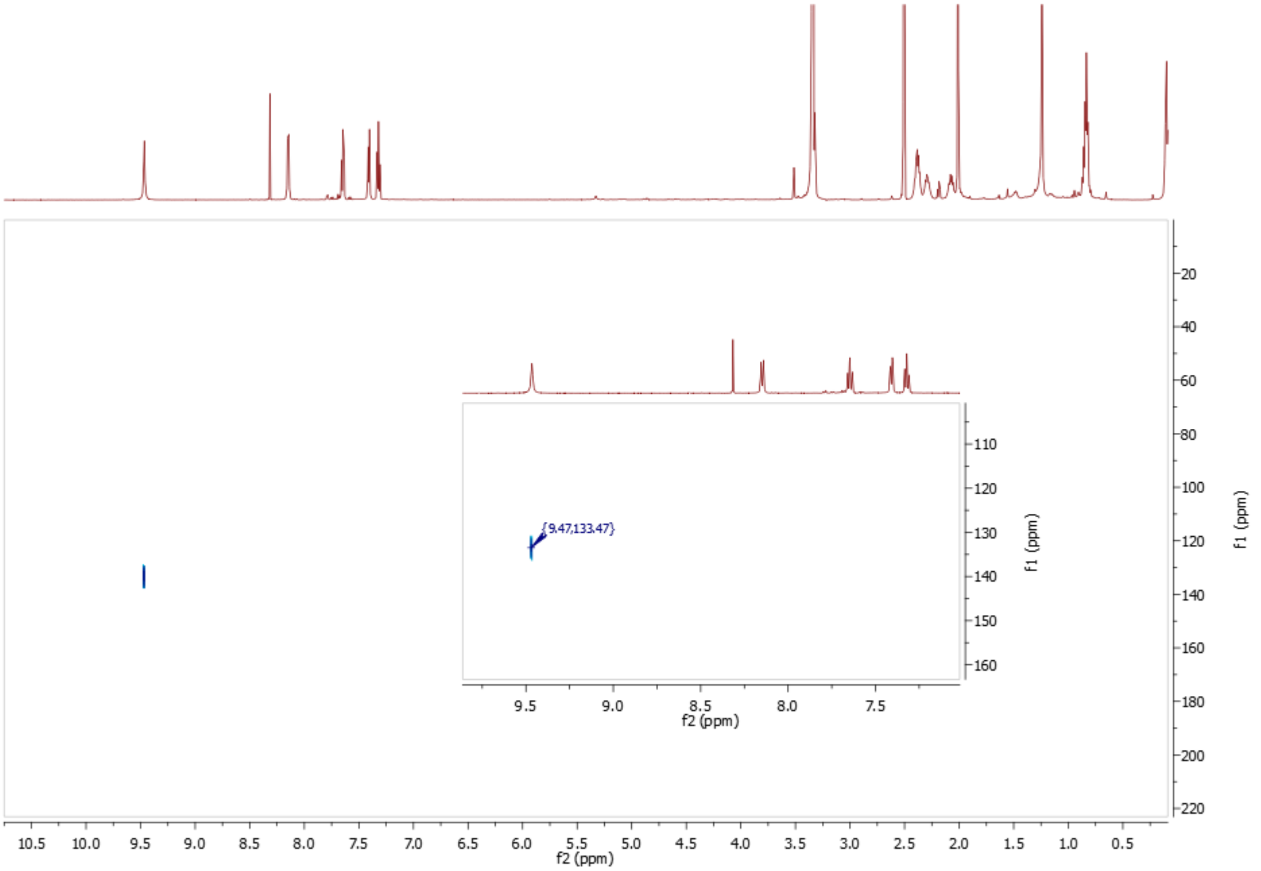


**Figure S43.** $^1H$–$^{15}N$ HSQC spectrum of *αβαβ*-**2** with expansion of areas of interest (*d*$_6$-DMSO, 25°C).

**Elemental Composition Report** **Page 1**

**Single Mass Analysis**
Tolerance = 50.0 PPM / DBE: min = -1.5, max = 400.0
Element prediction: Off
Number of isotope peaks used for i-FIT = 5

Monoisotopic Mass, Odd and Even Electron Ions
68 formula(e) evaluated with 1 results within limits (up to 10 closest results for each mass)
Elements Used:
C: 0-68 H: 0-72 N: 0-8 O: 0-4 Ni: 0-1
Karolis Norvaisa (MSe), SM002
Q-TOF20210616GH003 57 (1.321) AM (Cen,8, 80.00, Ht,10000.0,1570.68,0.70); Sm (SG, 2x3.00); Sb (15,10.00 ); Cm (13:68) TOF MS LD+ 1.47e+003

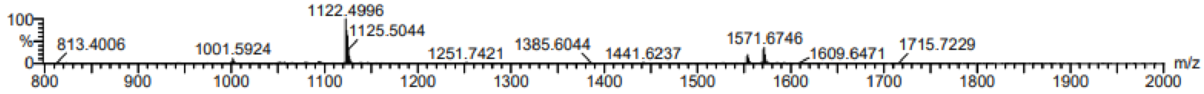


| Mass | Calc. Mass | mDa | PPM | DBE | i-FIT | i-FIT (Norm) | Formula |
|---|---|---|---|---|---|---|---|
| Minimum: | | | | -1.5 | | | |
| Maximum: | | 5.0 | 50.0 | 400.0 | | | |
| 1122.4996 | 1122.5030 | -3.4 | -3.0 | 37.5 | 63.3 | 0.0 | C68 H72 N8 O4 Ni |

**Figure S44.** HRMS (MALDI) of *αβαβ*-**2**.

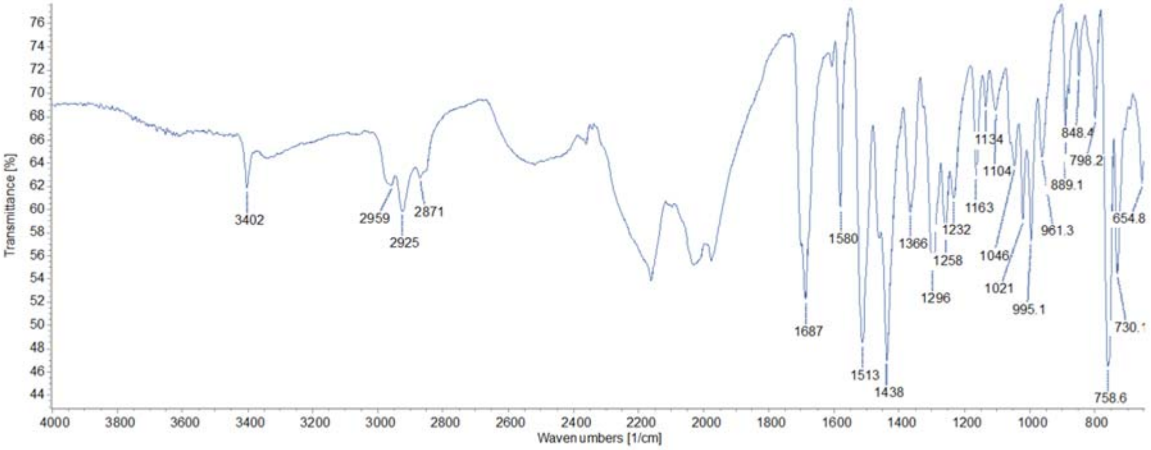


**Figure S45.** FTIR spectrum of *αβαβ*-***2***.

*Synthesis and characterization of $\alpha_2\beta_2$-**2***

**[$\alpha_2\beta_2$-5,10,15,20-Tetrakis(2-N-acetamidephenyl)-2,3,7,8,12,13,17,18-octaethylporphyrinato]nickel(II) [$\alpha_2\beta_2$-*2*]**

In 50mL round bottom flask, ($\alpha_2\beta_2$-[5,10,15,20-Tetrakis(2-aminophenyl)-2,3,7,8,12,13,17,18-octaethylporphyrinato]nickel(II)) (31.0mg, 32.43µmol, 1 eq.) was dissolved in chloroform (5mL). Acetyl chloride (276µL, 3.89mmol, 120 eq.) was added in 10 eq. additions followed by15 eq. additions of *N,N*-diisopropylethylamine (1.08mL, 5.84mmol, 180 eq.). Reaction was monitored by TLC (Dichloromethane: ethyl acetate 10:1 v/v) and was stirred at room temperature for 18 hours. Upon completion of the reaction, solvent was removed under reduced pressure, residual purple solid was dissolved in dichloromethane and transferred for column chromatography ($SiO_2$, dichloromethane: ethyl acetate 5:1 v/v). The first dark red band was collected and recrystallized in acetonitrile, giving a purple crystalline solid [29.7mg, 26.42µmol, 81%]. M.p > 300 °C. $R_f$ = 0.43 ($SiO_2$, dichloromethane: ethyl acetate 10:1 v/v); $^1$H NMR (600 MHz, $d_6$-DMSO, 25°C) δ 8.54 (s, 2H, Ar-H), 8.44 (s, 4H, N-H), 8.12 (s, 2H), 7.93 (s, 2H, Ar-H), 7.70 (t, J = 7.6 Hz, 4H, Ar-H), 7.50 (s, 2H, Ar-H), 7.40 (d, J = 44.1 Hz, 4H, Ar-H), 2.49 – 1.87 (m, 16H, -$CH_2$), 1.85 (s, 12H, Ac-$CH_3$), 0.71 – 0.21 (m, 24H, -$CH_3$); $^1$H NMR (400 MHz, $d_6$-DMSO, 80°C) δ 8.38 (d, *J* = 7.5 Hz, 4H, Ar-H), 8.08 (s, 4H, N-H), 7.71 (t, *J* = 7.8 Hz, 8H, Ar-H), 7.39 (t, *J* = 7.4 Hz, 4H, Ar-H), 2.31 (s, 16H, -$CH_2$), 1.77 (s, 12H, Ac-$CH_3$), 0.55 (t, *J* = 7.3 Hz, 24H, -$CH_3$); $^{13}$C NMR (151 MHz, $d_6$-DMSO, 25°C) δ 169.26, 145.83, 139.26, 135.99, 130.04, 124.05, 24.96, 19.09, 16.54; UV/Vis (Chloroform): λ max (log ε) = 438(5.30), 558 (4.12), 598 (4.15); HRMS (MALDI) m/z calc. for $C_{68}H_{72}N_8O_4Ni$ $[M]^+$: 1122.5030, found 1122.5043; IR (ATR): ṽ = 3403; 3353; 2964; 2928; 2871; 1687; 1622; 1579; 1513; 1437; 1367; 1294; 1258; 1228; 1163; 1135; 1102; 1051; 1021; 994.6; 962.6; 889.1; 848.6; 798.5; 757.6; 730.1; 653.5.

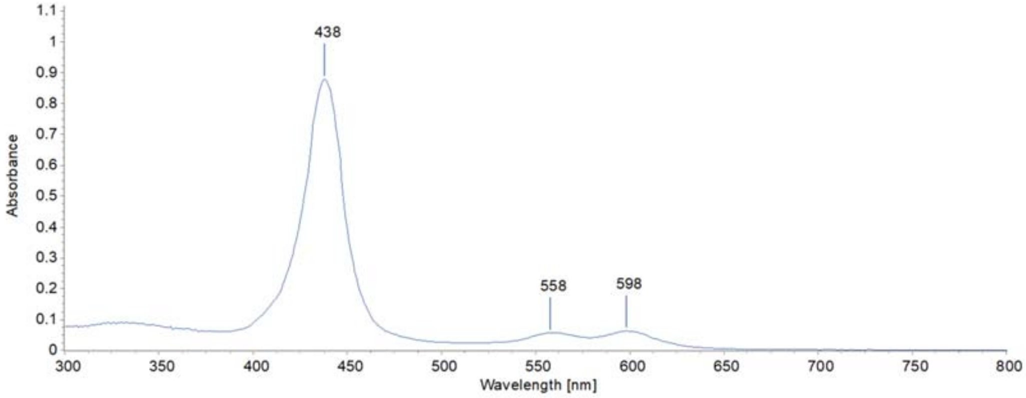


**Figure S46.** UV-vis spectrum of $\alpha_2\beta_2$-***2*** in $CHCl_3$ [4.97µM].

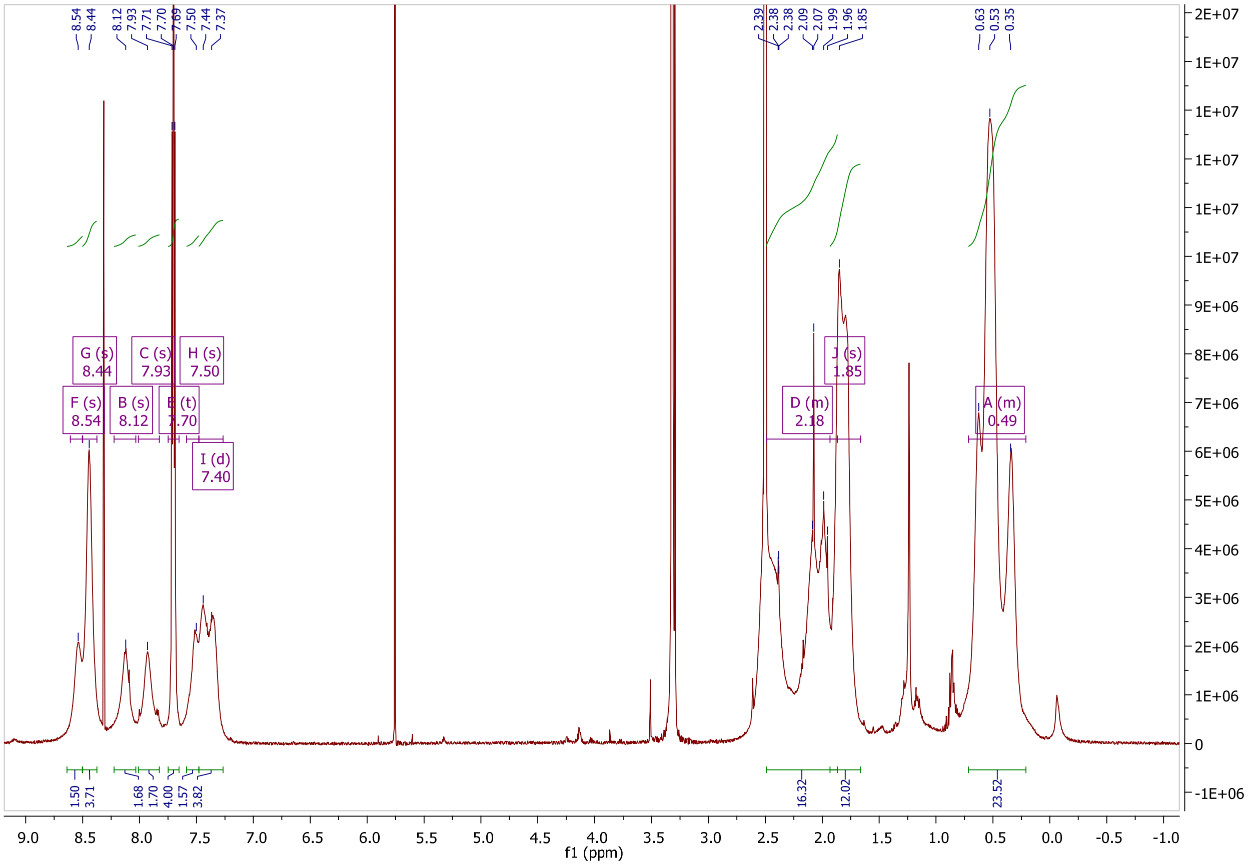


**Figure S47.** $^1$H NMR spectrum of *α*$_2$*β*$_2$-***2*** (600 MHz, *d*$_6$-DMSO, 25°C).

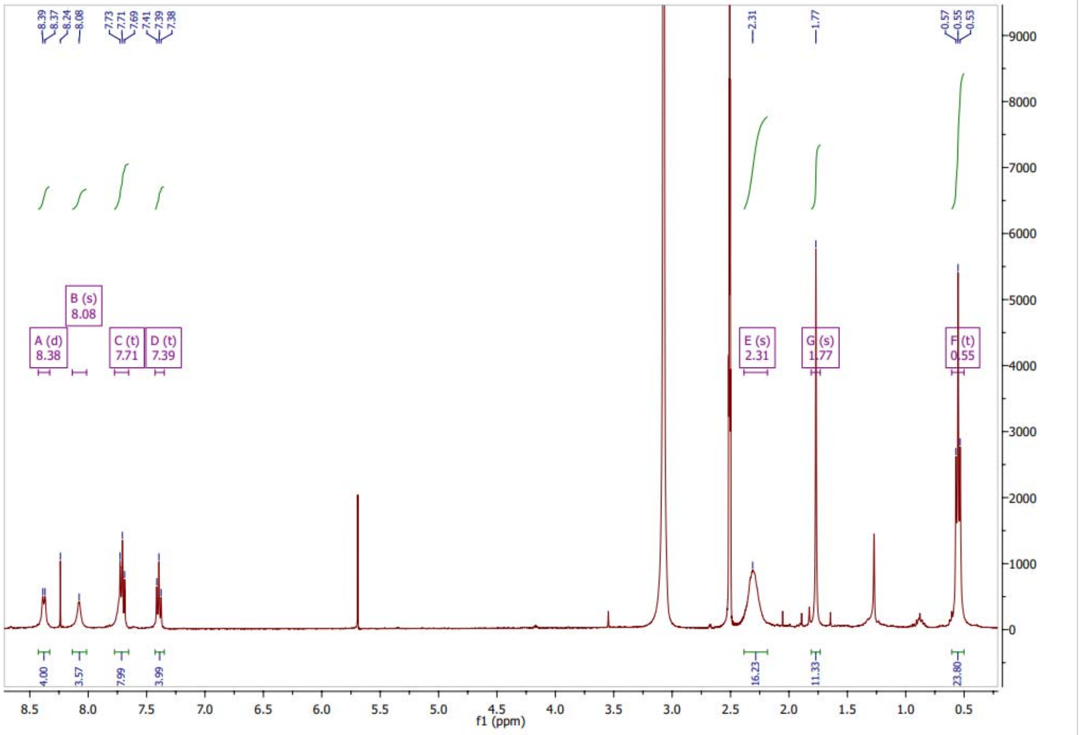


**Figure S48.** $^1$H NMR spectrum of *α*$_2$*β*$_2$-***2*** (400 MHz, *d*$_6$-DMSO, 80°C).

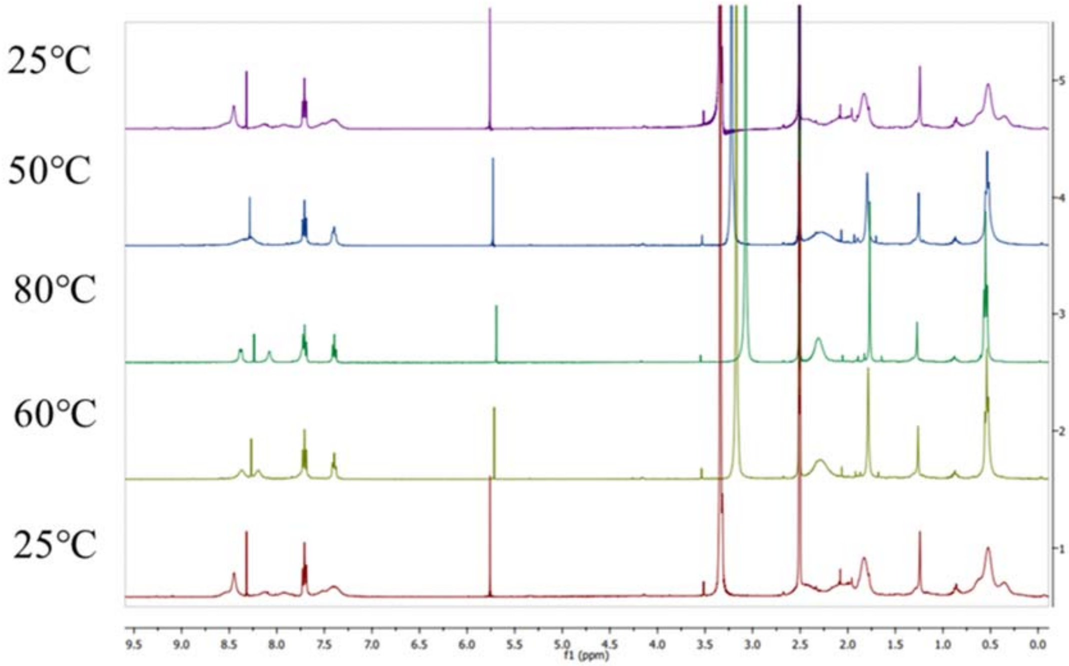


**Figure S49.** $^{1}$H NMR VT NMR spectra of $\alpha_2\beta_2$-***2*** (400 MHz, $d_6$-DMSO).

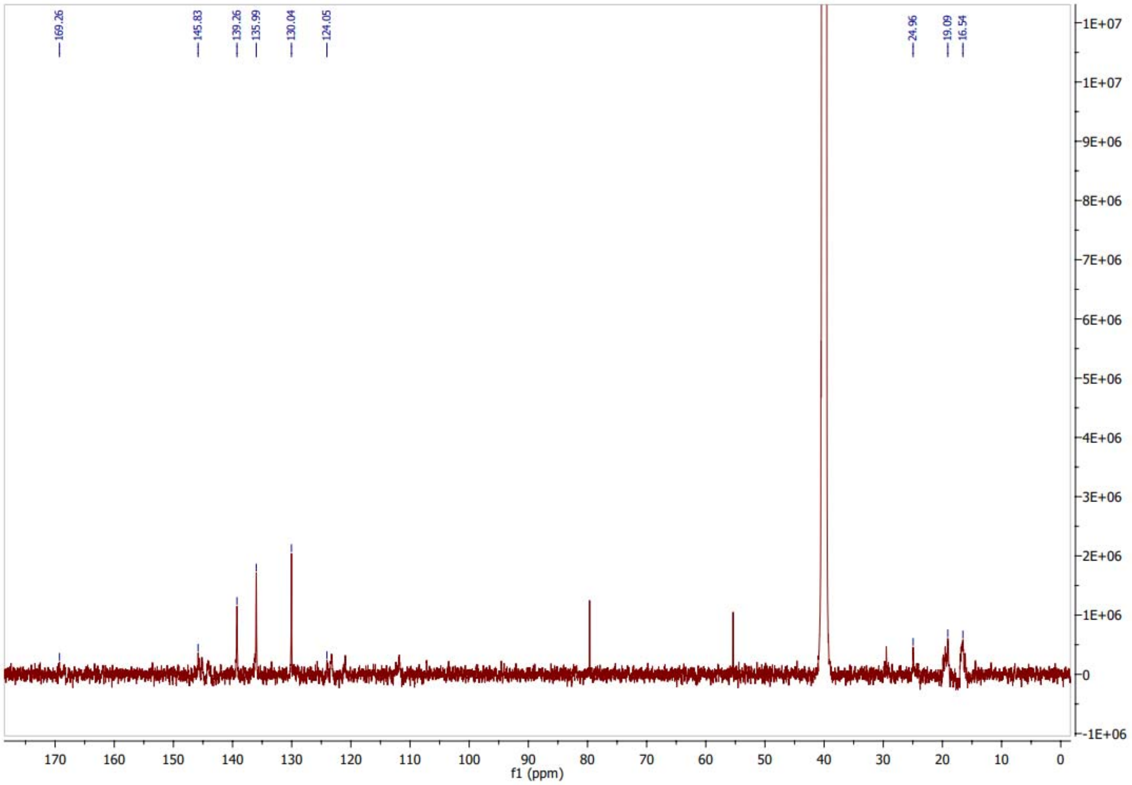


**Figure S50.** $^{13}$C NMR spectrum of $\alpha_2\beta_2$-***2*** (151 MHz, $d_6$-DMSO, 25°C).

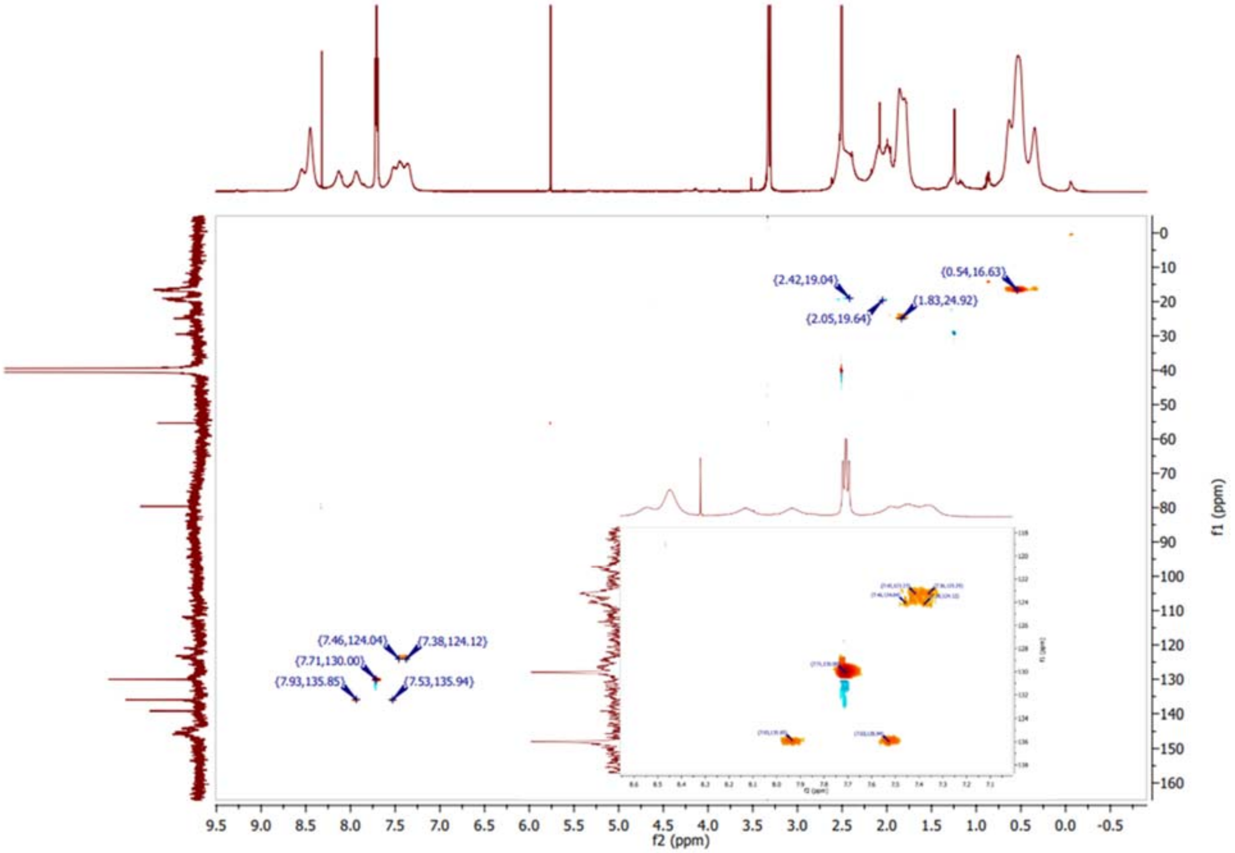


**Figure S51.** $^{1}H$–$^{13}C$ HSQC spectrum of $\alpha_2\beta_2$-***2*** with expansion of areas of interest ($d_6$-DMSO, 25°C).

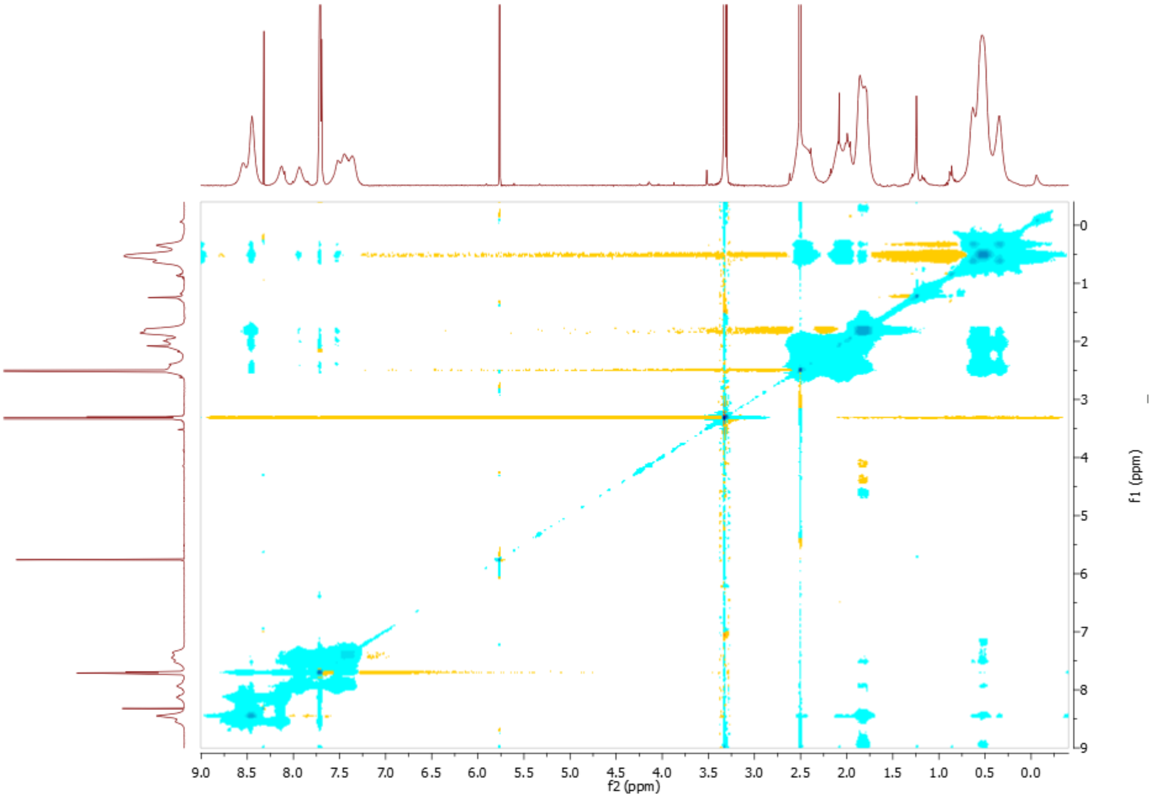


**Figure S52.** $^{1}H$–$^{1}H$ ROESY spectrum of $\alpha_2\beta_2$-***2*** ($d_6$-DMSO, 25°C).

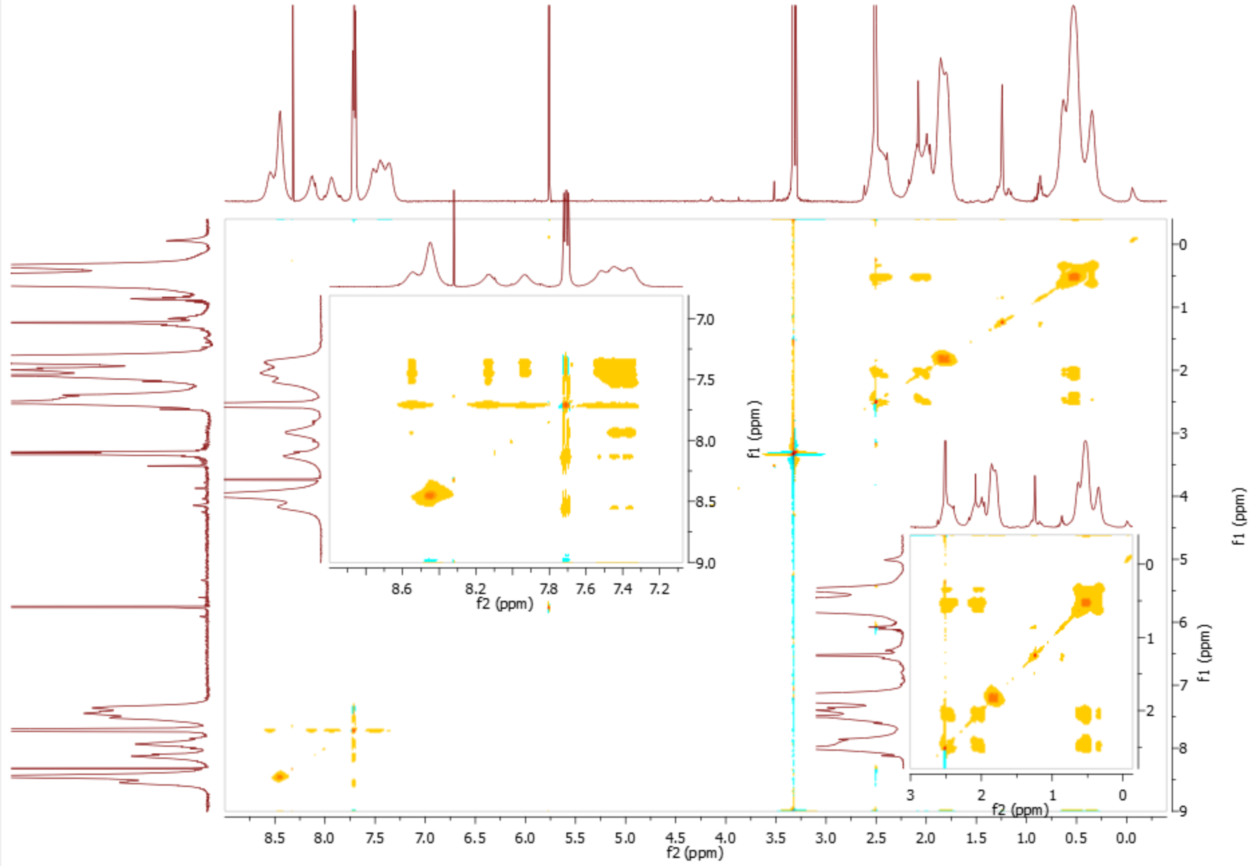


**Figure S53.** $^1H$–$^1H$ TOCSY spectrum of *α*$_2$*β*$_2$-***2*** with expansion of areas of interest (*d*$_6$-DMSO, 25°C).

**Elemental Composition Report** **Page 1**

**Single Mass Analysis**
Tolerance = 50.0 PPM / DBE: min = -1.5, max = 400.0
Element prediction: Off
Number of isotope peaks used for i-FIT = 5

Monoisotopic Mass, Odd and Even Electron Ions
68 formula(e) evaluated with 1 results within limits (up to 10 closest results for each mass)
Elements Used:
C: 0-68 H: 0-72 N: 0-8 O: 0-4 Ni: 0-1
Karolis Norvaisa (MSe), SM003
Q-TOF20210616GH004 44 (1.124) AM (Cen,8, 80.00, Ht,10000.0,1570.68,0.70); Sm (SG, 2x3.00); Sb (15,10.00 ); Cm (10:56) TOF MS LD+ 2.04e+003

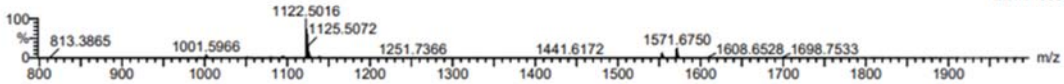


| | | | | | | | |
|---|---|---|---|---|---|---|---|
| Minimum: | | | | -1.5 | | | |
| Maximum: | | 5.0 | 50.0 | 400.0 | | | |
| Mass | Calc. Mass | mDa | PPM | DBE | i-FIT | i-FIT (Norm) | Formula |
| 1122.5016 | 1122.5030 | -1.4 | -1.2 | 37.5 | 58.7 | 0.0 | C68 H72 N8 O4 Ni |

**Figure S54.** HRMS (MALDI) of *α₂β₂*-**2**.

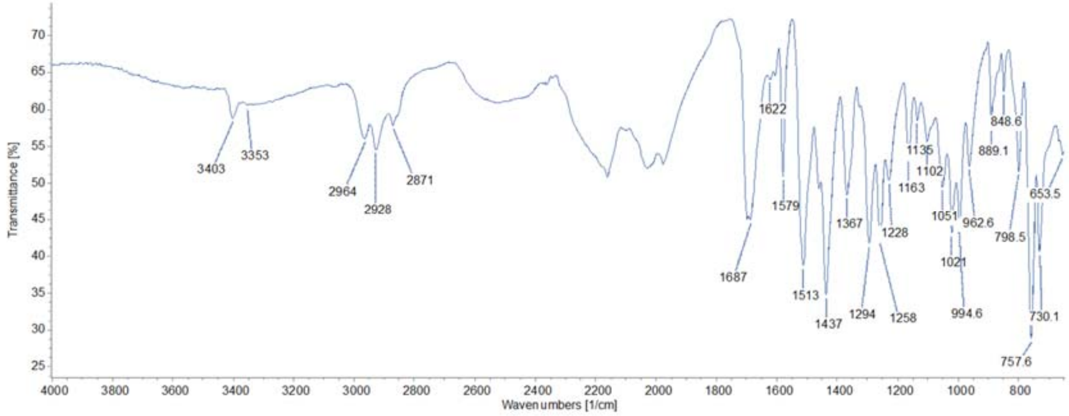


**Figure S55.** FTIR spectrum of *α₂β₂*-**2**.

*Synthesis and characterization of α₄-**2***

**[$\alpha_4$-5,10,15,20-Tetrakis(2-N-acetamidephenyl)-2,3,7,8,12,13,17,18-octaethylporphyrinato]nickel(II) [$\alpha_4$-*2*]**

In 50mL round bottom flask, ($\alpha_4$-[5,10,15,20-Tetrakis(2-aminophenyl)-2,3,7,8,12,13,17,18-octaethylporphyrinato]nickel(II)) (30.6mg, 32.01µmol, 1 eq.) was dissolved in chloroform (5mL). Acetyl chloride (230µL, 3.20mmol, 100 eq.) was added in 10 eq. additions following 15 eq. additions of *N,N*-diisopropylethylamine (890µL, 4.8mmol, 150 eq.). Reaction was stirred at room temperature for 20hrs and monitored by TLC (Dichloromethane: ethyl acetate 2:1 v/v). Solvent was removed under reduced pressure, and the purple solid was dissolved in dichloromethane and transferred to column chromatography ($SiO_2$, dichloromethane: ethyl acetate 4:1 v/v). The third dark red band was collected before removing solvent under reduced pressure. Product was recrystallized in acetonitrile, giving a purple crystalline solid [16.9mg, 15.03µmol, 47%]. M.p >250 °C; $R_f$ = 0.31 ($SiO_2$, dichloromethane: ethyl acetate 1:1 v/v); $^1$H NMR (600 MHz, $d_6$-DMSO, 25 °C) δ 8.42 (d, *J* = 8.1 Hz, 4H, Ar-H), 8.39 (s, 4H, N-H), 7.69 (t, *J* = 7.9 Hz, 4H, Ar-H), 7.66 (d, *J* = 7.4 Hz, 4H, Ar-H), 7.38 (t, *J* = 7.5 Hz, 4H, Ar-H), 2.48 – 1.98 (m, 16H, -$CH_2$), 1.76 (s, 12H, Ac-$CH_3$), 0.53 (s, 24H, -$CH_3$); $^{13}$C NMR (151 MHz, $d_6$-DMSO, 25 °C) δ 169.08, 145.97, 139.16, 136.10, 130.08, 123.71, 121.40, 111.99, 24.67, 16.61; UV/Vis (Chloroform): λ max (log ε) = 438 (5.27), 561 (4.09), 599 (4.15); HRMS (MALDI) m/z calc. for $C_{68}H_{72}N_8O_4Ni$ $[M]^+$: 1122.5030, found 1122.5043; IR (ATR): ṽ =3396, 2971, 2930, 2872, 1697, 1580, 1514, 1437, 1369, 1293, 1256, 1226, 1163, 1135, 1104, 1051, 994.4, 962.7, 889.6, 849.4, 799.4, 756.9, 730.2, 653.4.

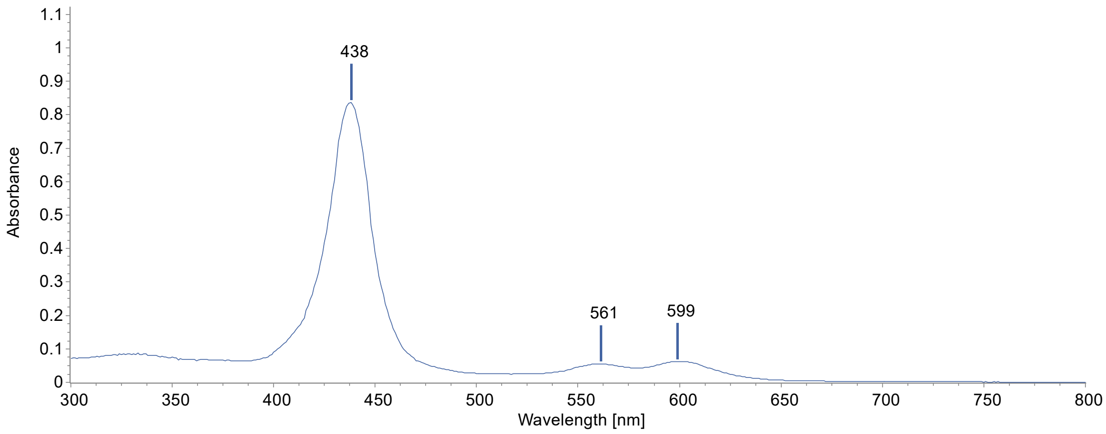


**Figure S56.** UV-vis spectrum of $\alpha_4$-***2*** in $CHCl_3$ [4.45µM].

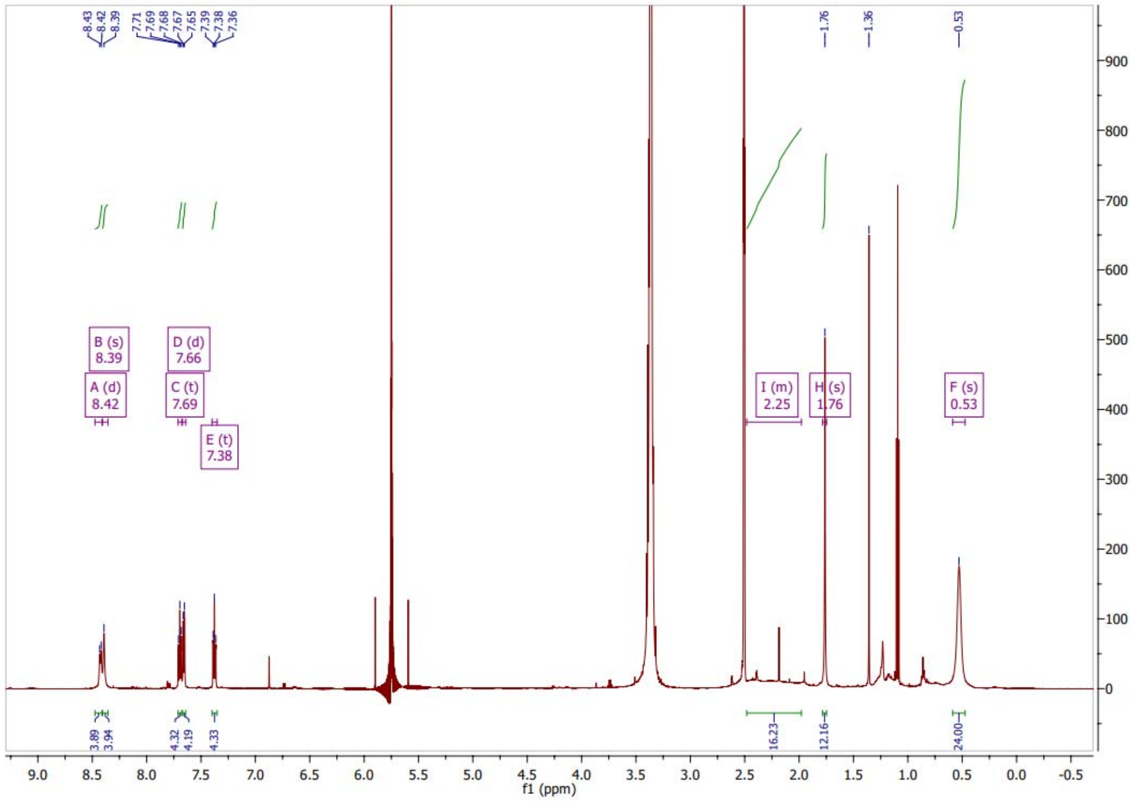


**Figure S57.** $^{1}H$ NMR spectrum of *α₄*-**2** (600 MHz, $d_6$-DMSO, 25°C).

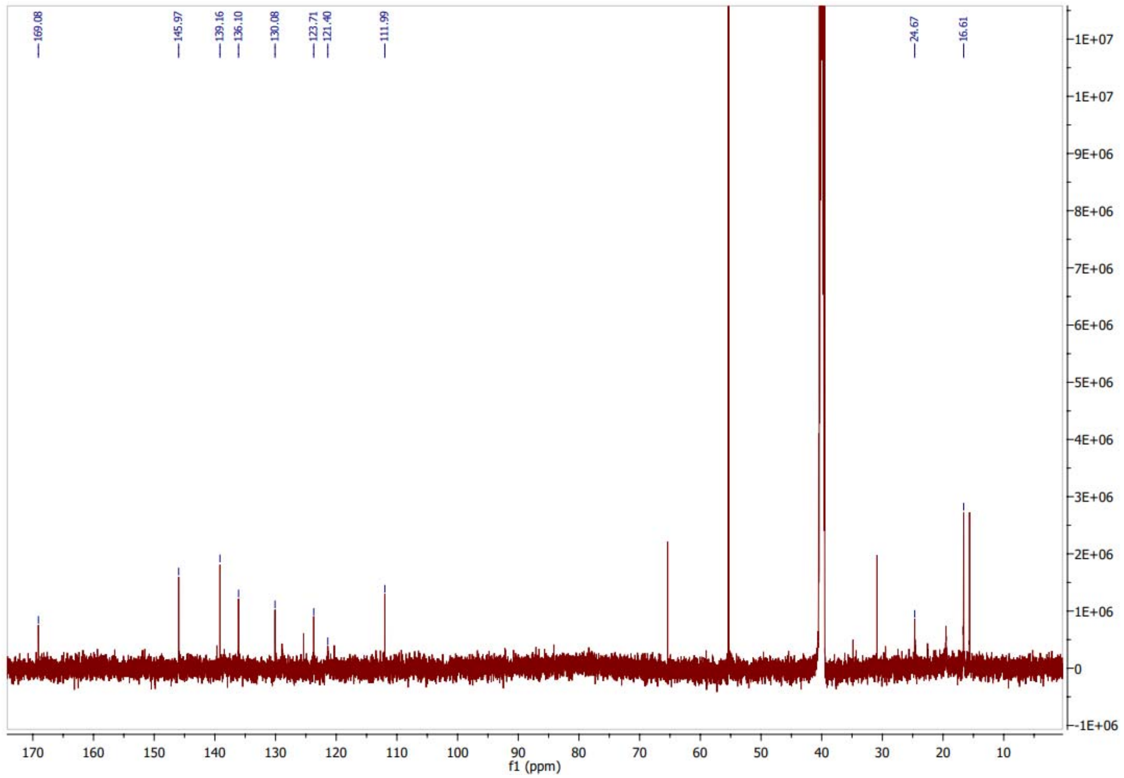


**Figure S58.** $^{13}C$ NMR spectrum of $α_4$-**2** (151 MHz, $d_6$-DMSO, 25°C).

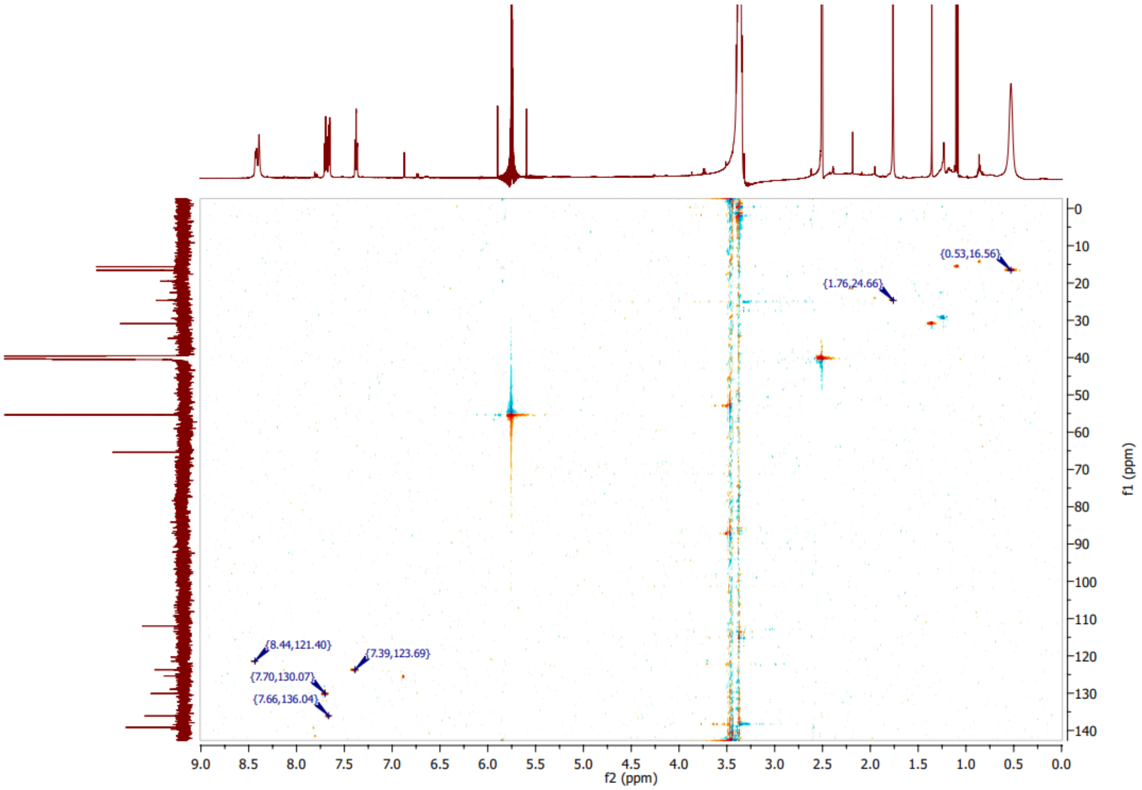


**Figure S59.** $^1H$–$^{13}C$ HSQC spectrum of $\alpha_4$-**2** ($d_6$-DMSO, 25°C).

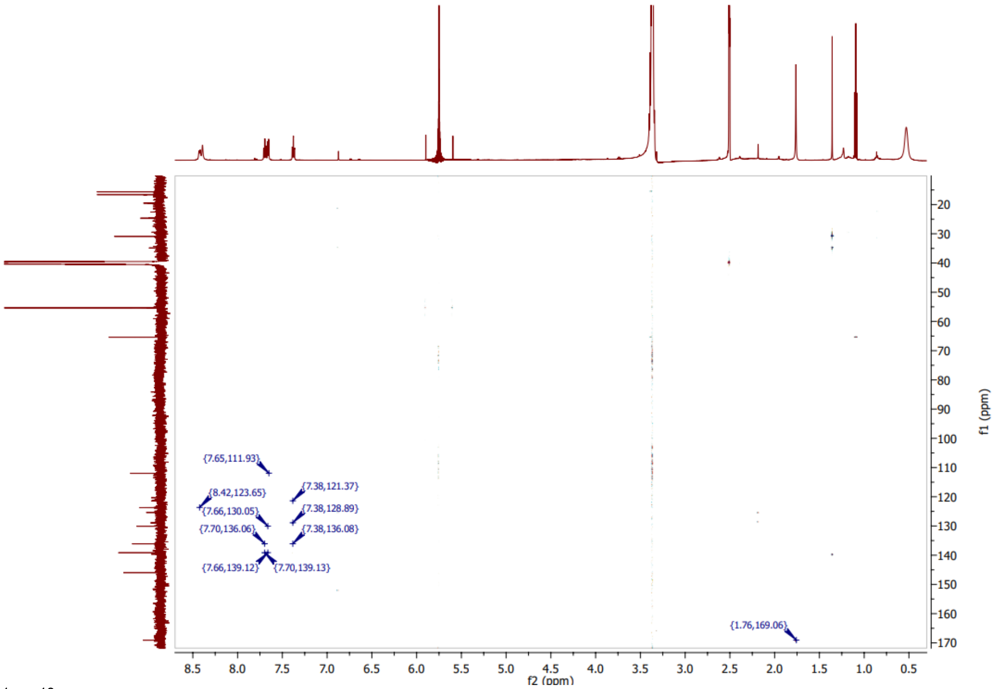


**Figure S60.** $^1H$–$^{13}C$ HMBC spectrum of $\alpha_4$-**2** ($d_6$-DMSO, 25°C).

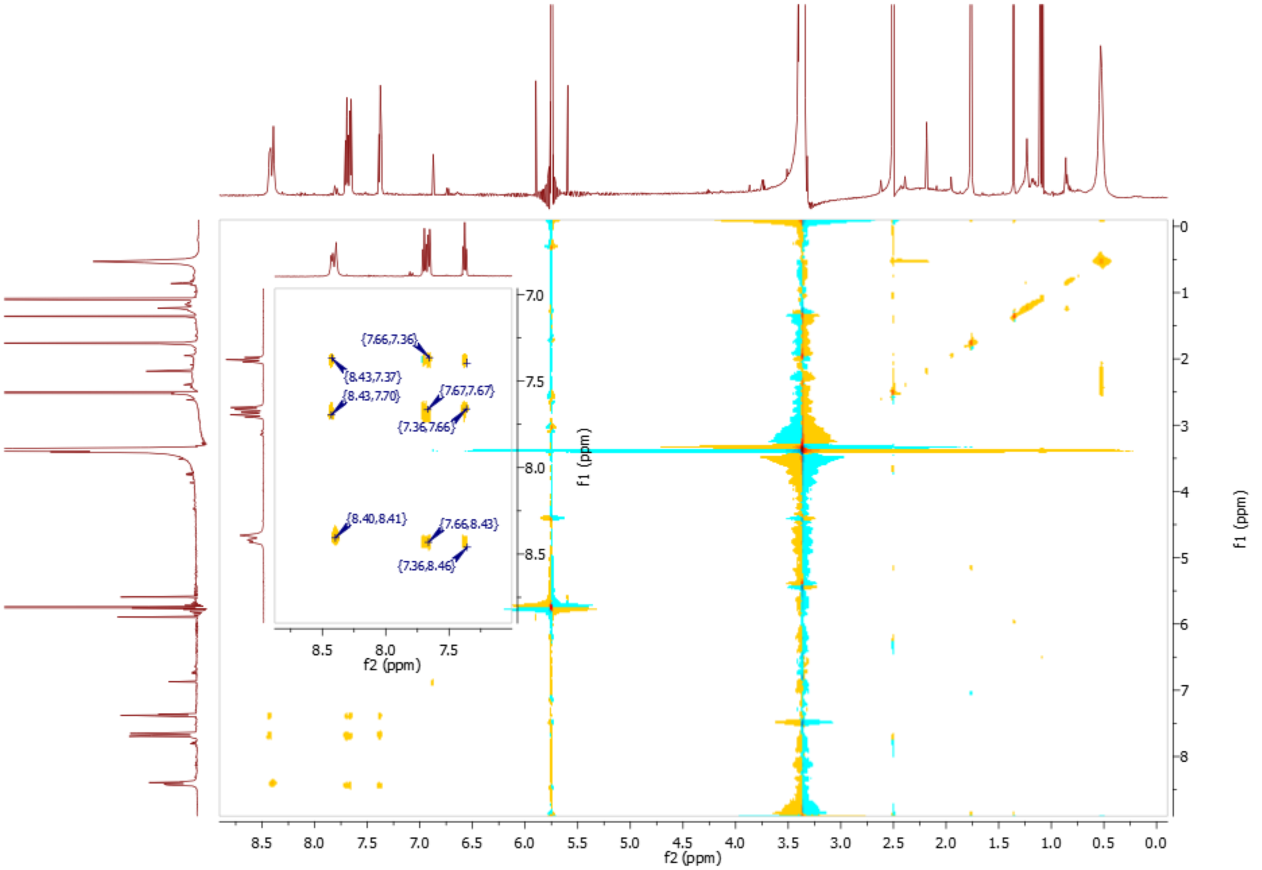


**Figure S61.** $^{1}H$–$^{1}H$ TOCSY spectrum of *α$_4$*-***2*** with expansion of areas of interest (*d$_6$*-DMSO, 25°C).

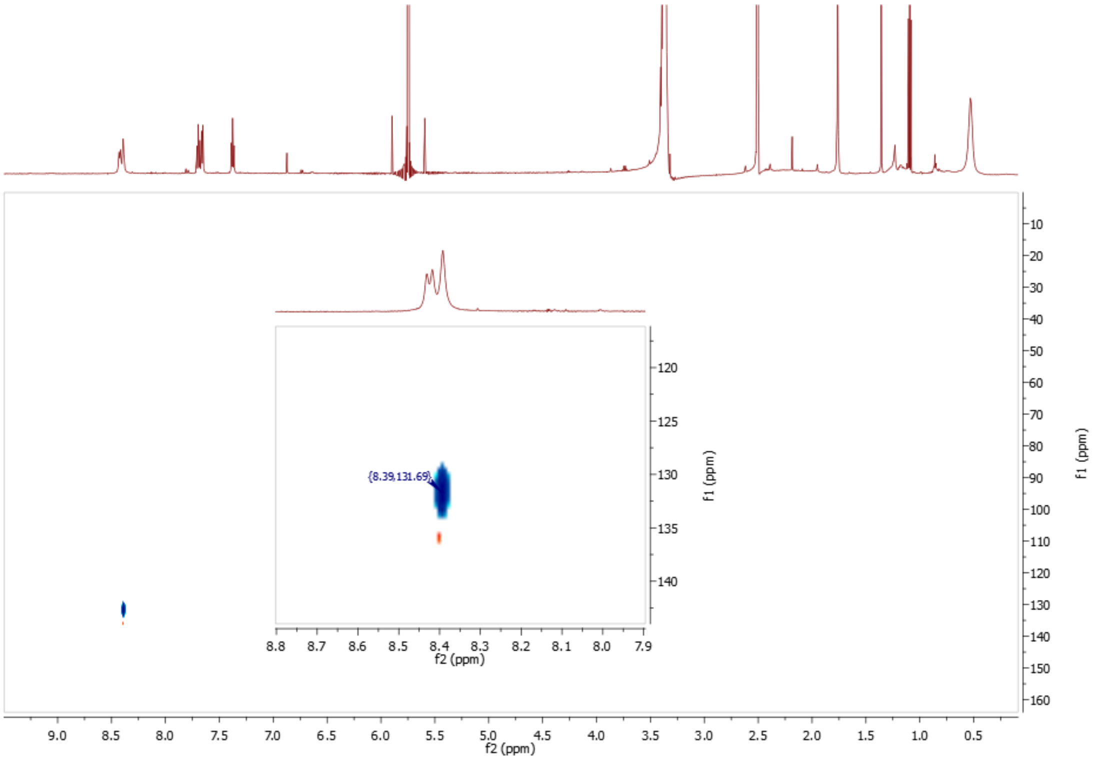


**Figure S62.** $^{1}H$–$^{15}N$ HSQC spectrum of *α$_4$*-***2*** with expansion of areas of interest (*d$_6$*-DMSO, 25°C).

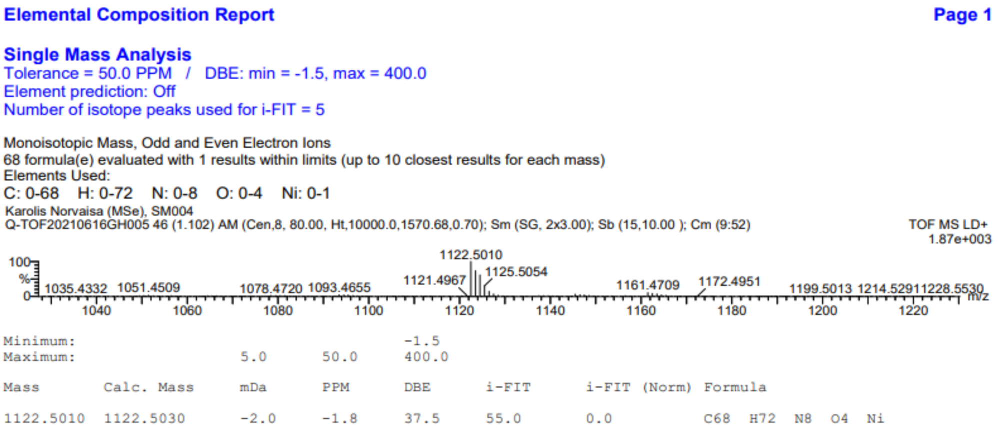


**Figure S63.** HRMS (MALDI) of *α₄*-***2***.

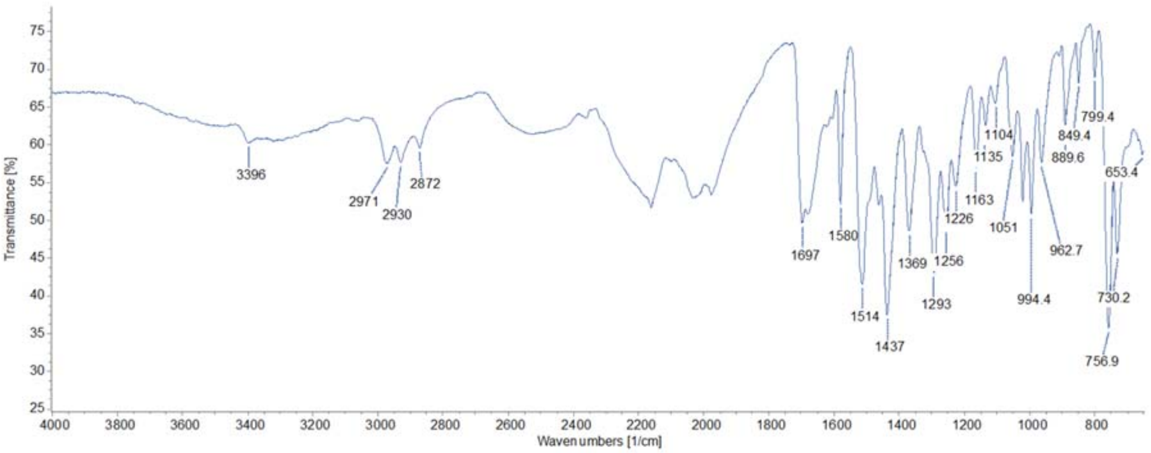


**Figure S64.** FTIR spectrum of *α₄*-***2***.

*Synthesis and characterization of αβαβ-**3***

**[*αβαβ*-5,10,15,20-Tetrakis(2-pivalamidophenyl)-2,3,7,8,12,13,17,18-octaethylporphyrinato]nickel(II) [*αβαβ-3*]**

In a 50mL dried Schlenk tube containing $α_3β$-5,10,15,20-tetrakis(2-aminophenyl)-2,3,7,8,12,13,17,18-octaethylporphyrinato]nickel(II) (10.4mg, 10.88μmol, 1 eq.) was charged with 3mL dichlorobenzene and purged with argon. Separately, pivaloyl chloride (187μL, 1.52mmol, 140 eq.) and *N,N*-diisopropylethylamine (460μL, 2.68mmol, 246 eq.) were dissolved in dichlorobenzene (0.7mL) and was added in small portions respectively. Reaction was stirred at room temperature for 3hrs before heating to 120°C for 15hrs. Reaction was monitored by TLC (Dichloromethane: ethyl acetate 10:1 v/v) and mass spectrometry (Figure S22). Upon cooling down the reaction mixture, column chromatography, ($SiO_2$, Hexane) was used to remove dichlorobenzene, then (Dichloromethane: Ethyl acetate 10:1 v/v) was used to collect dark red band. Product was recrystallised in acetonitrile, giving a purple crystalline solid [7.4mg, 5.73μmol, 53%] M.p 250 °C. $R_f$ = 0.51 ($SiO_2$, dichloromethane: ethyl acetate 10:1 v/v). $^1$H NMR (600 MHz, $CDCl_3$, 25°C) δ 8.78 (d, *J* = 8.3 Hz, 4H, Ar-H), 7.93 (s, 4H, N-H), 7.74 (t, *J* = 7.9 Hz, 4H, Ar-H), 7.63 (d, *J* = 7.4 Hz, 4H, Ar-H), 7.32 (t, *J* = 7.5 Hz, 4H, Ar-H), 2.38 (s, 16H, $-CH_2$), 0.79 (s, 36H, t-Bu), 0.57 (s, 24H, $-CH_3$). $^{13}$C NMR (151 MHz, $CDCl_3$, 25°C) δ 176.04, 146.58, 138.89, 134.20, 130.58, 127.78, 122.79, 119.70, 111.34, 39.67, 27.27, 19.34, 16.47. UV/Vis (Chloroform): λ max (log ε) = 437 (5.05), 558 (4.21), 596 (4.24); HRMS (MALDI) m/z calc. for $C_{80}H_{96}N_8O_4Ni$ $[M]^+$:1290.6908, found 1290.6890; IR (ATR): ṽ =3419, 2960, 2926, 2869, 1731, 1690, 1606, 1581, 1513, 1437, 1396, 1366, 1302, 1259, 1214, 1110, 1052, 1021, 993, 962.3, 918.6, 888.5, 848.9, 798.0, 760.5, 729.2.

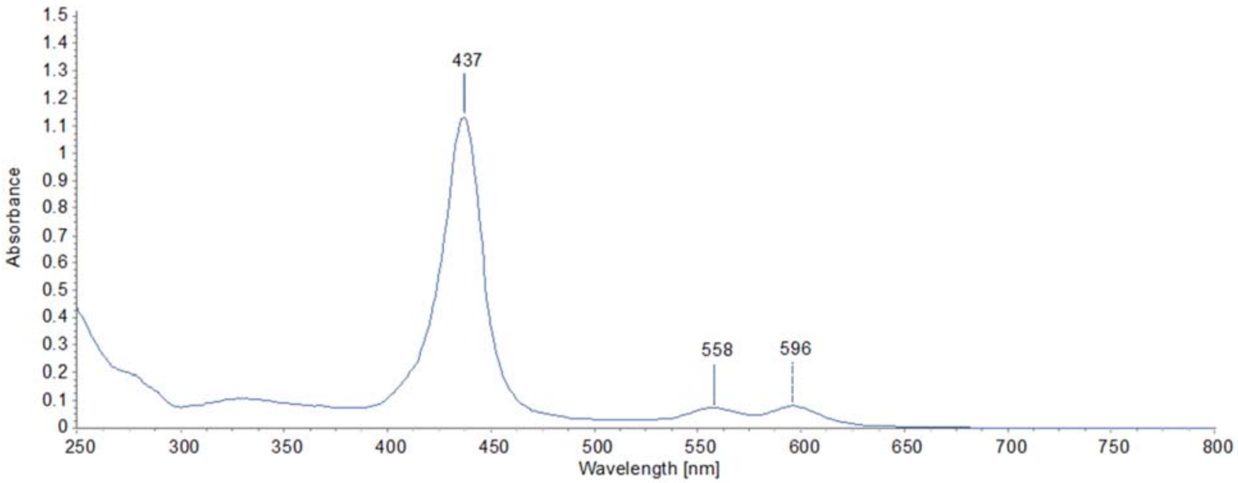


**Figure S65.** UV-vis spectrum of *αβαβ*-**3** in $CHCl_3$ [10.17μL].

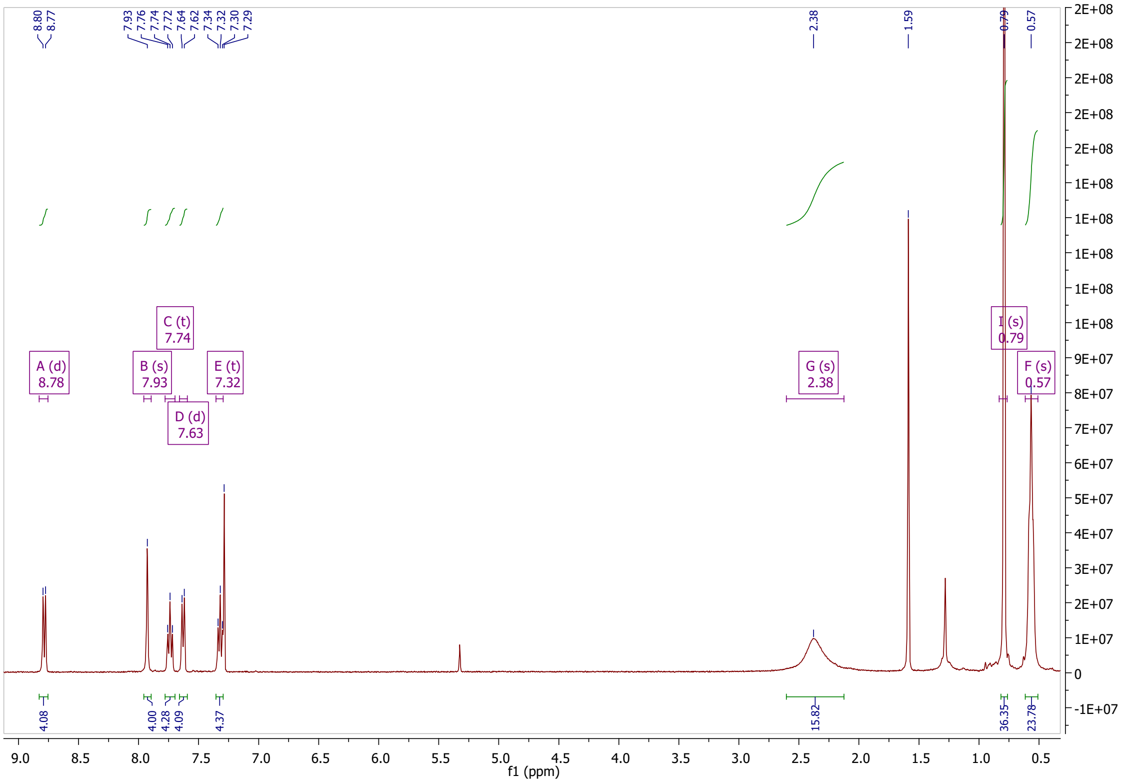


**Figure S66.** $^{1}H$ NMR spectrum of *αβαβ*-**3** (600 MHz, $CDCl_3$, 25°C).

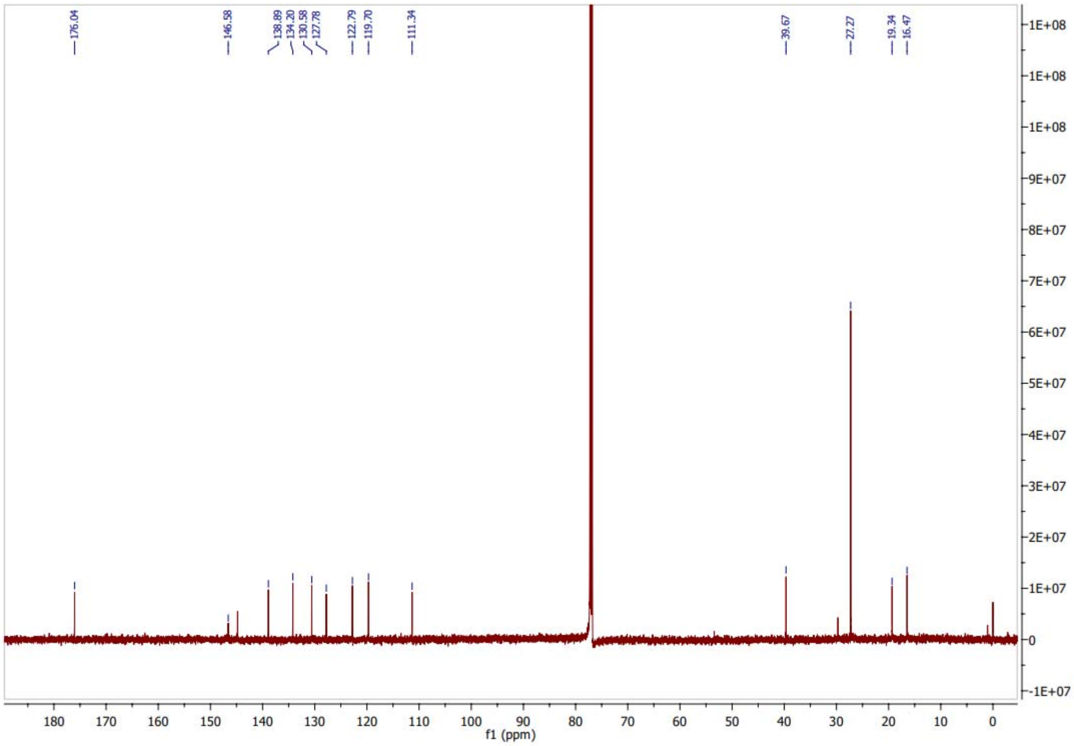


**Figure S67.** $^{13}C$ NMR spectrum of *αβαβ*-**3** (151 MHz, $CDCl_3$, 25°C).

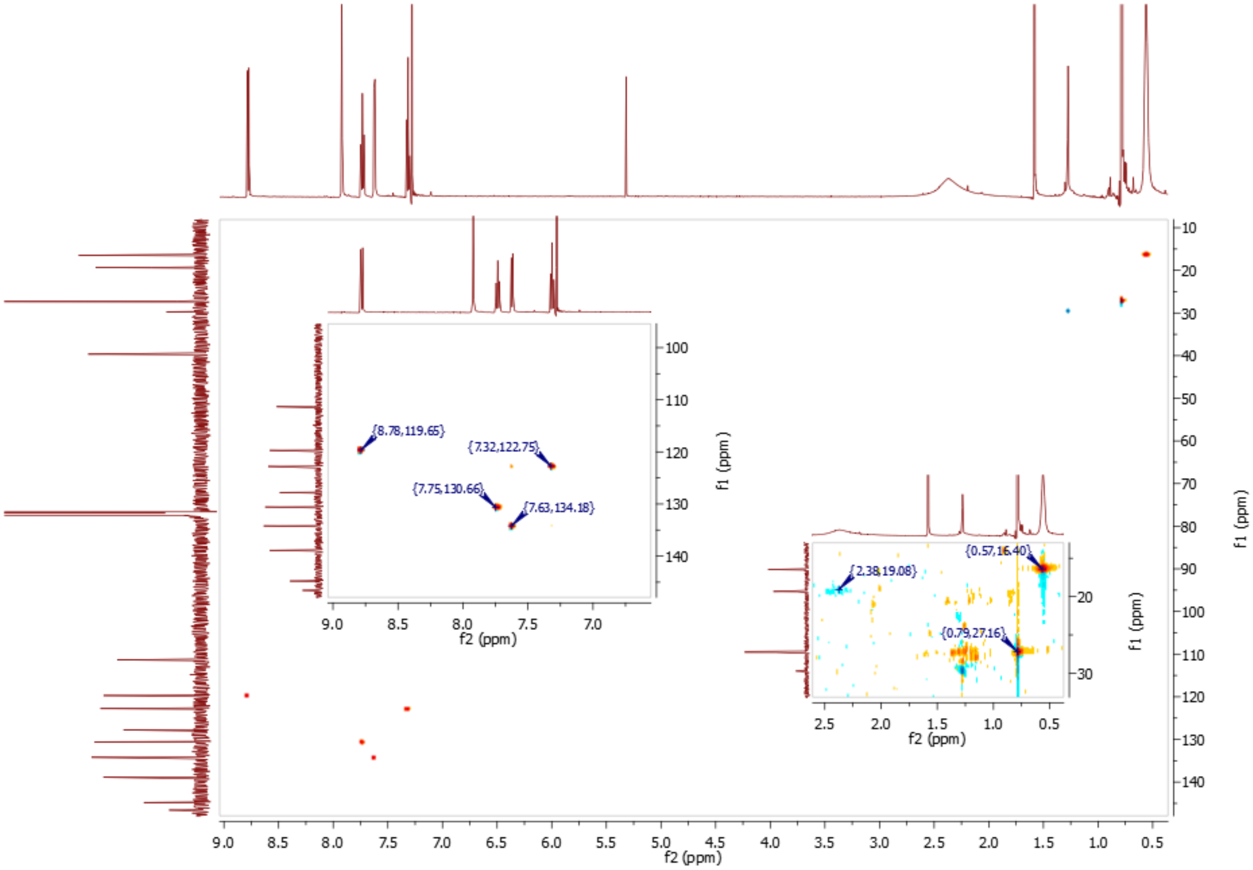

**Figure S68.** $^{1}H$–$^{13}C$ HSQC spectrum of *αβαβ*-**3** with expansion of areas of interest ($CDCl_3$, 25°C).

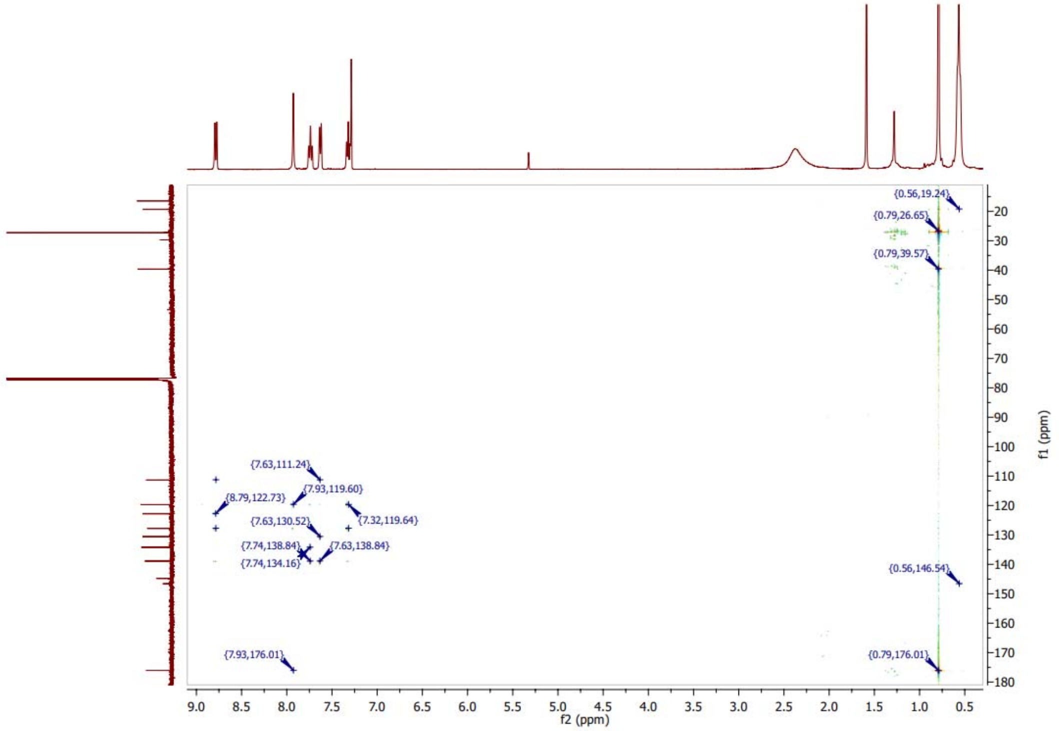

**Figure S69.** $^{1}H$–$^{13}C$ HMBC spectrum of *αβαβ*-**3** ($CDCl_3$, 25°C)

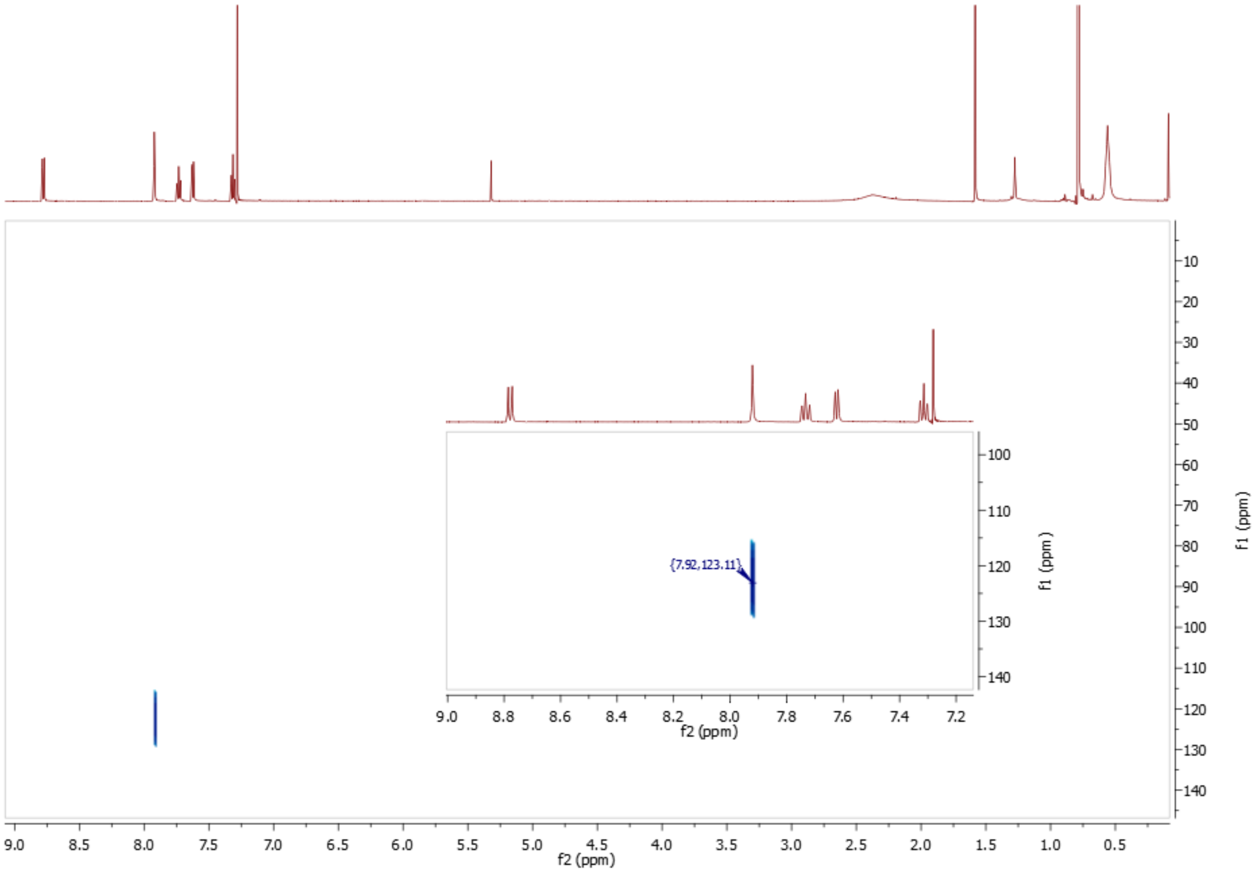


**Figure S70.** $^{1}H$–$^{15}N$ HSQC spectrum of *αβαβ*-**3** with expansion of areas of interest ($CDCl_3$, 25°C)

**Elemental Composition Report** **Page 1**

**Single Mass Analysis**
Tolerance = 50.0 PPM / DBE: min = -1.5, max = 400.0
Element prediction: Off
Number of isotope peaks used for i-FIT = 5

Monoisotopic Mass, Odd and Even Electron Ions
68 formula(e) evaluated with 1 results within limits (up to 10 closest results for each mass)
Elements Used:
C: 0-80 H: 0-96 N: 0-8 O: 0-4 Ni: 0-1

Karolis Norvaisa (MSe), SM010
Q-TOF20210707GH002 44 (0.815) AM (Cen,8, 80.00, Ht,10000.0,1570.68,0.70); Sm (SG, 2x3.00); Sb (15,10.00 ); Cm (12:59) TOF MS LD+ 1.48e+003

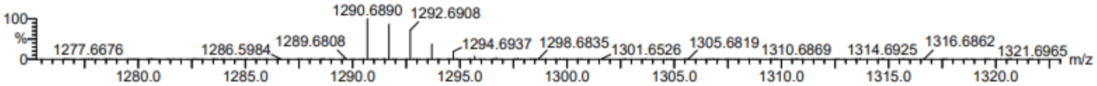


| | | | | | | | |
|---|---|---|---|---|---|---|---|
| Minimum: | | | | -1.5 | | | |
| Maximum: | | 5.0 | 50.0 | 400.0 | | | |
| Mass | Calc. Mass | mDa | PPM | DBE | i-FIT | i-FIT (Norm) | Formula |
| 1290.6890 | 1290.6908 | -1.8 | -1.4 | 37.5 | 64.8 | 0.0 | C80 H96 N8 O4 Ni |

**Figure S71.** HRMS (MALDI) of *αβαβ*-**3**.

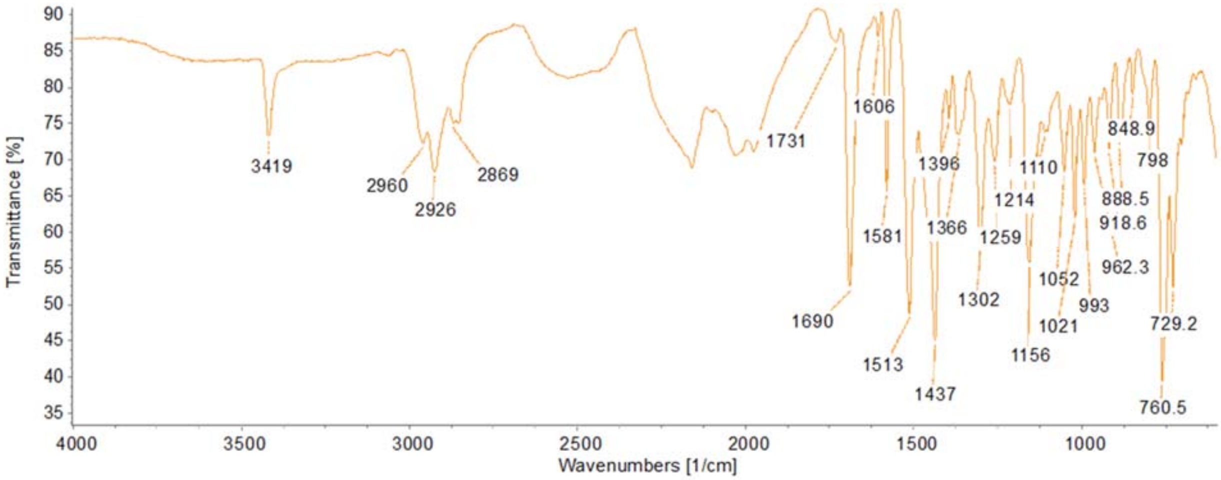


**Figure S72.** FTIR spectrum of *αβαβ*-**3**.

*Synthesis and characterization of α3β-**3***

**[*α3β-3* -5,10,15,20-Tetrakis(2-pivalamidophenyl)-2,3,7,8,12,13,17,18-octaethylporphyrinato]nickel(II) [*α3β-3*]**

A 50mL dried Schlenk tube containing α3β-5,10,15,20-tetrakis(2-aminophenyl)-2,3,7,8,12,13,17,18-octaethylporphyrinato]nickel(II) (11.6mg, 12.13μmol, 1 eq.), pivaloyl chloride (180μL, 1.46mmol, 120 eq.) and *N,N*-diisopropylethylamine (422μL, 2.43mmol, 200 eq.) in chloroform (4mL) was stirred for 1 hour. Then the temperature was raised to 66 °C and reaction mixture was stirred for additional 2 hours. The solution was cooled down to 25 °C and washed with 2x50mL $NaHCO_3$ and 1x50 mL saturated NaCl solutions. Organic layer was dried over $MgSO_4$. Upon removal of the solvent under reduced pressure the purple solid was dissolved in dichloromethane and transferred for column chromatography ($SiO_2$, dichloromethane: ethyl acetate 25:1 v/v). The second green band was collected, and solvent was removed under reduced pressure, yielding a purple solid product [8.5mg, 6.58μmol, 54%]. M.p >250 °C; Rf = 0.21 ($SiO_2$, dichloromethane: ethyl acetate 100:1 v/v); $^1$H NMR (600 MHz, $CDCl_3$, 25 °C) δ 8.69 (d, *J* = 8.3 Hz, 1H, Ar-H), 8.62 (d, *J* = 8.2 Hz, 1H, Ar-H), 8.55 (d, *J* = 8.3 Hz, 2H, Ar-H), 8.14 (d, *J* = 7.5 Hz, 2H, Ar-H), 7.97 (d, *J* = 7.5 Hz, 1H, Ar-H), 7.80 (s, 1H, N-H), 7.74 – 7.61 (m, 4H, Ar-H), 7.51 (d, *J* = 7.5 Hz, 1H, Ar-H), 7.41 (t, *J* = 8.2 Hz, 3H, Ar-H), 7.32 – 7.26 (m, 1H, Ar-H), 6.88 (s, 1H, N-H), 6.37 (s, 2H, N-H), 2.67 – 2.47 (m, 12H, -$CH_2$), 2.37 – 2.24 (m, 4H, -$CH_2$), 0.73 (t, *J* = 7.4 Hz, 6H, -$CH_3$), 0.65 (s, 9H, t-Bu), 0.61 (t, *J* = 7.4 Hz, 6H, -$CH_3$), 0.54 (t, *J* = 7.4 Hz, 12H, -$CH_3$), 0.39 (s, 9H, t-Bu), 0.12 (s, 18H, t-Bu). $^{13}$C NMR (101 MHz, $CDCl_3$, 25 °C) δ 176.52, 176.02, 175.58, 147.49, 147.10, 147.05, 146.95, 143.99, 143.71, 143.58, 143.54, 139.41, 139.23, 138.17, 135.05, 133.42, 132.46, 130.79, 130.64, 130.38, 127.12, 126.89, 123.33, 122.79, 122.62, 120.14, 119.96, 119.84, 112.06, 110.74, 39.61, 39.44, 39.23, 27.21, 26.87, 26.71, 20.02, 19.75, 19.70, 16.63, 16.60, 16.56, 16.43; $^1$H NMR (400 MHz, *d6*-DMSO, 25 °C) δ 8.50 (d, *J* = 8.3 Hz, 1H, Ar-H), 8.35 (d, *J* = 8.4 Hz, 2H, Ar-H), 8.23 (d, *J* = 7.9 Hz, 1H, Ar-H), 8.16 (d, *J* = 7.3 Hz, 2H, Ar-H), 7.98 (d, *J* = 7.0 Hz, 1H, Ar-H), 7.77 – 7.62 (m, 4H, Ar-H, 1H, N-H), 7.48 (t, *J* = 7.6 Hz, 4H, , Ar-H), 7.31 (t, *J* = 7.5 Hz, 1H, Ar-H), 7.09 (s, 1H, N-H), 6.21 (s, 2H, N-H), 2.66 – 2.49 (m, 6H, -$CH_2$), 2.46 – 2.35 (m, 5H, -$CH_2$), 2.30 – 2.13 (m, 5H, -$CH_2$), 0.64 – 0.52 (m, 12H, -$CH_3$), 0.52 (s, 9H, t-Bu), 0.48 – 0.39 (m, 12H, -$CH_3$), 0.29 (s, 9H, t-Bu), -0.03 (s, 18H, t-Bu);$^{13}$C NMR (151 MHz, *d6*-DMSO, 25 °C) δ 175.65, 175.35, 174.81, 147.30, 146.69, 143.52, 143.28, 143.14, 139.38, 139.16, 138.05, 135.33, 134.00, 132.74, 130.95, 130.70, 128.06, 126.97, 126.61, 123.83, 123.62, 123.12, 119.53, 111.83, 111.72, 110.75, 39.34, 39.06, 38.97, 27.13, 26.73, 26.55, 19.77, 19.53, 19.39, 16.67, 16.60, 16.53; UV/Vis (Chloroform): λ max (log ε) = 438 (5.37), 562 (4.13), 607 (4.30); HRMS (MALDI) m/z calc. for for $C_{80}H_{96}N_8O_4Ni$ $[M]^+$: 1290.6908, found 1290.6873; IR (ATR): ṽ = 1290.6908, found 1290.6890; IR (ATR): ṽ =3416, 2960, 2929, 2870, 1690, 1580, 1513, 1436, 1366, 1299, 1258, 1214, 1153, 1100, 1052, 1020, 993.3, 961.8, 917.4, 888.8, 851.3, 797.8, 760.6, 728.6, 647.

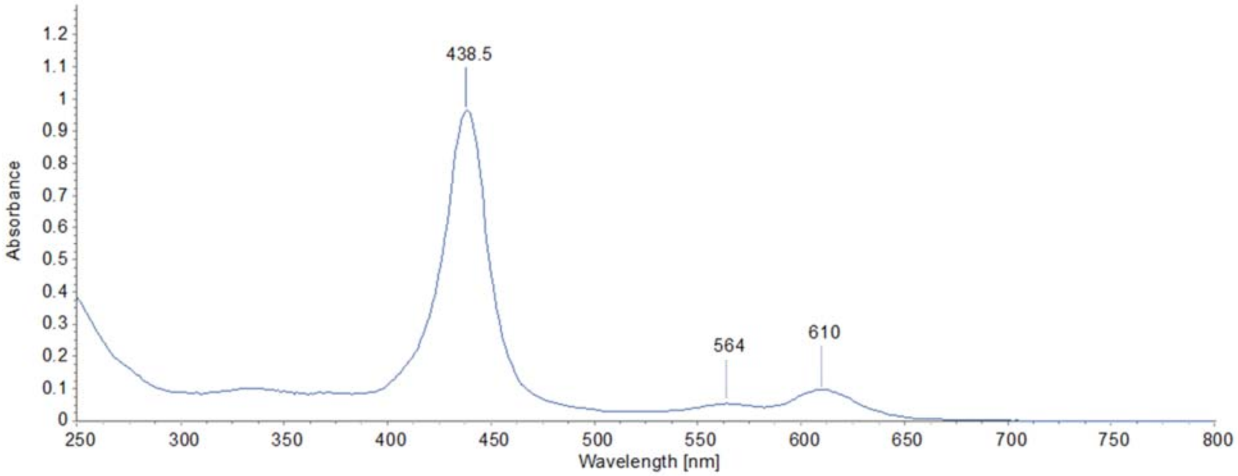


**Figure S73.** UV-vis spectrum of *α3β*-**3** in $CHCl_3$ [19.45μM].

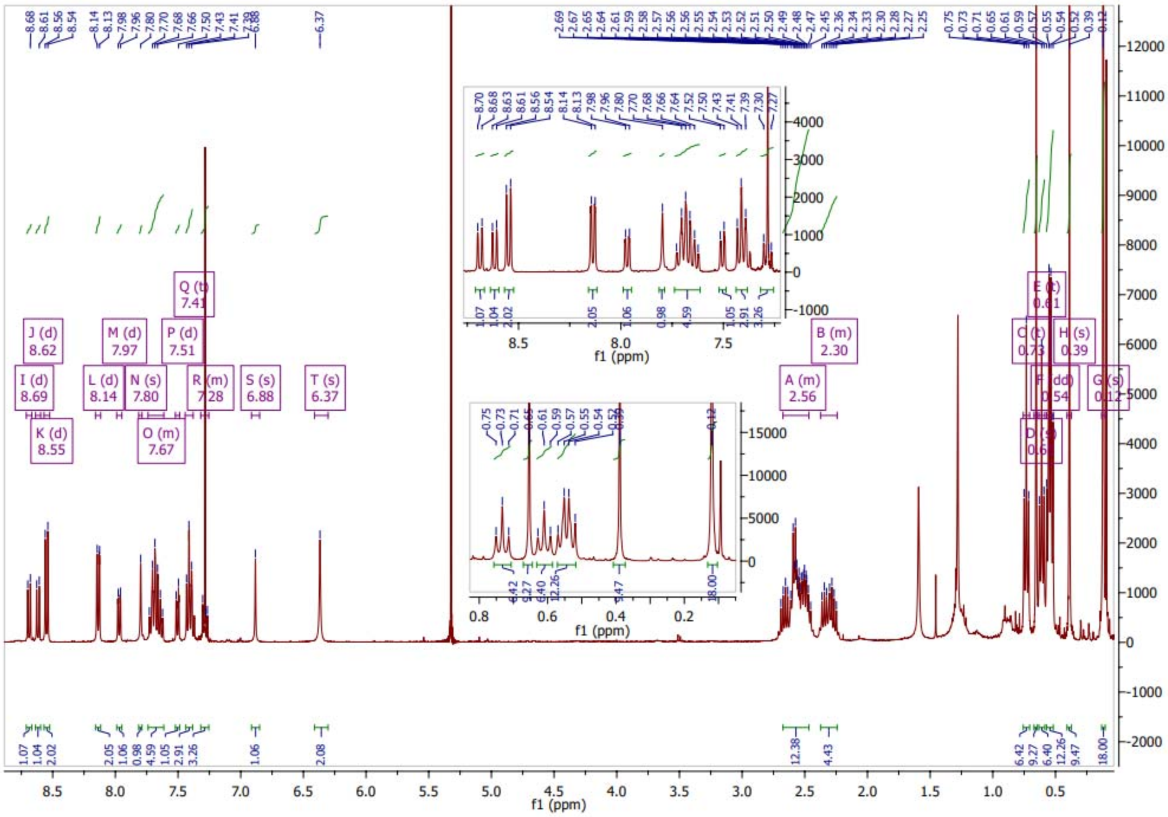


**Figure S74.** $^{1}$H NMR spectrum of *α₃β*-**3** (600 MHz, $CDCl_3$, 25°C).

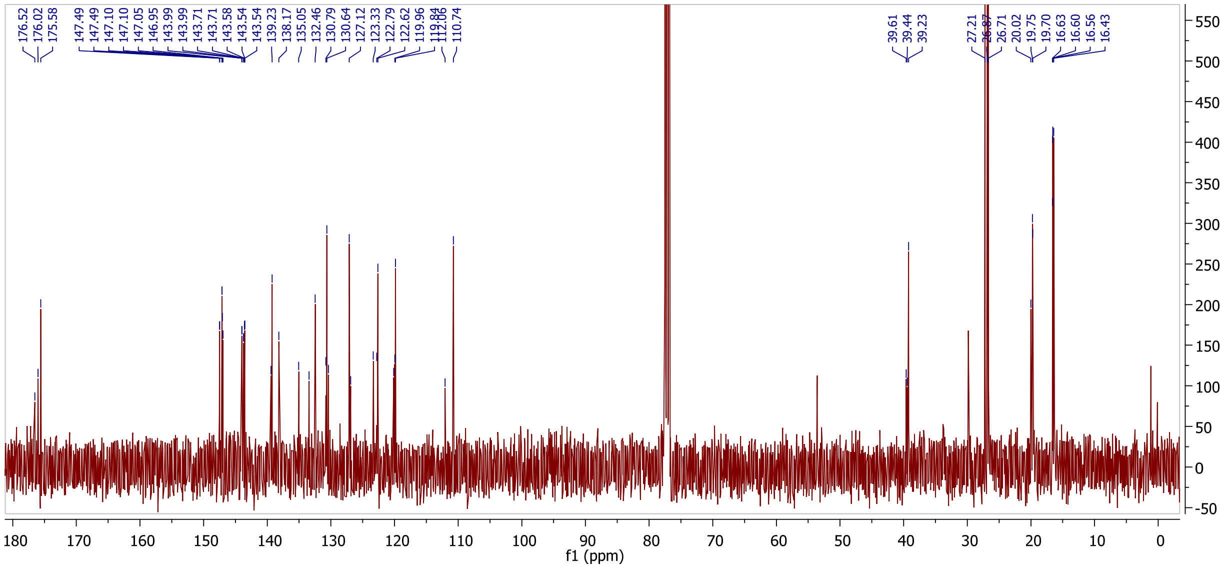


**Figure S75.** $^{13}$C NMR spectrum of *α₃β*-**3** (151 MHz, $CDCl_3$, 25°C).

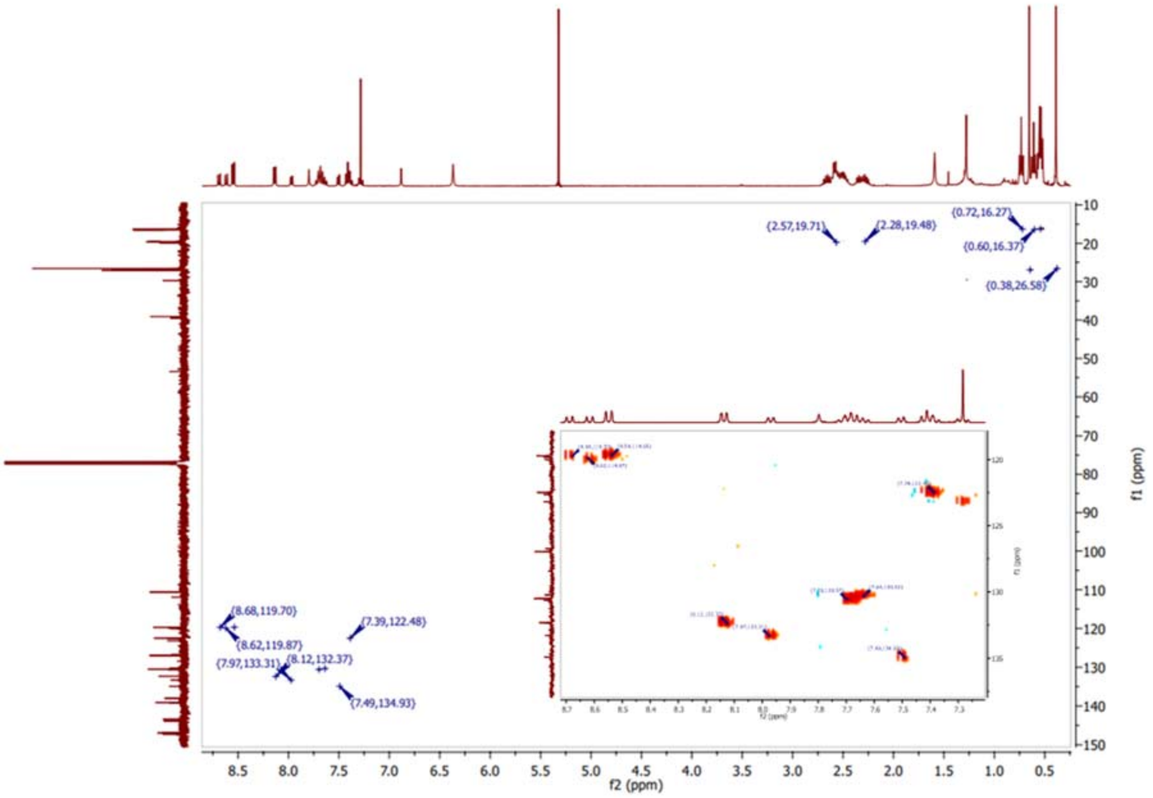


**Figure S76.** $^{1}H$–$^{13}C$ HSQC spectrum of *α₃β*-**3** with expansion of areas of interest ($CDCl_3$, 25°C).

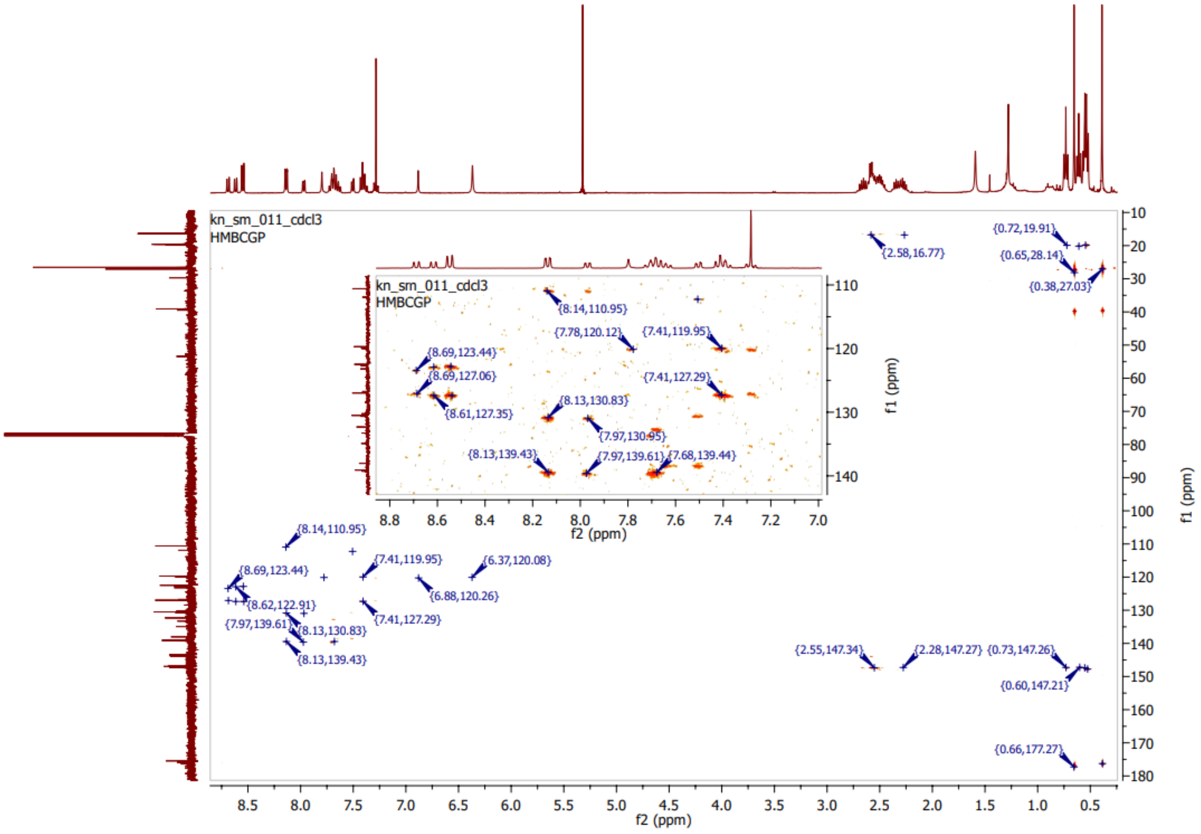


**Figure S77.** $^{1}H$–$^{13}C$ HMBC spectrum of *α₃β*-**3** with expansion of areas of interest ($CDCl_3$, 25°C).

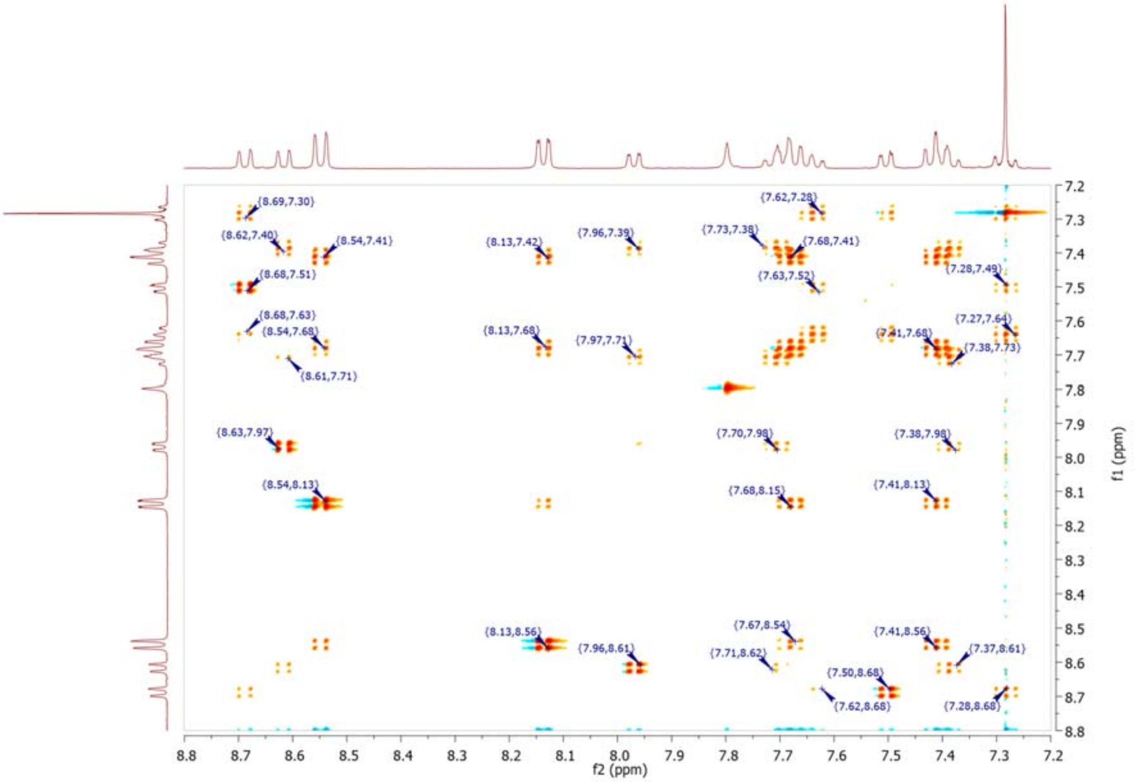


**Figure S78.** $^1H$–$^1H$ TOCSY spectrum of $\alpha_3\beta$-**3** with expansion of areas of interest ($CDCl_3$, 25°C).

**Elemental Composition Report** **Page 1**

**Single Mass Analysis**
Tolerance = 50.0 PPM / DBE: min = -1.5, max = 400.0
Element prediction: Off
Number of isotope peaks used for i-FIT = 5

Monoisotopic Mass, Odd and Even Electron Ions
68 formula(e) evaluated with 1 results within limits (up to 10 closest results for each mass)
Elements Used:
C: 0-80 H: 0-96 N: 0-8 O: 0-4 Ni: 0-1
Karolis Norvaisa (MSe), SM011
Q-TOF20210707GH003 25 (0.463) AM (Cen,8, 80.00, Ht,10000.0,1570.68,0.70); Sm (SG, 2x3.00); Sb (15,10.00 ); Cm (10:57) TOF MS LD+ 1.13e+003

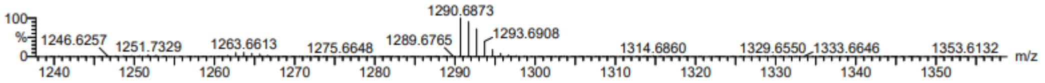


| | | | | | | | |
|---|---|---|---|---|---|---|---|
| Minimum: | | | | -1.5 | | | |
| Maximum: | | 5.0 | 50.0 | 400.0 | | | |
| Mass | Calc. Mass | mDa | PPM | DBE | i-FIT | i-FIT (Norm) | Formula |
| 1290.6873 | 1290.6908 | -3.5 | -2.7 | 37.5 | 61.7 | 0.0 | C80 H96 N8 O4 Ni |

**Figure S79.** HRMS (MALDI) of *α₃β*-**3**.

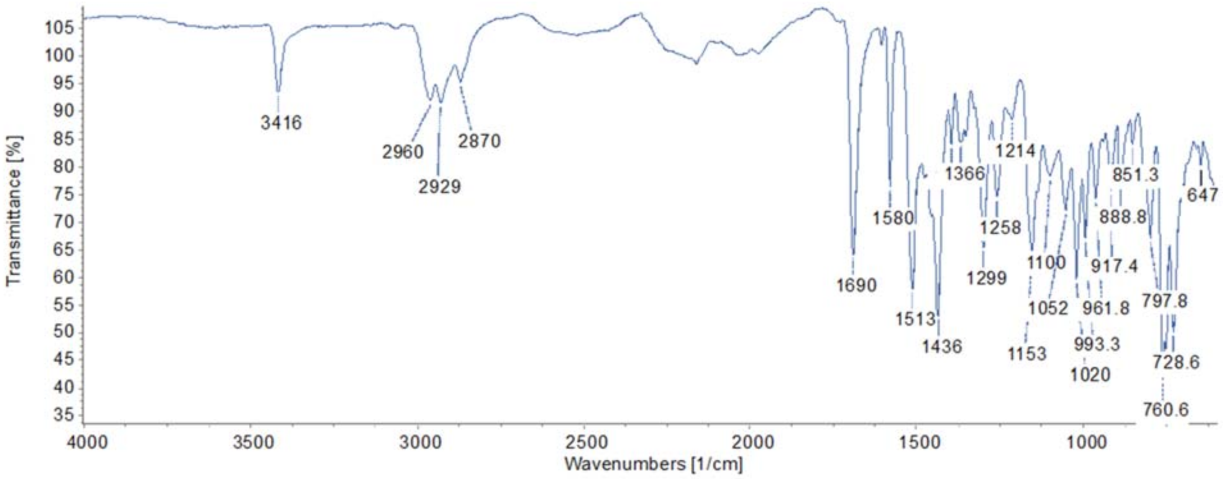


**Figure S80.** FTIR spectrum of *α₃β*-**3**.

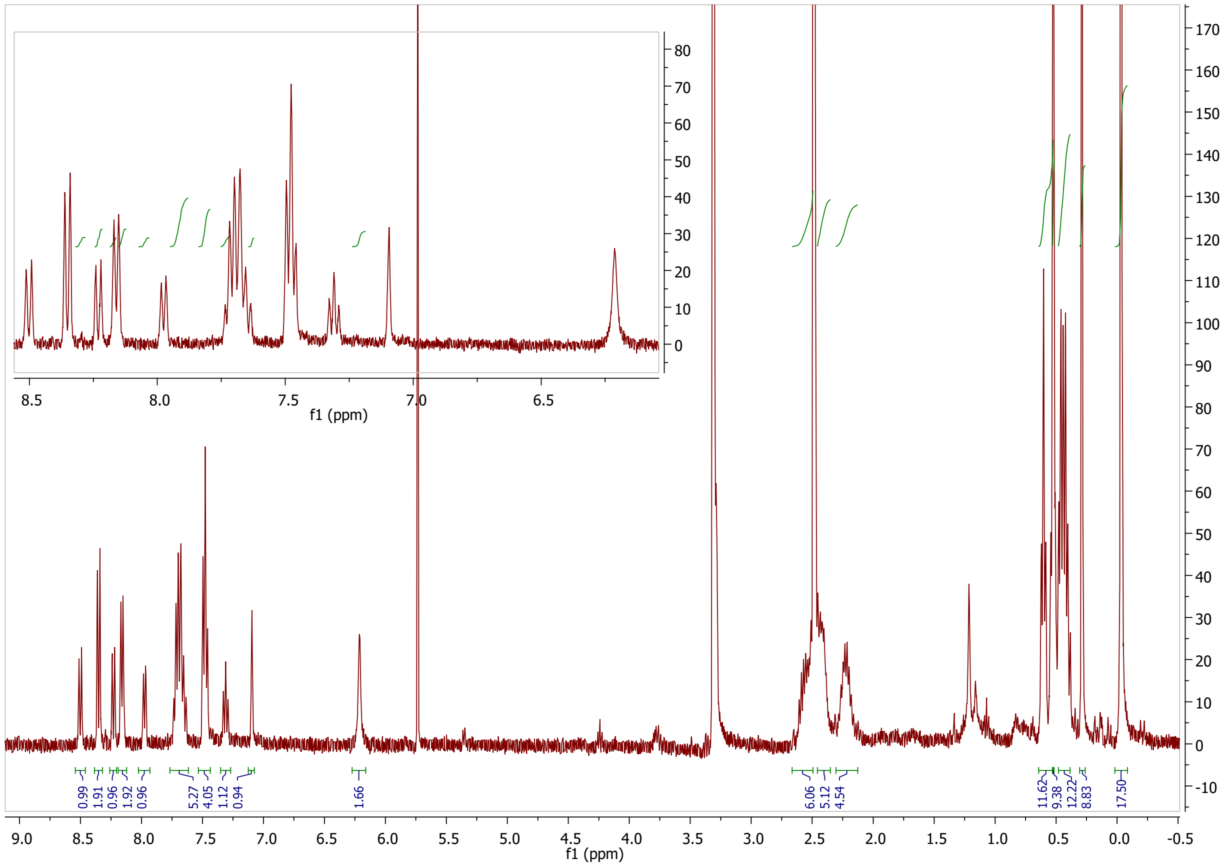

**Figure S81.** $^{1}$H NMR spectrum of *α₃β*-**3** (600 MHz, *d₆*-DMSO, 25°C).

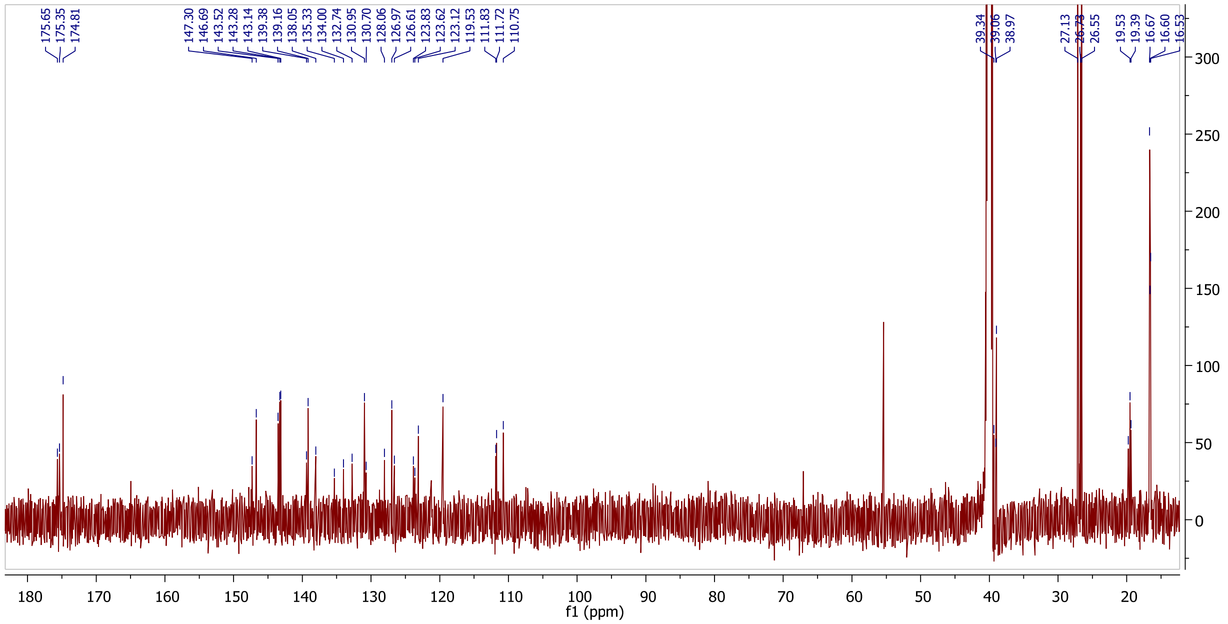

**Figure S82.** $^{13}$C NMR spectrum of *α₃β*-**3** (151 MHz, *d₆*-DMSO, 25°C).

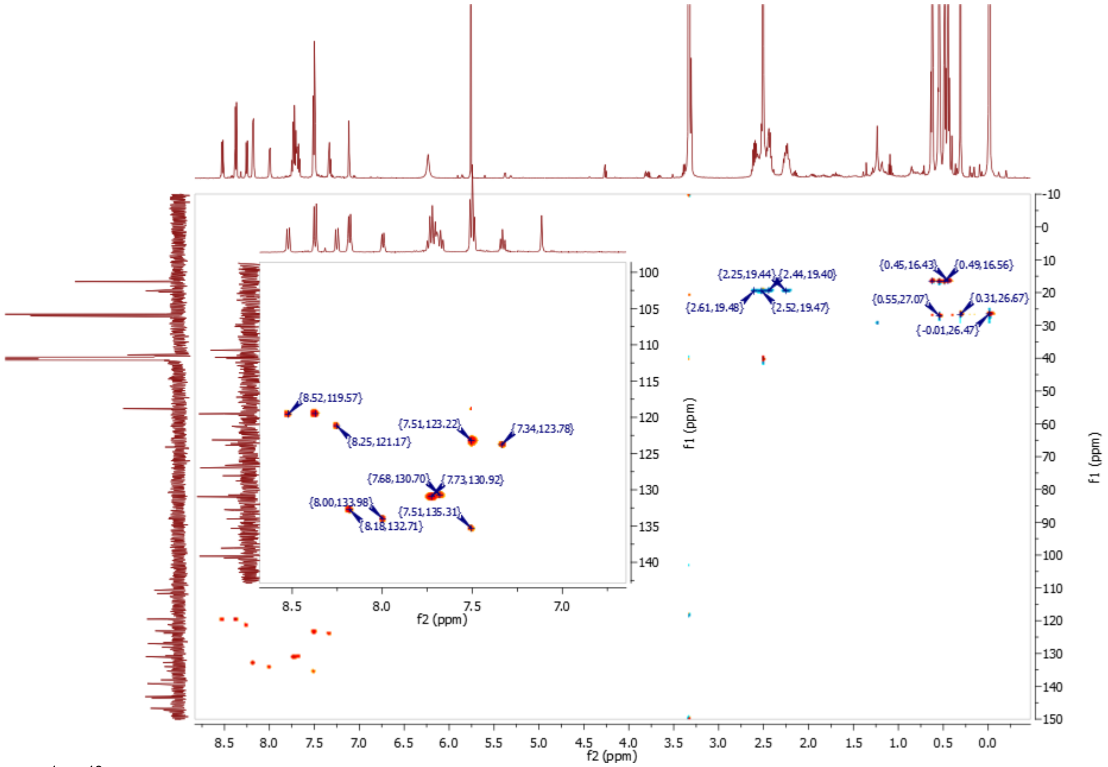

**Figure S83.** $^{1}$H–$^{13}$C HSQC spectrum of *α*$_3$*β*-**3** with expansion of areas of interest (*d*$_6$-DMSO, 25°C).

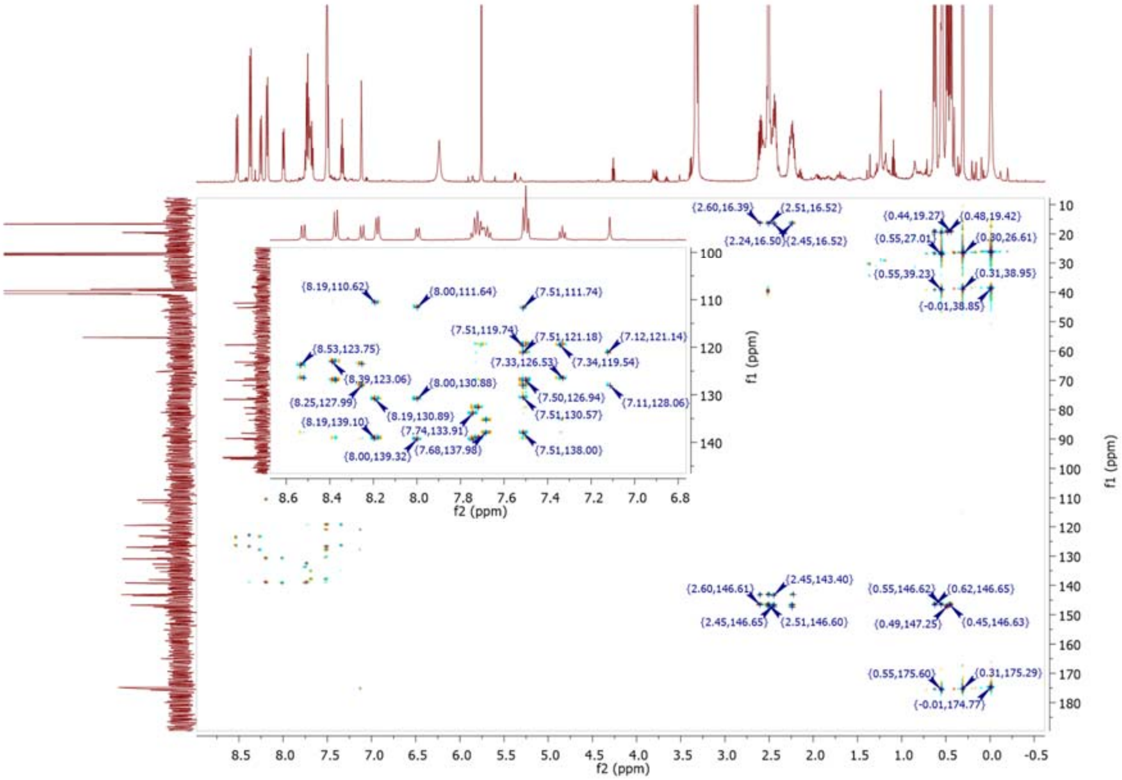

**Figure S84.** $^{1}$H–$^{13}$C HMBC spectrum of *α*$_3$*β*-**3** with expansion of areas of interest (*d*$_6$-DMSO, 25°C).

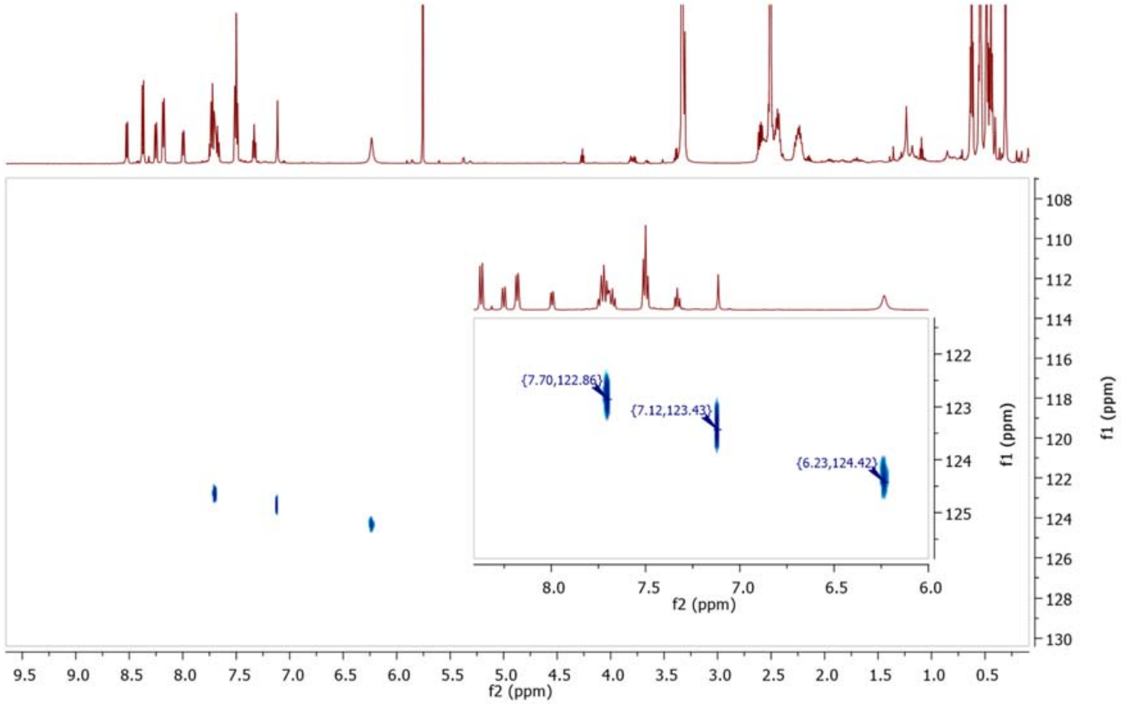


**Figure S85.** $^1H$–$^{15}N$ HSQC spectrum of *α₃β*-**3** with expansion of areas of interest (*d₆*-DMSO, 25°C).

## References


[1] H. Hope, *Prog. Inorg. Chem.* **2007**, *41*, 1–19.
[2] a) O. V. Dolomanov, L. J. Bourhis, R. J. Gildea, J. A. K. Howard, H. Puschmann, *J. Appl. Cryst.* **2009**, *42*, 339–341; b) G. Sheldrick, *Acta Crystallogr.* **2015**, *A71*, 3–8.
[3] A. Spek, *Acta Crystallogr.* **2015**, *C71*, 9–18.
[4] C. J. Kingsbury, M. O. Senge, *Coord. Chem. Rev.* **2021**, *431*, 213760–213783.
[5] V. Velkannan, P. Bhyrappa, *Polyhedron* **2015**, *87*, 170–180.
[6] K. Norvaiša, J. E. O'Brien, D. J. Gibbons, M. O. Senge, *Chem. Eur. J.* **2020**, *27*, 331–339.
[7] M. O. Senge, *Z. Naturforsch., B: Chem. Sci.* **2000**, *55*, 336–344.
[8] a) J. S. Lindsey, I. C. Schreiman, H. C. Hsu, P. C. Kearney, A. M. Marguerettaz, *J. Org. Chem.* **1987**, *52*, 827–836; b) J. L. Sessler, A. Mozattari, M. R. Johnson, *Org. Synth.* **1998**, *70*, 68–74.
[9] a) K. Norvaiša, K. J. Flanagan, D. Gibbons, M. O. Senge, *Angew. Chem.* **2019**, *131*, 16705–16709; *Angew. Chem. Int. Ed.* **2019**, *58*, 16553–16557.
[10] APEX3, Version 2016.9-0, Bruker AXS, Inc., Madison, WI, **2016**.
[11] SADABS, Version 2016/2, Bruker AXS, Inc., Madison, WI, **2014**.
[12] W. Jentzen, X.-Z. Song, J. A. Shelnutt, *J. Phys. Chem. B* **1997**, *101*, 1684–1699.
[13] L. Sun and J. A. Shelnutt, NSDGUI (Version 1.3 Alpha version), Sandia National Laboratory, Albuquerque, USA., **2000–2001**.
